# Nationally representative household survey data for studying the interaction between district-level development and individual-level socioeconomic gradients of cardiovascular disease risk factors in India

**DOI:** 10.1016/j.dib.2019.104486

**Published:** 2019-09-13

**Authors:** Lara Jung, Jan-Walter De Neve, Simiao Chen, Jennifer Manne-Goehler, Lindsay M. Jaacks, Daniel J. Corsi, Ashish Awasthi, S.V. Subramanian, Sebastian Vollmer, Till Bärnighausen, Pascal Geldsetzer

**Affiliations:** a Heidelberg Institute of Global Health, Heidelberg University, Heidelberg, Germany; b Division of Infectious Diseases, Massachusetts General Hospital, Harvard Medical School, Boston, MA, USA; c Department of Global Health and Population, Harvard T.H. Chan School of Public Health, Boston, MA, USA; d Public Health Foundation of India, New Delhi, Delhi NCR, India; e Ottawa Hospital Research Institute, Ottawa, ON, Canada; f School of Epidemiology and Public Health, University of Ottawa, Ottawa, ON, Canada; g Department of Social and Behavioral Sciences, Harvard T.H. Chan School of Public Health, Boston, MA, USA; h Department of Economics & Centre for Modern Indian Studies, University of Goettingen, Göttingen, Germany; i Africa Health Research Institute, Somkhele, KwaZulu-Natal, South Africa

**Keywords:** India, Cardiovascular disease, Education, Household wealth, Hypertension, Diabetes mellitus, Smoking, Obesity, Multi-level modelling, DLHS-4, District-Level Household Survey 4, AHS, Annual Health Survey, NFHS-4, National Family Health Survey, CVD, cardiovascular disease, SES, socio-economic status, PSU, primary sampling unit, CAB, Clinical, anthropometric, and biochemical

## Abstract

In this article, we describe the dataset used in our study entitled “The interaction between district-level development and individual-level socioeconomic gradients of cardiovascular disease risk factors in India: A cross-sectional study of 2.4 million adults”, recently published in Social Science & Medicine, and present supplementary analyses.

We used data from three different household surveys in India, which are representative at the district level. Specifically, we analyzed pooled data from the District-Level Household Survey 4 (DLHS-4) and the second update of the Annual Health Survey (AHS), and separately analyzed data from the National Family Health Survey (NFHS-4). The DLHS-4 and AHS sampled adults aged 18 years or older between 2012 and 2014, while the NFHS-4 sampled women aged 15–49 years and - in a subsample of 15% of households - men aged 15–54 years in 2015 and 2016.

The measures of individual-level socio-economic status that we used in both datasets were educational attainment and household wealth quintiles. The measures of district-level development, which we calculated from these data, were i) the percentage of participants living in an urban area, ii) female literacy rate, and iii) the district-level median of the continuous household wealth index. An additional measure of district-level development that we used was Gross Domestic Product per capita, which we obtained from the Planning Commission of the Government of India for 2004/2005.

Our outcome variables were diabetes, hypertension, obesity, and current smoking. The data were analyzed using both district-level regressions and multilevel modelling.

**Table:** Specifications Table

Subject	Public Health and Health Policy
Specific subject area	Cardiovascular disease; social epidemiology.
Type of data	Tables and figures
How data were acquired	The data are available at http://www.measuredhs.com (NFHS-4), http://www.iipsindia.ac.in (DLHS-4), and https://nrhm-mis.nic.in/hmisreports/AHSReports.aspx (AHS).
Data format	Analyzed and filtered
Parameters for data collection	Data from the DLHS-4 and AHS were pooled, whereas the NFHS-4 was analyzed separately. In the DLHS-4 and AHS, clinical, anthropometric, and biochemical (CAB) data were measured in all non-pregnant participants ≥ 18 years, whereas in the NFHS-4 biomarker tests were conducted among women aged 15–49 years and (in a random subsample of 15% of households) men aged 15–54 years. Multi-stage cluster random sampling was used to select participants.
Description of data collection	Socio-demographic characteristics were ascertained by administering questionnaires to all eligible women and men. Clinical, anthropometric, and biochemical (CAB) data were collected by trained personnel through biomarker tests and physical measurements.
Data source location	Combined, the DLHS-4 and AHS covered all states in India except Gujarat and Jammu and Kashmir, as well as all Union Territories except for Lakshadweep and Dadra and Nagar Haveli. The NFHS-4 covered all states and Union Territories.
Data accessibility	Tables and figures are presented in this article. Raw data and analysis code files are available in a repository. Repository name: Harvard Dataverse Direct URL to data: https://dataverse.harvard.edu/dataset.xhtml?persistentId=doi:10.7910/DVN/UVTMR5
Related research article	Lara Jung, Jan-Walter De Neve, Simiao Chen, Jennifer Manne-Goehler, Lindsay M. Jaacks, Daniel J. Corsi, Ashish Awasthi, S.V. Subramanian, Sebastian Vollmer, Till Bärnighausen, Pascal Geldsetzer. (2019) The interaction between district-level development and individual-level socioeconomic gradients of cardiovascular disease risk factors in India: A cross-sectional study of 2.4 million adults. Social Science & Medicine. https://doi.org/10.1016/j.socscimed.2019.112514

**Table:** 

**Value of the Data** • The data allow researchers and policy makers to examine how individual-level socio-economic gradients of cardiovascular disease risk factors are associated with district-level socio-economic development. • Insights gained from these analyses might give an indication as to how individual-level socio-economic gradients of cardiovascular disease risk factors will change in the future as districts continue to develop economically. • These data could be used to conduct analyses on socio-economic determinants of cardiovascular disease risk factors in India and merged with data from other countries to conduct analyses at a larger scale

## 1 Data

The provided data are supplementary data of the study entitled “The interaction between district-level development and individual-level socioeconomic gradients of cardiovascular disease risk factors in India: A cross-sectional study of 2.4 million adults”, which was recently published in Social Science & Medicine [1].

Table 1, Table 2 report unweighted sample characteristics for the data, stratified by gender.

**Table 1:** Sample characteristics stratified by gender (NFHS-4).^a^^,^^b^^,^^c^

Characteristic	Female	Male
No. (%)	647,451 (85.5)	110,204 (14.5)
**CVD risk factors**		
Diabetes, No. (%)	17,246 (2.7)	4351 (4.1)
*missing*	19,298 (3.0)	4430 (4.0)
High blood glucose, No. (%)	11,138 (1.8)	2972 (2.8)
*missing*	19,298 (3.0)	4430 (4.0)
Hypertension, No. (%)	111,144 (17.5)	22,690 (21.2)
*missing*	11,749 (1.8)	3320 (3.0)
High blood pressure, No. (%)	66,215 (10.4)	17,714 (16.6)
*missing*	11,727 (1.8)	3317 (3.0)
BMI, No. (%)		
<18.5 kg/mˆ2	141,669 (22.3)	20,446 (19.1)
18.5–<23 kg/mˆ2	295,713 (46.5)	50,768 (47.5)
23–<25 kg/mˆ2	80,849 (12.7)	16,753 (15.7)
25–<30 kg/mˆ2	90,422 (14.2)	16,014 (15.0)
≥30 kg/mˆ2	27,696 (4.4)	2914 (2.7)
*missing*	11,102 (1.7)	3309 (3.0)
BMI>27.5 kg/mˆ2, No. (%)	58,868 (9.3)	7678 (7.2)
Currently smoking tobacco, No. (%)	7923 (1.2)	29,996 (27.2)
*missing*	0 (0.0)	0 (0.0)
**Sociodemographic characteristics**		
Age group, No. (%), Y		
15–19	117,259 (18.1)	18,710 (17.0)
20–24	103,149 (15.9)	16,182 (14.7)
25–29	100,533 (15.5)	15,798 (14.3)
30–34	90,854 (14.0)	14,349 (13.0)
35–39	87,876 (13.6)	13,693 (12.4)
40–44	75,671 (11.7)	11,848 (10.8)
45–49	72,109 (11.1)	11,088 (10.1)
50–54	–	8536 (7.7)
55–59	–	–
60–64	–	–
>65	–	–
*missing*	0 (0.0)	0 (0.0)
Mean age, y (SD)	30.22 (9.91)	31.80 (11.10)
Urban area, No. (%)	191,482 (29.6)	35,072 (31.8)
Education, No. (%)		
Below primary education	223,076 (34.5)	22,040 (20.0)
Primary	43,404 (6.7)	6978 (6.3)
Some secondary	253,067 (39.1)	51,625 (46.8)
Secondary completed	55,495 (8.6)	12,475 (11.3)
Higher	72,409 (11.2)	17,086 (15.5)
*missing*	0 (0.0)	0 (0.0)
Literate, No. (%)	436,969 (67.5)	92,551 (84.0)
*missing*	0 (0.0)	0 (0.0)
Household wealth quintile computed for each district, No. (%)		
Q1 (Poorest)	130,131 (20.1)	21,886 (19.9)
Q2	129,899 (20.1)	21,636 (19.6)
Q3	129,712 (20.0)	21,828 (19.8)
Q4	129,339 (20.0)	22,196 (20.1)
Q5 (Richest)	128,370 (19.8)	22,658 (20.6)
*missing*	0 (0.0)	0 (0.0)
Household wealth quintile computed nationally, No. (%)		
Q1 (Poorest)	120,310 (18.6)	19,013 (17.3)
Q2	128,715 (19.9)	21,380 (19.4)
Q3	133,429 (20.6)	22,521 (20.4)
Q4	130,721 (20.2)	23,147 (21.0)
Q5 (Richest)	134,276 (20.7)	24,143 (21.9)
*missing*	0 (0.0)	0 (0.0)

**Abbreviations:** No. = number; % = Percentage; BMI=Body Mass Index; y = years; SD=Standard deviation; Q = Quintile.

a Sample characteristics were not weighted using sampling weights.

b Percentages shown were calculated after excluding those with a missing value for the relevant variable.

c Household wealth quintile (computed within a district) for this table was created separately for rural and urban areas in each district.

**Table 2:** Sample characteristics stratified by gender (DLHS-4/AHS).^a^^,^^b^^,^^c^

Characteristic	Female	Male
No. (%)	771,995 (47.7)	846,287 (52.3)
**CVD risk factors**		
Diabetes, No. (%)	54,846 (7.6)	50,810 (8.0)
*missing*	54,004 (7.0)	210,901 (24.9)
Hypertension, No. (%)	183,995 (24.8)	194,929 (29.4)
*missing*	29,379 (3.8)	184,066 (21.7)
BMI, No. (%)		
<18.5 kg/mˆ2	150,474 (20.3)	118,746 (17.9)
18.5–<23 kg/mˆ2	339,657 (45.9)	324,399 (49.0)
23–<25 kg/mˆ2	102,133 (13.8)	104,813 (15.8)
25–<30 kg/mˆ2	110,122 (14.9)	92,113 (13.9)
≥30 kg/mˆ2	38,183 (5.2)	21,822 (3.3)
*missing*	31,426 (4.1)	184,394 (21.8)
BMI>27.5 kg/mˆ2, No. (%)	76,245 (10.3)	49,516 (7.5)
Currently smoking tobacco, No. (%)	14,610 (2.3)	140,083 (23.1)
*missing*	129,159 (16.7)	238,928 (28.2)
**Sociodemographic characteristics**		
Age group, No. (%), y		
15–19	37,302 (4.8)	46,934 (5.5)
20–24	89,034 (11.5)	108,601 (12.8)
25–29	94,440 (12.2)	99,460 (11.8)
30–34	92,183 (11.9)	92,793 (11.0)
35–39	89,418 (11.6)	87,600 (10.4)
40–44	79,676 (10.3)	84,287 (10.0)
45–49	68,202 (8.8)	74,833 (8.8)
50–54	62,045 (8.0)	64,969 (7.7)
55–59	46,767 (6.1)	52,287 (6.2)
60–64	40,888 (5.3)	47,226 (5.6)
>65	72,028 (9.3)	87,271 (10.3)
*missing*	12 (0.0)	26 (0.0)
Mean age, y (SD)	40.66 (15.65)	40.80 (16.22)
Urban area, No. (%)	250,952 (32.5)	284,567 (33.6)
Education, No. (%)		
Below primary education	363,801 (47.3)	232,186 (27.6)
Primary	91,282 (11.9)	107,130 (12.7)
Some secondary	194,321 (25.3)	285,337 (33.9)
Secondary completed	61,236 (8.0)	103,680 (12.3)
Higher	58,266 (7.6)	113,732 (13.5)
*missing*	3089 (0.4)	4222 (0.5)
Literate, No. (%)	479,727 (62.4)	689,709 (81.9)
*missing*	3089 (0.4)	4222 (0.5)
Household wealth quintile computed for each district, No. (%)		
Q1 (Poorest)	149,860 (20.3)	160,202 (19.7)
Q2	147,637 (20.0)	162,012 (20.0)
Q3	147,104 (20.0)	162,491 (20.0)
Q4	146,859 (19.9)	162,801 (20.1)
Q5 (Richest)	145,563 (19.8)	163,674 (20.2)
*missing*	34,972 (4.5)	35,107 (4.1)
Household wealth quintile computed for each district, No. (%)		
Q1 (Poorest)	151,347 (20.5)	154,351 (19.0)
Q2	145,350 (19.7)	157,755 (19.4)
Q3	143,078 (19.4)	159,925 (19.7)
Q4	147,126 (20.0)	167,379 (20.6)
Q5 (Richest)	150,122 (20.4)	171,770 (21.2)
*missing*	34,972 (4.5)	35,107 (4.1)

**Abbreviations:** No. = number; % = Percentage; BMI=Body Mass Index; y = years; Q = Quintile.

a Sample characteristics were not weighted using sampling weights.

b Percentages shown were calculated after excluding those with a missing value for the relevant variable.

c Household wealth quintile (computed within a district) for this table was created separately for rural and urban areas in each district.

Fig. 1a, Fig. 1b, Fig. 1c, Fig. 1d, Fig. 2a, Fig. 2b, Fig. 2c, Fig. 2d display the association of a district's development with the difference in the probability of having hypertension between most and least educated categories (i.e., having completed secondary school or a tertiary education versus not having completed primary school) (Fig. 1a, Fig. 1b, Fig. 1c, Fig. 1d) or between the top two and bottom two household wealth quintiles computed for each district (Fig. 2a, Fig. 2b, Fig. 2c, Fig. 2d). We used the following indicators of district-level socio-economic development: median household wealth (Fig. 1a, Fig. 2aa), GDP per capita (Fig. 1b, Fig. 2bb), percentage of participants living in an urban area (Fig. 1c, Fig. 2cc) and female literacy rate (Fig. 1d, Fig. 2dd. We also show the same analyses for the following CVD risk factors: obesity (Fig. 3a, Fig. 3b, Fig. 3c, Fig. 3d, Fig. 4a, Fig. 4b, Fig. 4c, Fig. 4dd), diabetes (Fig. 5a, Fig. 5b, Fig. 5c, Fig. 5d, Fig. 6a, Fig. 6b, Fig. 6c, Fig. 6dd), and currently smoking (Fig. 7a, Fig. 7b, Fig. 7c, Fig. 7d, Fig. 8a, Fig. 8b, Fig. 8c, Fig. 8dd). In Fig. 9a, Fig. 9b, Fig. 9c, Fig. 9da–d, we compare top and bottom household wealth quintiles computed for each district (for district-level primary school completion rate only). In Fig. 10a, Fig. 10b, Fig. 10c, Fig. 10da–d we examine the association of a district's primary school completion rate, with the difference in the probability of a CVD risk factor between the top two and bottom two household wealth quintiles computed nationally. The numbers of districts included in the district-level regressions for each risk factor and SES measure are presented in Table 3.

**Fig. 1a:**
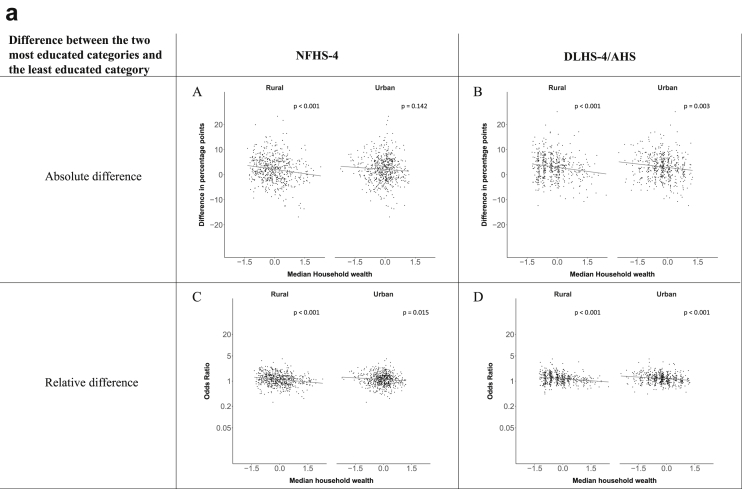
**Hypertension: association of district-level median household wealth with the difference between completing at least secondary school and less than primary school.** The points in the plot represent the regression coefficient from a linear probability model (for the absolute difference) and the Odds Ratio from a logistic regression (for the relative difference) comparing those participants who completed at least secondary school to those who did not complete primary school education in a district. These regressions regressed hypertension onto sex, age, and urban/rural residency separately for each district. The analysis included 595 districts in the NFHS-4 and 516 districts in the DLHS-4/AHS. The grey line through the scatterplots has been fitted using ordinary least squares regression (with each data point in the plot having the same weight). The p-value shows whether the slope of the grey line is significantly different from zero. The y-axis for the relative difference is on the logarithmic scale.

**Fig. 1b:**
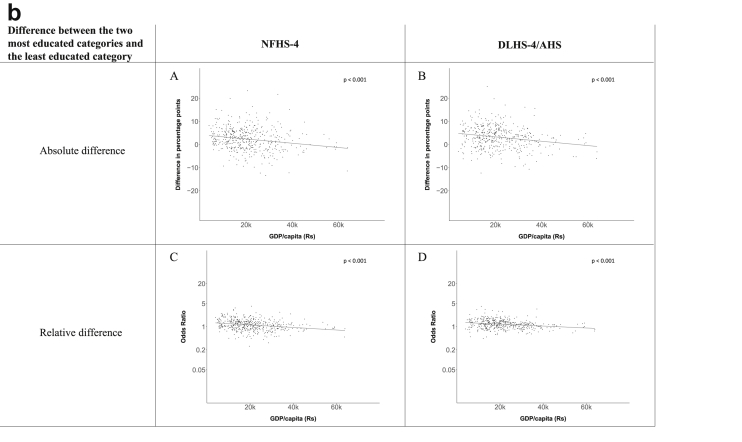
**Hypertension: association of a district's GDP/capita with the difference between completing at least secondary school and less than primary school.** The points in the plot represent the regression coefficient from a linear probability model (for the absolute difference) and the Odds Ratio from a logistic regression (for the relative difference) comparing those participants who completed at least secondary school to those who did not complete primary school education in a district. These regressions regressed hypertension onto sex, age, and urban/rural residency separately for each district. The analysis included 450 districts in the NFHS-4 and 436 districts in the DLHS-4/AHS. The grey line through the scatterplots has been fitted using ordinary least squares regression (with each data point in the plot having the same weight). The p-value shows whether the slope of the grey line is significantly different from zero. The y-axis for the relative difference is on the logarithmic scale.

**Fig. 1c:**
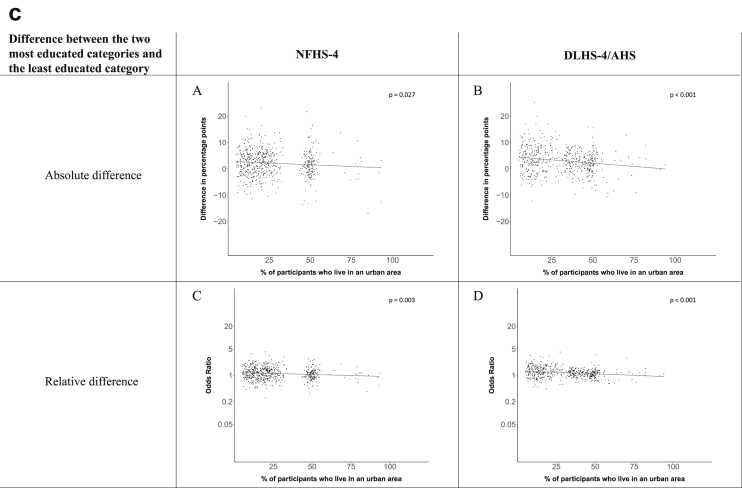
**Hypertension: association of a district's urban population with the difference between completing at least secondary school and less than primary school.** The points in the plot represent the regression coefficient from a linear probability model (for the absolute difference) and the Odds Ratio from a logistic regression (for the relative difference) comparing those participants who completed at least secondary school to those who did not complete primary school education in a district. These regressions regressed hypertension onto sex, age, and urban/rural residency separately for each district. The analysis included 595 districts in the NFHS-4 and 516 districts in the DLHS-4/AHS. The grey line through the scatterplots has been fitted using ordinary least squares regression (with each data point in the plot having the same weight). The p-value shows whether the slope of the grey line is significantly different from zero. The y-axis for the relative difference is on the logarithmic scale.

**Fig. 1d:**
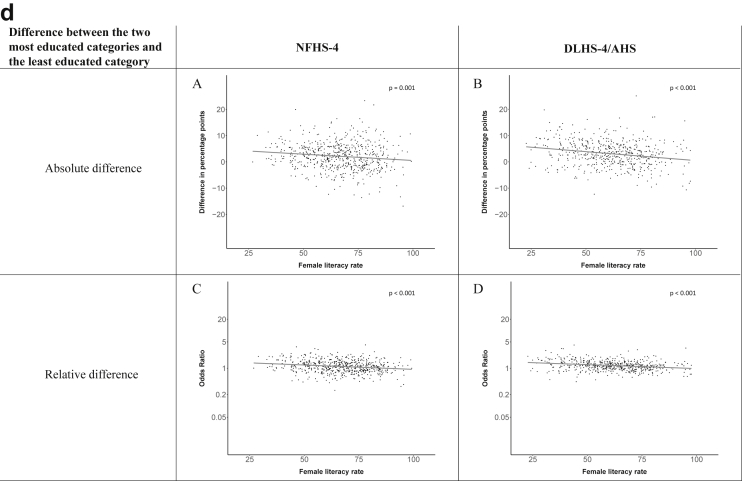
**Hypertension: association of district-level female literacy with the difference between completing at least secondary school and less than primary school.** The points in the plot represent the regression coefficient from a linear probability model (for the absolute difference) and the Odds Ratio from a logistic regression (for the relative difference) comparing those participants who completed at least secondary school to those who did not complete primary school education in a district. These regressions regressed hypertension onto sex, age, and urban/rural residency separately for each district. The analysis included 595 districts in the NFHS-4 and 516 districts in the DLHS-4/AHS. The grey line through the scatterplots has been fitted using ordinary least squares regression (with each data point in the plot having the same weight). The p-value shows whether the slope of the grey line is significantly different from zero. The y-axis for the relative difference is on the logarithmic scale.

**Fig. 2a:**
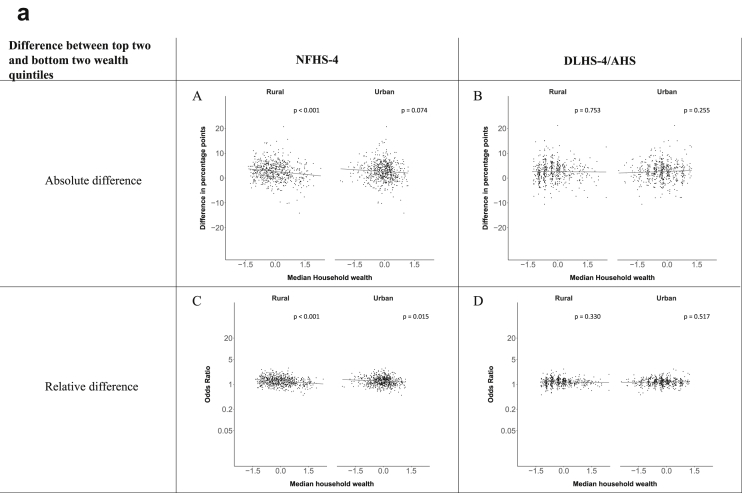
**Hypertension: association of district-level median household wealth with the difference between the top two and bottom two household wealth quintiles computed for each district.** The points in the plot represent the regression coefficient from a linear probability model (for the absolute difference) and the Odds Ratio from a logistic regression (for the relative difference) comparing the richest to the poorest household wealth quintile in a district. These regressions regressed hypertension onto sex, age, and urban/rural residency separately for each district. The analysis included 608 districts in the NFHS-4 and 517 districts in the DLHS-4/AHS. The grey line through the scatterplots has been fitted using ordinary least squares regression (with each data point in the plot having the same weight). The p-value shows whether the slope of the grey line is significantly different from zero. The y-axis for the relative difference is on the logarithmic scale.

**Fig. 2b:**
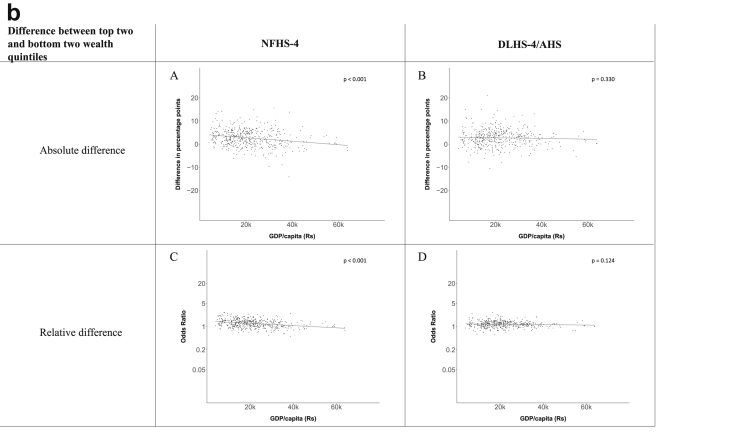
**Hypertension: association of a district's GDP/capita with the difference between the top two and bottom two household wealth quintiles computed for each district.** The points in the plot represent the regression coefficient from a linear probability model (for the absolute difference) and the Odds Ratio from a logistic regression (for the relative difference) comparing the richest to the poorest household wealth quintile in a district. These regressions regressed hypertension onto sex, age, and urban/rural residency separately for each district. The analysis included 462 districts in the NFHS-4 and 437 districts in the DLHS-4/AHS. The grey line through the scatterplots has been fitted using ordinary least squares regression (with each data point in the plot having the same weight). The p-value shows whether the slope of the grey line is significantly different from zero. The y-axis for the relative difference is on the logarithmic scale.

**Fig. 2c:**
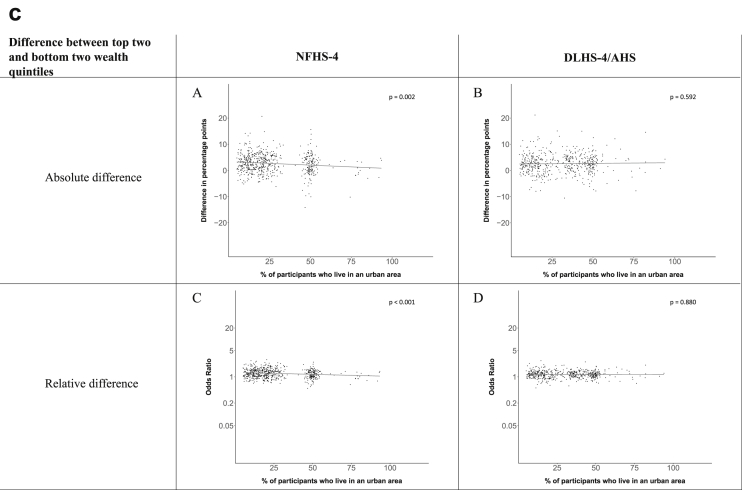
**Hypertension: association of a district's urban population with the difference between the top two and bottom two household wealth quintiles computed for each district.** The points in the plot represent the regression coefficient from a linear probability model (for the absolute difference) and the Odds Ratio from a logistic regression (for the relative difference) comparing the richest to the poorest household wealth quintile in a district. These regressions regressed hypertension onto sex, age, and urban/rural residency separately for each district. The analysis included 608 districts in the NFHS-4 and 517 districts in the DLHS-4/AHS. The grey line through the scatterplots has been fitted using ordinary least squares regression (with each data point in the plot having the same weight). The p-value shows whether the slope of the grey line is significantly different from zero. The y-axis for the relative difference is on the logarithmic scale.

**Fig. 2d:**
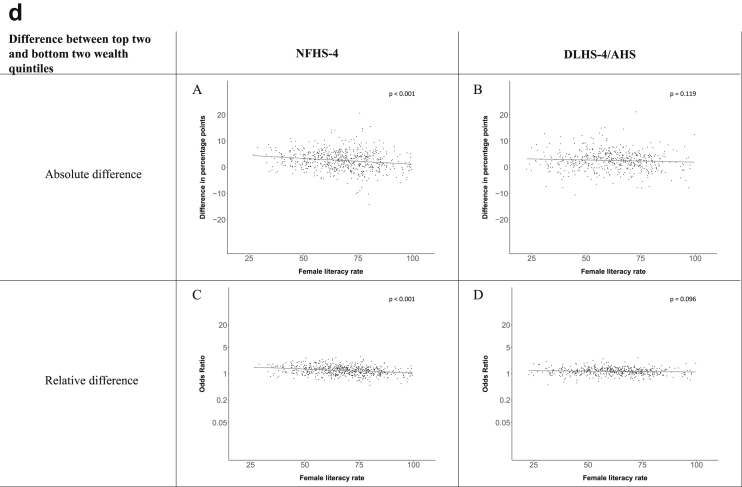
**Hypertension: association district-level female literacy with the difference between the top two and bottom two household wealth quintiles computed for each district.** The points in the plot represent the regression coefficient from a linear probability model (for the absolute difference) and the Odds Ratio from a logistic regression (for the relative difference) comparing the richest to the poorest household wealth quintile in a district. These regressions regressed hypertension onto sex, age, and urban/rural residency separately for each district. The analysis included 608 districts in the NFHS-4 and 517 districts in the DLHS-4/AHS. The grey line through the scatterplots has been fitted using ordinary least squares regression (with each data point in the plot having the same weight). The p-value shows whether the slope of the grey line is significantly different from zero. The y-axis for the relative difference is on the logarithmic scale.

**Fig. 3a:**
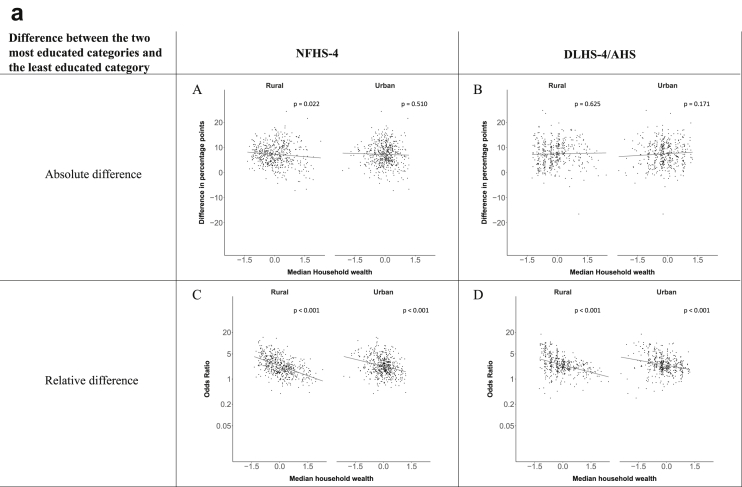
**Obesity: association of district-level median household wealth with the difference between completing at least secondary school and less than primary school.** The points in the plot represent the regression coefficient from a linear probability model (for the absolute difference) and the Odds Ratio from a logistic regression (for the relative difference) comparing those participants who completed at least secondary school to those who did not complete primary school education in a district. These regressions regressed obesity onto sex, age, and urban/rural residency separately for each district. The analysis included 531 districts in the NFHS-4 and 443 districts in the DLHS-4/AHS. The grey line through the scatterplots has been fitted using ordinary least squares regression (with each data point in the plot having the same weight). The p-value shows whether the slope of the grey line is significantly different from zero. The y-axis for the relative difference is on the logarithmic scale.

**Fig. 3b:**
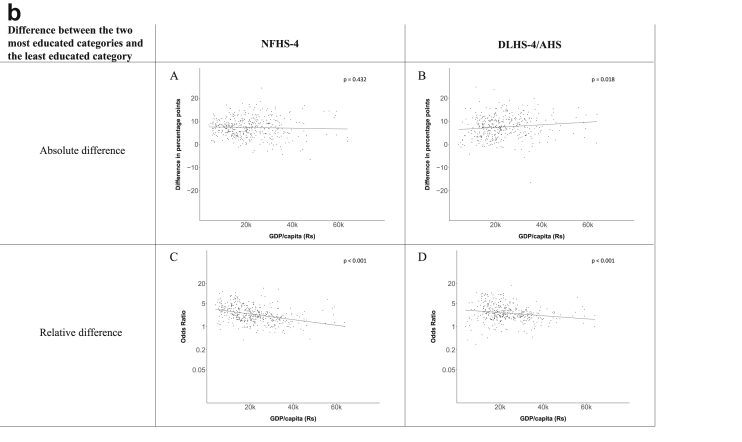
**Obesity: association of a district's GDP/capita with the difference between completing at least secondary school and less than primary school.** The points in the plot represent the regression coefficient from a linear probability model (for the absolute difference) and the Odds Ratio from a logistic regression (for the relative difference) comparing those participants who completed at least secondary school to those who did not complete primary school education in a district. These regressions regressed obesity onto sex, age, and urban/rural residency separately for each district. The analysis included 407 districts in the NFHS-4 and 376 districts in the DLHS-4/AHS. The grey line through the scatterplots has been fitted using ordinary least squares regression (with each data point in the plot having the same weight). The p-value shows whether the slope of the grey line is significantly different from zero. The y-axis for the relative difference is on the logarithmic scale.

**Fig. 3c:**
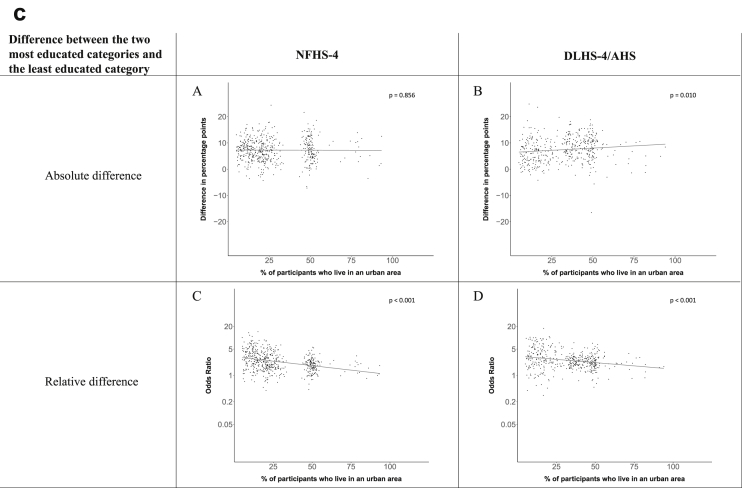
**Obesity: association of a district's urban population with the difference between completing at least secondary school and less than primary school.** The points in the plot represent the regression coefficient from a linear probability model (for the absolute difference) and the Odds Ratio from a logistic regression (for the relative difference) comparing those participants who completed at least secondary school to those who did not complete primary school education in a district. These regressions regressed obesity onto sex, age, and urban/rural residency separately for each district. The analysis included 531 districts in the NFHS-4 and 443 districts in the DLHS-4/AHS. The grey line through the scatterplots has been fitted using ordinary least squares regression (with each data point in the plot having the same weight). The p-value shows whether the slope of the grey line is significantly different from zero. The y-axis for the relative difference is on the logarithmic scale.

**Fig. 3d:**
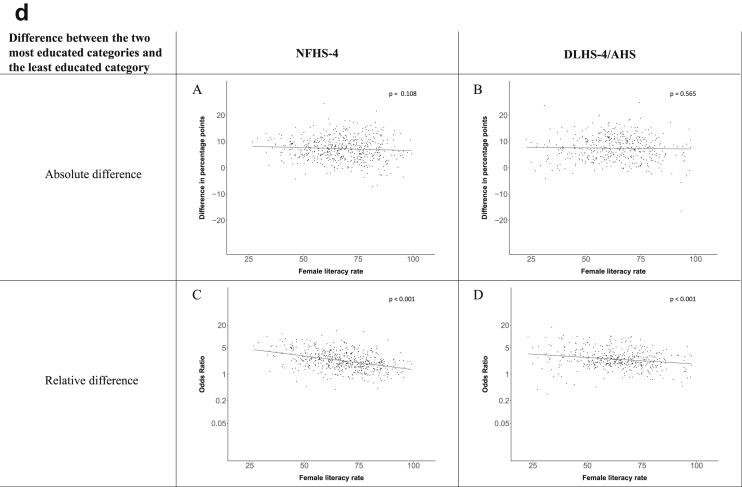
**Obesity: association of district-level female literacy with the difference between completing at least secondary school and less than primary school.** The points in the plot represent the regression coefficient from a linear probability model (for the absolute difference) and the Odds Ratio from a logistic regression (for the relative difference) comparing those participants who completed at least secondary school to those who did not complete primary school education in a district. These regressions regressed obesity onto sex, age, and urban/rural residency separately for each district. The analysis included 531 districts in the NFHS-4 and 443 districts in the DLHS-4/AHS. The grey line through the scatterplots has been fitted using ordinary least squares regression (with each data point in the plot having the same weight). The p-value shows whether the slope of the grey line is significantly different from zero. The y-axis for the relative difference is on the logarithmic scale.

**Fig. 4a:**
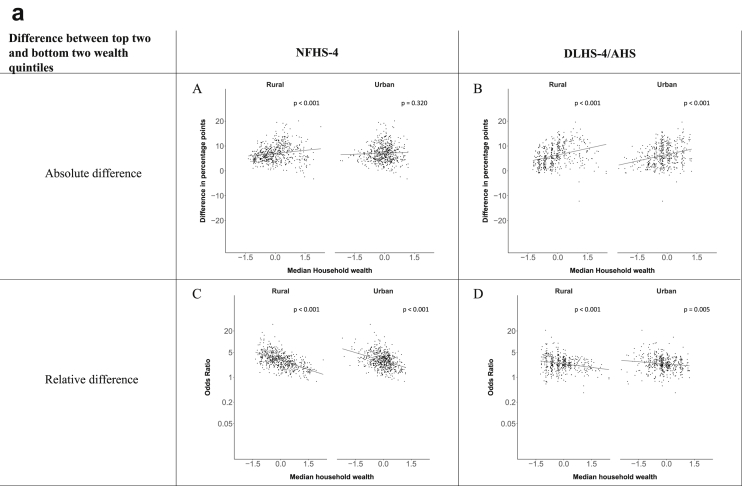
**Obesity: association of district-level median household wealth with the difference between the top two and bottom two household wealth quintiles computed for each district.** The points in the plot represent the regression coefficient from a linear probability model (for the absolute difference) and the Odds Ratio from a logistic regression (for the relative difference) comparing the richest to the poorest household wealth quintile in a district. These regressions regressed obesity onto sex, age, and urban/rural residency separately for each district. The analysis included 589 districts in the NFHS-4 and 461 districts in the DLHS-4/AHS. The grey line through the scatterplots has been fitted using ordinary least squares regression (with each data point in the plot having the same weight). The p-value shows whether the slope of the grey line is significantly different from zero. The y-axis for the relative difference is on the logarithmic scale.

**Fig. 4b:**
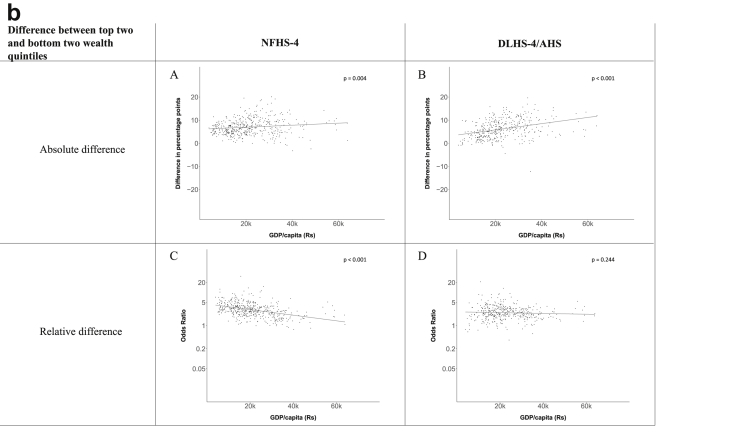
**Obesity: association of a district's GDP/capita with the difference between the top two and bottom two household wealth quintiles computed for each district.** The points in the plot represent the regression coefficient from a linear probability model (for the absolute difference) and the Odds Ratio from a logistic regression (for the relative difference) comparing the richest to the poorest household wealth quintile in a district. These regressions regressed obesity onto sex, age, and urban/rural residency separately for each district. The analysis included 454 districts in the NFHS-4 and 389 districts in the DLHS-4/AHS. The grey line through the scatterplots has been fitted using ordinary least squares regression (with each data point in the plot having the same weight). The p-value shows whether the slope of the grey line is significantly different from zero. The y-axis for the relative difference is on the logarithmic scale.

**Fig. 4c:**
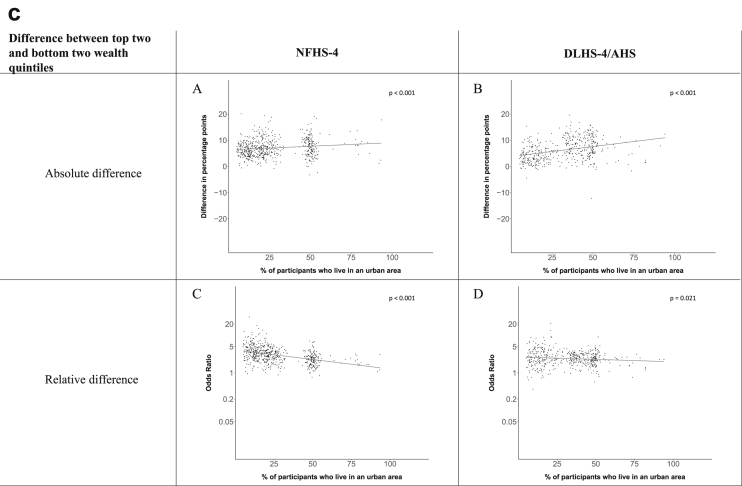
**Obesity: association of a district's urban population with the difference between the top two and bottom two household wealth quintiles computed for each district.** The points in the plot represent the regression coefficient from a linear probability model (for the absolute difference) and the Odds Ratio from a logistic regression (for the relative difference) comparing the richest to the poorest household wealth quintile in a district. These regressions regressed obesity onto sex, age, and urban/rural residency separately for each district. The analysis included 589 districts in the NFHS-4 and 461 districts in the DLHS-4/AHS. The grey line through the scatterplots has been fitted using ordinary least squares regression (with each data point in the plot having the same weight). The p-value shows whether the slope of the grey line is significantly different from zero. The y-axis for the relative difference is on the logarithmic scale.

**Fig. 4d:**
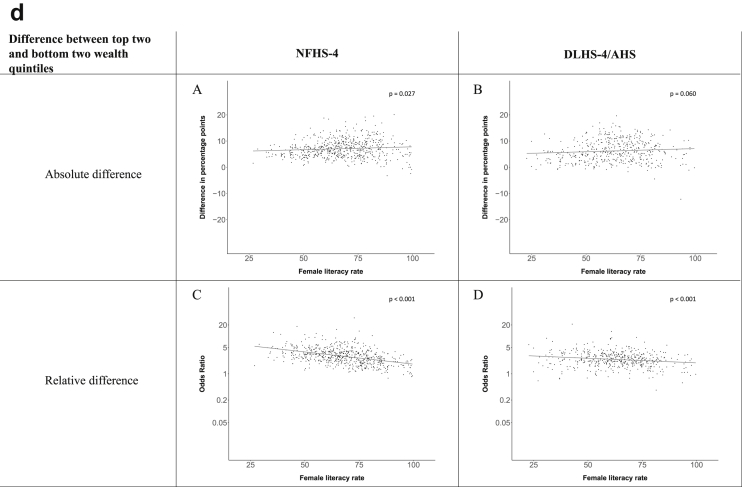
**Obesity: association district-level female literacy with the difference between the top two and bottom two household wealth quintiles computed for each district.** The points in the plot represent the regression coefficient from a linear probability model (for the absolute difference) and the Odds Ratio from a logistic regression (for the relative difference) comparing the richest to the poorest household wealth quintile in a district. These regressions regressed obesity onto sex, age, and urban/rural residency separately for each district. The analysis included 589 districts in the NFHS-4 and 461 districts in the DLHS-4/AHS. The grey line through the scatterplots has been fitted using ordinary least squares regression (with each data point in the plot having the same weight). The p-value shows whether the slope of the grey line is significantly different from zero. The y-axis for the relative difference is on the logarithmic scale.

**Fig. 5a:**
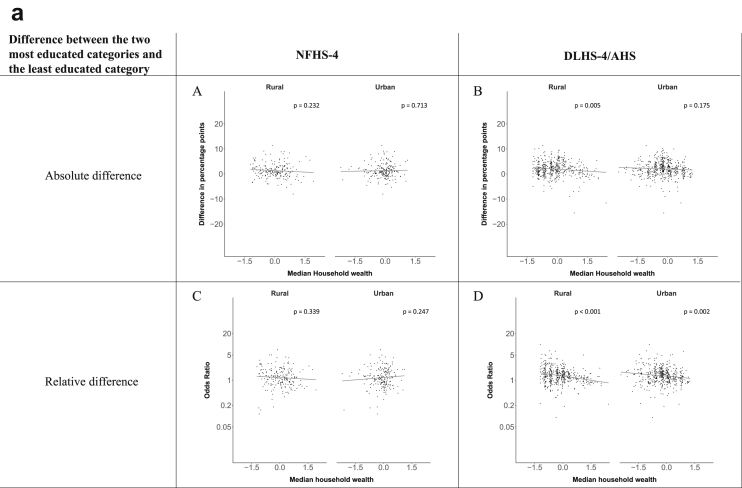
**Diabetes: association of district-level median household wealth with the difference between completing at least secondary school and less than primary school.** The points in the plot represent the regression coefficient from a linear probability model (for the absolute difference) and the Odds Ratio from a logistic regression (for the relative difference) comparing those participants who completed at least secondary school to those who did not complete primary school education in a district. These regressions regressed diabetes onto sex, age, and urban/rural residency separately for each district. The analysis included 200 districts in the NFHS-4 and 469 districts in the DLHS-4/AHS. The grey line through the scatterplots has been fitted using ordinary least squares regression (with each data point in the plot having the same weight). The p-value shows whether the slope of the grey line is significantly different from zero. The y-axis for the relative difference is on the logarithmic scale.

**Fig. 5b:**
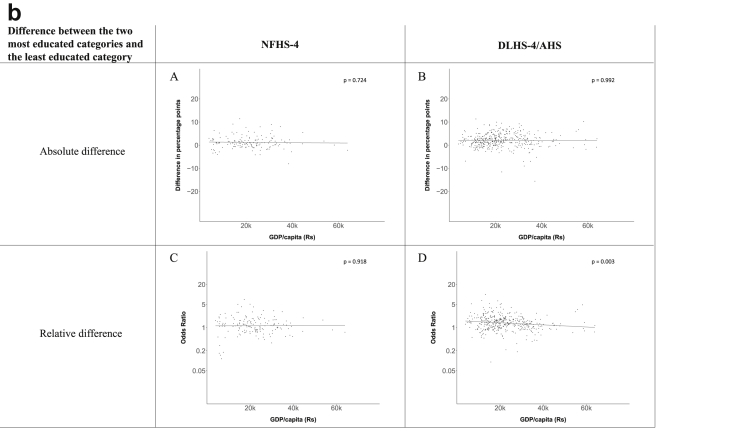
**Diabetes: association of a district's GDP/capita with the difference between completing at least secondary school and less than primary school.** The points in the plot represent the regression coefficient from a linear probability model (for the absolute difference) and the Odds Ratio from a logistic regression (for the relative difference) comparing those participants who completed at least secondary school to those who did not complete primary school education in a district. These regressions regressed diabetes onto sex, age, and urban/rural residency separately for each district. The analysis included 155 districts in the NFHS-4 and 393 districts in the DLHS-4/AHS. The grey line through the scatterplots has been fitted using ordinary least squares regression (with each data point in the plot having the same weight). The p-value shows whether the slope of the grey line is significantly different from zero. The y-axis for the relative difference is on the logarithmic scale.

**Fig. 5c:**
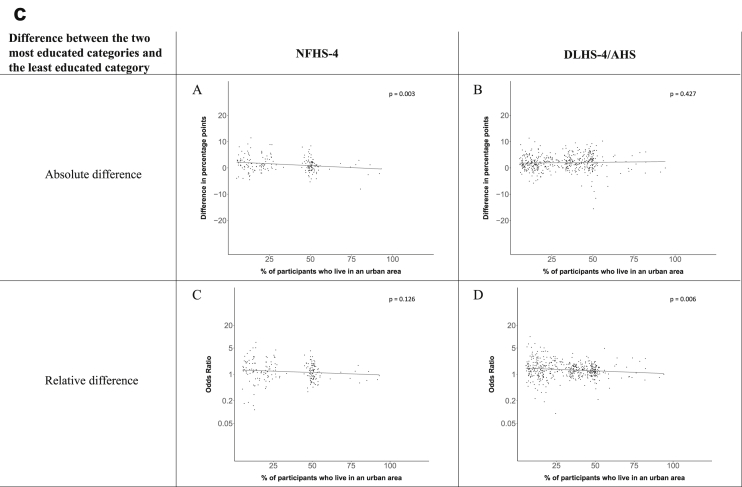
**Diabetes: association of a district's urban population with the difference between completing at least secondary school and less than primary school.** The points in the plot represent the regression coefficient from a linear probability model (for the absolute difference) and the Odds Ratio from a logistic regression (for the relative difference) comparing those participants who completed at least secondary school to those who did not complete primary school education in a district. These regressions regressed diabetes onto sex, age, and urban/rural residency separately for each district. The analysis included 200 districts in the NFHS-4 and 469 districts in the DLHS-4/AHS. The grey line through the scatterplots has been fitted using ordinary least squares regression (with each data point in the plot having the same weight). The p-value shows whether the slope of the grey line is significantly different from zero. The y-axis for the relative difference is on the logarithmic scale.

**Fig. 5d:**
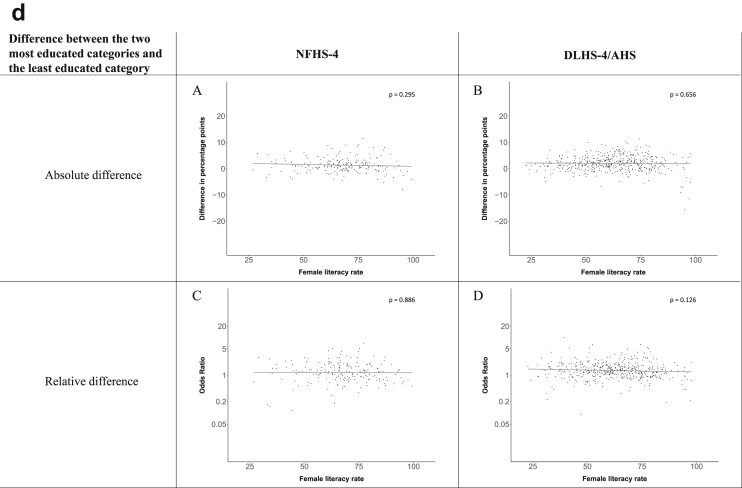
**Diabetes: association of district-level female literacy with the difference between completing at least secondary school and less than primary school.** The points in the plot represent the regression coefficient from a linear probability model (for the absolute difference) and the Odds Ratio from a logistic regression (for the relative difference) comparing those participants who completed at least secondary school to those who did not complete primary school education in a district. These regressions regressed diabetes onto sex, age, and urban/rural residency separately for each district. The analysis included 200 districts in the NFHS-4 and 469 districts in the DLHS-4/AHS. The grey line through the scatterplots has been fitted using ordinary least squares regression (with each data point in the plot having the same weight). The p-value shows whether the slope of the grey line is significantly different from zero. The y-axis for the relative difference is on the logarithmic scale.

**Fig. 6a:**
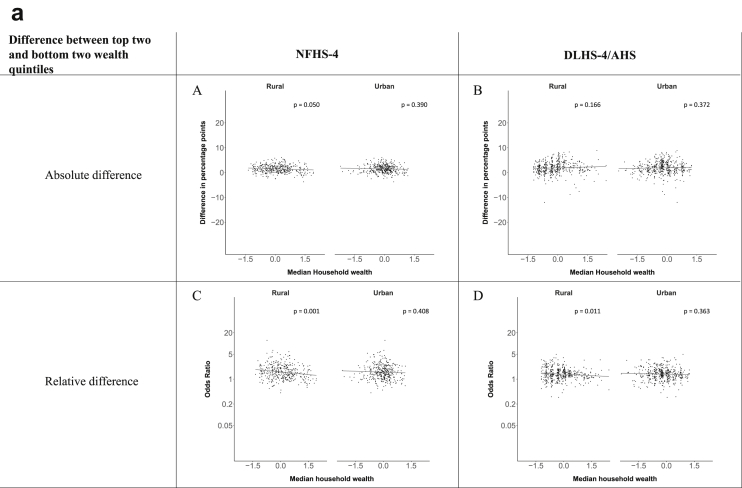
**Diabetes: association of district-level median household wealth with the difference between the top two and bottom two household wealth quintiles computed for each district.** The points in the plot represent the regression coefficient from a linear probability model (for the absolute difference) and the Odds Ratio from a logistic regression (for the relative difference) comparing the richest to the poorest household wealth quintile in a district. These regressions regressed diabetes onto sex, age, and urban/rural residency separately for each district. The analysis included 373 districts in the NFHS-4 and 477 districts in the DLHS-4/AHS. The grey line through the scatterplots has been fitted using ordinary least squares regression (with each data point in the plot having the same weight). The p-value shows whether the slope of the grey line is significantly different from zero. The y-axis for the relative difference is on the logarithmic scale.

**Fig. 6b:**
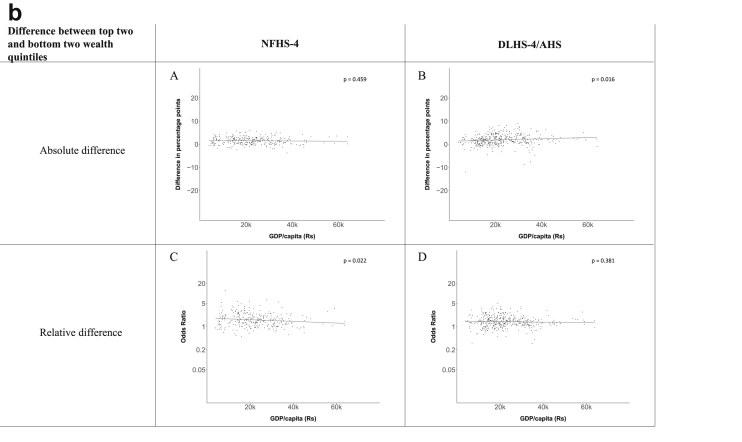
**Diabetes: association of a district's GDP/capita with the difference between the top two and bottom two household wealth quintiles computed for each district.** The points in the plot represent the regression coefficient from a linear probability model (for the absolute difference) and the Odds Ratio from a logistic regression (for the relative difference) comparing the richest to the poorest household wealth quintile in a district. These regressions regressed diabetes onto sex, age, and urban/rural residency separately for each district. The analysis included 282 districts in the NFHS-4 and 401 districts in the DLHS-4/AHS. The grey line through the scatterplots has been fitted using ordinary least squares regression (with each data point in the plot having the same weight). The p-value shows whether the slope of the grey line is significantly different from zero. The y-axis for the relative difference is on the logarithmic scale.

**Fig. 6c:**
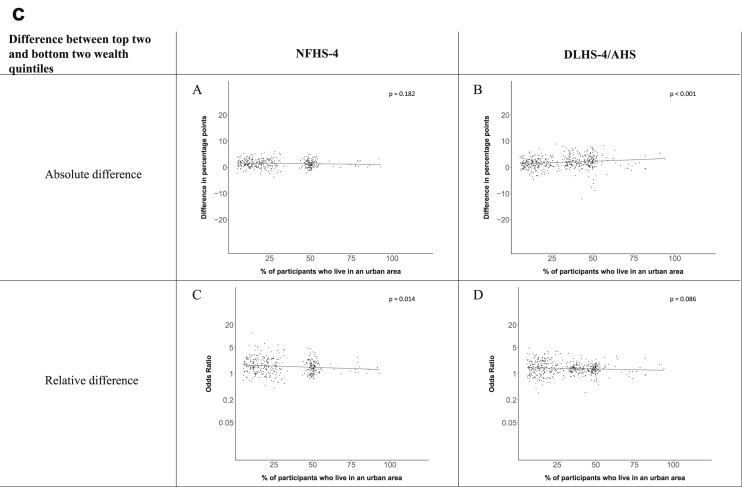
**Diabetes: association of a district's urban population with the difference between the top two and bottom two household wealth quintiles computed for each district.** The points in the plot represent the regression coefficient from a linear probability model (for the absolute difference) and the Odds Ratio from a logistic regression (for the relative difference) comparing the richest to the poorest household wealth quintile in a district. These regressions regressed diabetes onto sex, age, and urban/rural residency separately for each district. The analysis included 373 districts in the NFHS-4 and 477 districts in the DLHS-4/AHS. The grey line through the scatterplots has been fitted using ordinary least squares regression (with each data point in the plot having the same weight). The p-value shows whether the slope of the grey line is significantly different from zero. The y-axis for the relative difference is on the logarithmic scale.

**Fig. 6d:**
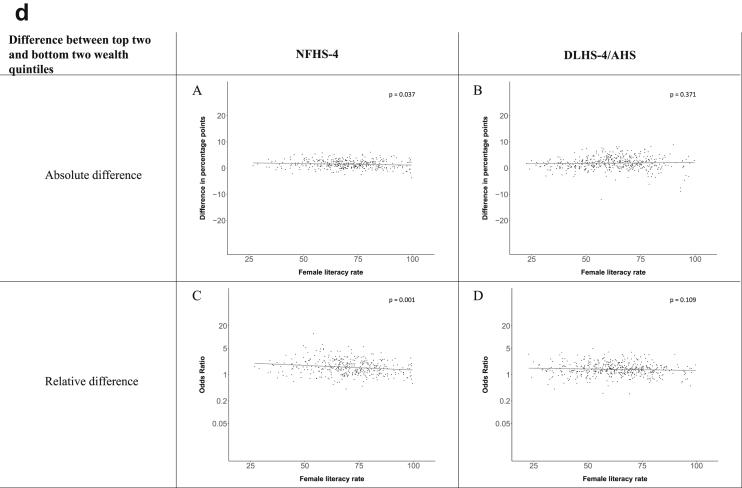
**Diabetes: association district-level female literacy with the difference between the top two and bottom two household wealth quintiles computed for each district.** The points in the plot represent the regression coefficient from a linear probability model (for the absolute difference) and the Odds Ratio from a logistic regression (for the relative difference) comparing the richest to the poorest household wealth quintile in a district. These regressions regressed diabetes onto sex, age, and urban/rural residency separately for each district. The analysis included 373 districts in the NFHS-4 and 477 districts in the DLHS-4/AHS. The grey line through the scatterplots has been fitted using ordinary least squares regression (with each data point in the plot having the same weight). The p-value shows whether the slope of the grey line is significantly different from zero. The y-axis for the relative difference is on the logarithmic scale.

**Fig. 7a:**
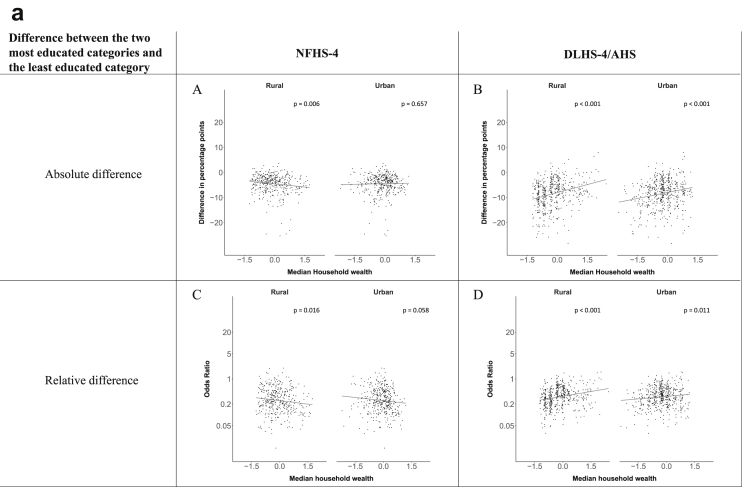
**Current smoking: association of district-level median household wealth with the difference between completing at least secondary school and less than primary school.** The points in the plot represent the regression coefficient from a linear probability model (for the absolute difference) and the Odds Ratio from a logistic regression (for the relative difference) comparing those participants who completed at least secondary school to those who did not complete primary school education in a district. These regressions regressed current smoking onto sex, age, and urban/rural residency separately for each district. The analysis included 390 districts in the NFHS-4 and 508 districts in the DLHS-4/AHS. The grey line through the scatterplots has been fitted using ordinary least squares regression (with each data point in the plot having the same weight). The p-value shows whether the slope of the grey line is significantly different from zero. The y-axis for the relative difference is on the logarithmic scale.

**Fig. 7b:**
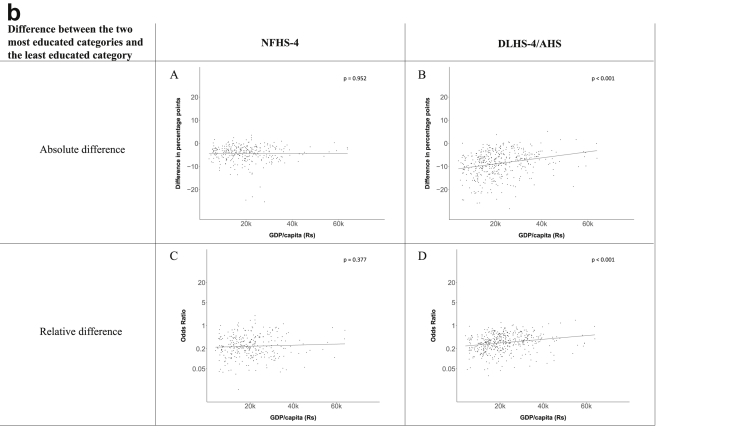
**Current smoking: association of a district's GDP/capita with the difference between completing at least secondary school and less than primary school.** The points in the plot represent the regression coefficient from a linear probability model (for the absolute difference) and the Odds Ratio from a logistic regression (for the relative difference) comparing those participants who completed at least secondary school to those who did not complete primary school education in a district. These regressions regressed current smoking onto sex, age, and urban/rural residency separately for each district. The analysis included 303 districts in the NFHS-4 and 429 districts in the DLHS-4/AHS. The grey line through the scatterplots has been fitted using ordinary least squares regression (with each data point in the plot having the same weight). The p-value shows whether the slope of the grey line is significantly different from zero. The y-axis for the relative difference is on the logarithmic scale.

**Fig. 7c:**
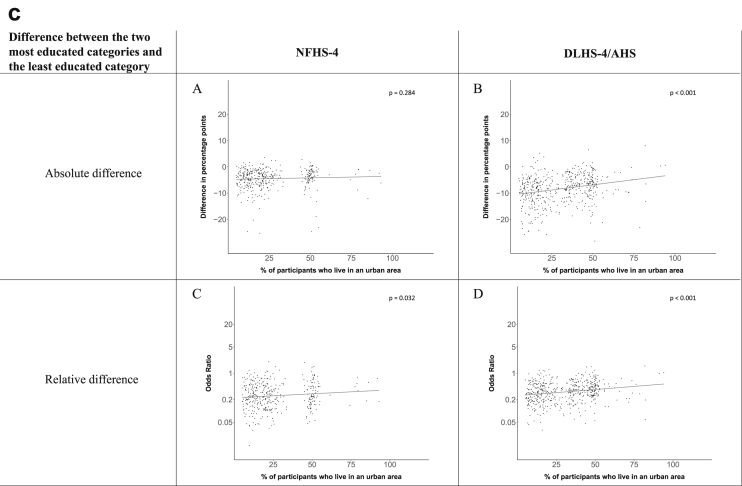
**Current smoking: association of a district's urban population with the difference between completing at least secondary school and less than primary school.** The points in the plot represent the regression coefficient from a linear probability model (for the absolute difference) and the Odds Ratio from a logistic regression (for the relative difference) comparing those participants who completed at least secondary school to those who did not complete primary school education in a district. These regressions regressed current smoking onto sex, age, and urban/rural residency separately for each district. The analysis included 390 districts in the NFHS-4 and 508 districts in the DLHS-4/AHS. The grey line through the scatterplots has been fitted using ordinary least squares regression (with each data point in the plot having the same weight). The p-value shows whether the slope of the grey line is significantly different from zero. The y-axis for the relative difference is on the logarithmic scale.

**Fig. 7d:**
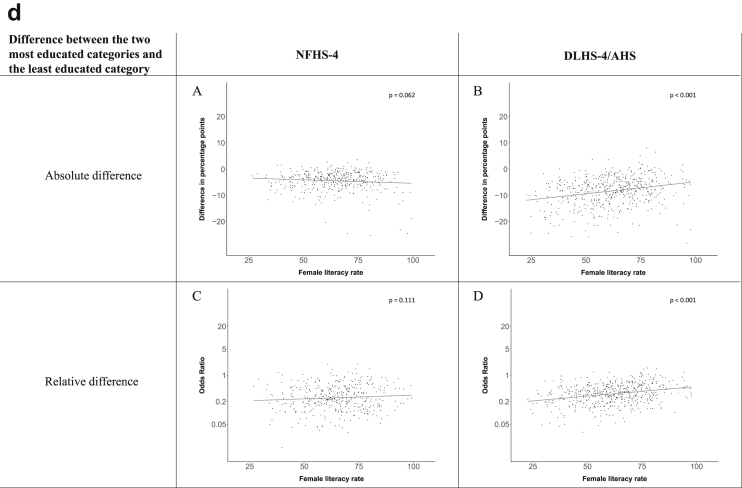
**Current smoking: association of district-level female literacy with the difference between completing at least secondary school and less than primary school.** The points in the plot represent the regression coefficient from a linear probability model (for the absolute difference) and the Odds Ratio from a logistic regression (for the relative difference) comparing those participants who completed at least secondary school to those who did not complete primary school education in a district. These regressions regressed current smoking onto sex, age, and urban/rural residency separately for each district. The analysis included 390 districts in the NFHS-4 and 508 districts in the DLHS-4/AHS. The grey line through the scatterplots has been fitted using ordinary least squares regression (with each data point in the plot having the same weight). The p-value shows whether the slope of the grey line is significantly different from zero. The y-axis for the relative difference is on the logarithmic scale.

**Fig. 8a:**
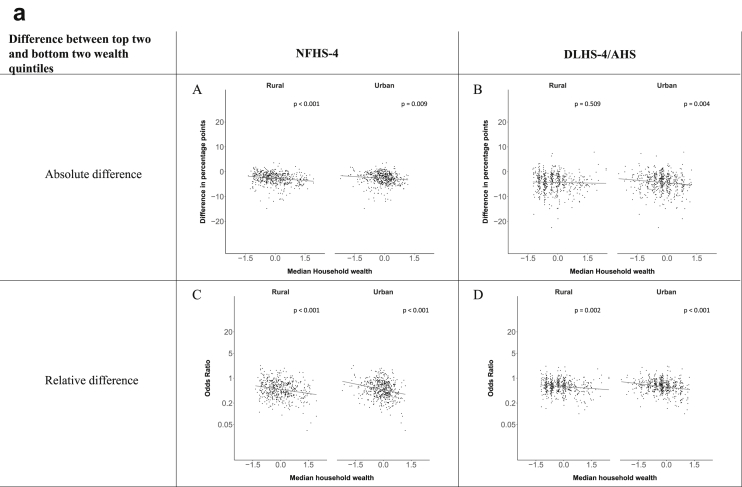
**Current smoking: association of district-level median household wealth with the difference between the top two and bottom two household wealth quintiles computed for each district.** The points in the plot represent the regression coefficient from a linear probability model (for the absolute difference) and the Odds Ratio from a logistic regression (for the relative difference) comparing the richest to the poorest household wealth quintile in a district. These regressions regressed current smoking (as a binary variable) onto sex, age, and urban/rural residency separately for each district. The analysis included 513 districts in the NFHS-4 and 514 districts in the DLHS-4/AHS. The grey line through the scatterplots has been fitted using ordinary least squares regression (with each data point in the plot having the same weight). The p-value shows the whether the slope of the grey line is significantly different from zero. The y-axis for the relative difference is on the logarithmic scale.

**Fig. 8b:**
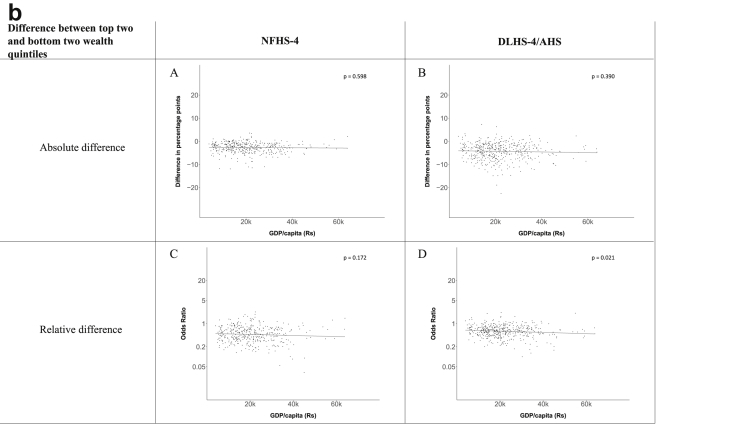
**Current smoking: association of a district's GDP/capita with the difference between the top two and bottom two household wealth quintiles computed for each district.** The points in the plot represent the regression coefficient from a linear probability model (for the absolute difference) and the Odds Ratio from a logistic regression (for the relative difference) comparing the richest to the poorest household wealth quintile in a district. These regressions regressed current smoking (as a binary variable) onto sex, age, and urban/rural residency separately for each district. The analysis included 387 districts in the NFHS-4 and 434 districts in the DLHS-4/AHS. The grey line through the scatterplots has been fitted using ordinary least squares regression (with each data point in the plot having the same weight). The p-value shows the whether the slope of the grey line is significantly different from zero. The y-axis for the relative difference is on the logarithmic scale.

**Fig. 8c:**
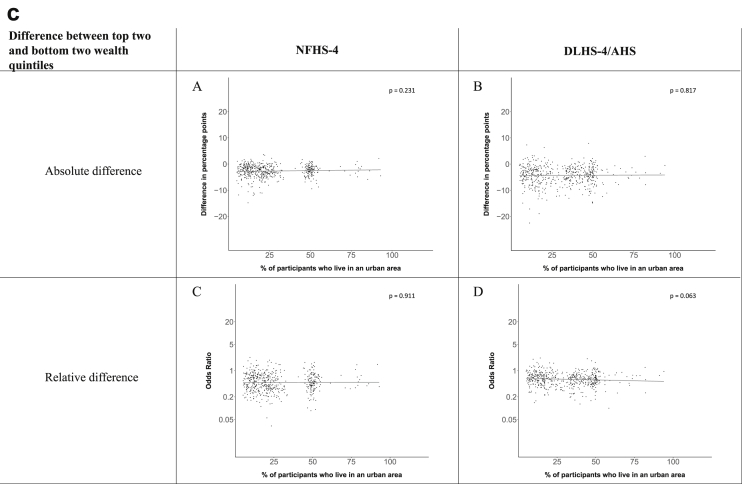
**Current smoking: association of a district's urban population with the difference between the top two and bottom two household wealth quintiles computed for each district.** The points in the plot represent the regression coefficient from a linear probability model (for the absolute difference) and the Odds Ratio from a logistic regression (for the relative difference) comparing the richest to the poorest household wealth quintile in a district. These regressions regressed current smoking (as a binary variable) onto sex, age, and urban/rural residency separately for each district. The analysis included 513 districts in the NFHS-4 and 514 districts in the DLHS-4/AHS. The grey line through the scatterplots has been fitted using ordinary least squares regression (with each data point in the plot having the same weight). The p-value shows the whether the slope of the grey line is significantly different from zero. The y-axis for the relative difference is on the logarithmic scale.

**Fig. 8d:**
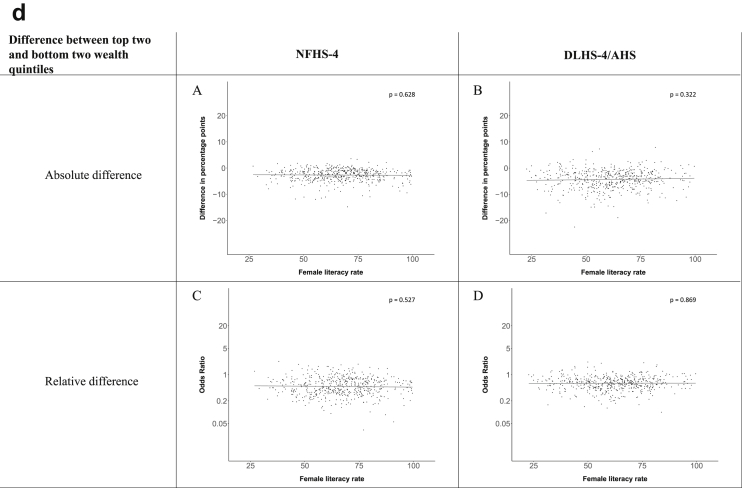
**Current smoking: association district-level female literacy with the difference between the top two and bottom two household wealth quintiles computed for each district.** The points in the plot represent the regression coefficient from a linear probability model (for the absolute difference) and the Odds Ratio from a logistic regression (for the relative difference) comparing the richest to the poorest household wealth quintile in a district. These regressions regressed current smoking (as a binary variable) onto sex, age, and urban/rural residency separately for each district. The analysis included 513 districts in the NFHS-4 and 514 districts in the DLHS-4/AHS. The grey line through the scatterplots has been fitted using ordinary least squares regression (with each data point in the plot having the same weight). The p-value shows the whether the slope of the grey line is significantly different from zero. The y-axis for the relative difference is on the logarithmic scale.

**Fig. 9a:**
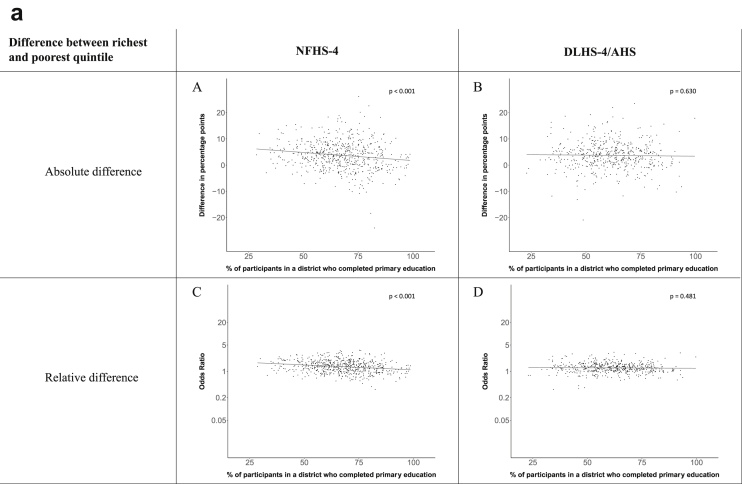
**Hypertension: association district-level primary school completion rate with the difference between richest and poorest household wealth quintile computed for each district.** The points in the plot represent the regression coefficient from a linear probability model (for the absolute difference) and the Odds Ratio from a logistic regression (for the relative difference) comparing the richest to the poorest household wealth quintile in a district. These regressions regressed hypertension onto sex, age, and urban/rural residency separately for each district. The analysis included 606 districts in the NFHS-4 and 517 districts in the DLHS-4/AHS. The grey line through the scatterplots has been fitted using ordinary least squares regression (with each data point in the plot having the same weight). The p-value shows whether the slope of the grey line is significantly different from zero. The y-axis for the relative difference is on the logarithmic scale.

**Fig. 9b:**
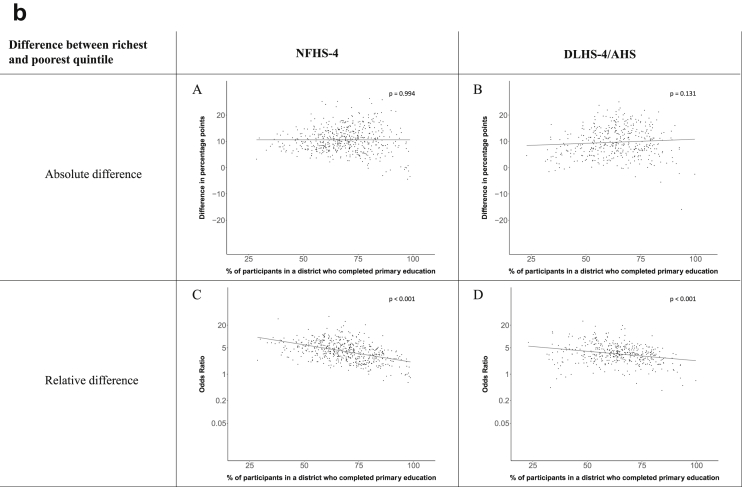
**Obesity: association district-level primary school completion rate with the difference between richest and poorest household wealth quintiles computed for each district.** The points in the plot represent the regression coefficient from a linear probability model (for the absolute difference) and the Odds Ratio from a logistic regression (for the relative difference) comparing the richest to the poorest household wealth quintile in a district. These regressions regressed obesity onto sex, age, and urban/rural residency separately for each district. The analysis included 528 districts in the NFHS-4 and 413 districts in the DLHS-4/AHS. The grey line through the scatterplots has been fitted using ordinary least squares regression (with each data point in the plot having the same weight). The p-value shows whether the slope of the grey line is significantly different from zero. The y-axis for the relative difference is on the logarithmic scale.

**Fig. 9c:**
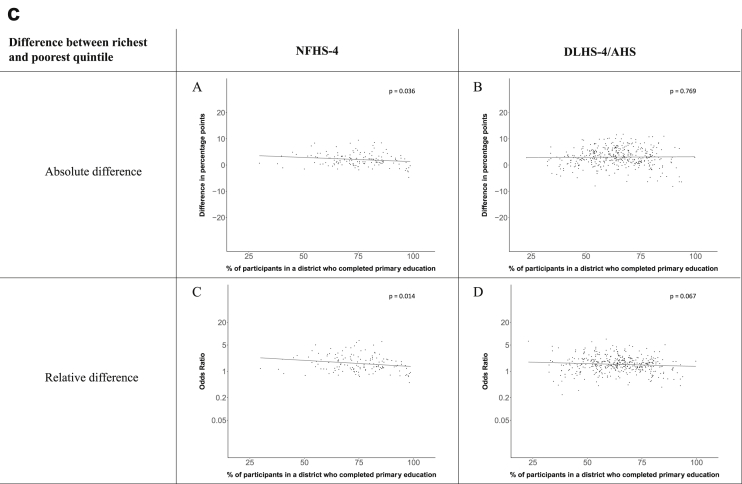
**Diabetes: association district-level primary school completion rate with the difference between richest and poorest household wealth quintiles computed for each district.** The points in the plot represent the regression coefficient from a linear probability model (for the absolute difference) and the Odds Ratio from a logistic regression (for the relative difference) comparing the richest to the poorest household wealth quintile in a district. These regressions regressed diabetes onto sex, age, and urban/rural residency separately for each district. The analysis included 142 districts in the NFHS-4 and 408 districts in the DLHS-4/AHS. The grey line through the scatterplots has been fitted using ordinary least squares regression (with each data point in the plot having the same weight). The p-value shows whether the slope of the grey line is significantly different from zero. The y-axis for the relative difference is on the logarithmic scale.

**Fig. 9d:**
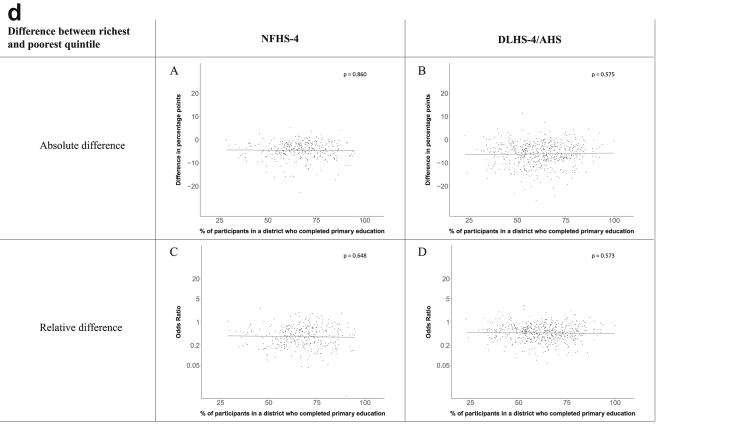
**Current smoking: association district-level primary school completion rate with the difference between richest and poorest household wealth quintile computed for each district.** The points in the plot represent the regression coefficient from a linear probability model (for the absolute difference) and the Odds Ratio from a logistic regression (for the relative difference) comparing the richest to the poorest household wealth quintile in a district. These regressions regressed current smoking (as a binary variable) onto sex, age, and urban/rural residency separately for each district. The analysis included 314 districts in the NFHS-4 and 503 districts in the DLHS-4/AHS. The grey line through the scatterplots has been fitted using ordinary least squares regression (with each data point in the plot having the same weight). The p-value shows the whether the slope of the grey line is significantly different from zero. The y-axis for the relative difference is on the logarithmic scale.

**Fig. 10a:**
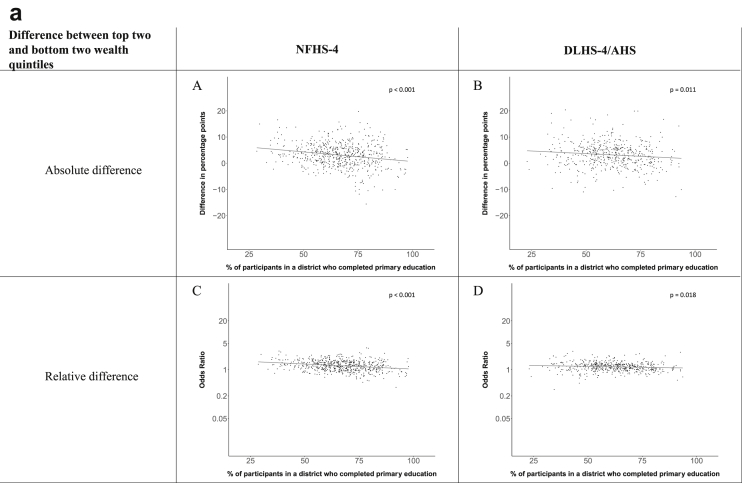
**Hypertension: association of district-level primary school completion with the difference between the top two and bottom two household wealth quintiles computed nationally.** The points in the plot represent the regression coefficient from a linear probability model (for the absolute difference) and the Odds Ratio from a logistic regression (for the relative difference) comparing the top two to the bottom two household wealth quintiles in a district. These regressions regressed hypertension onto sex, age, and urban/rural residency separately for each district. The analysis included 591 districts in the NFHS-4 and 501 districts in the DLHS-4/AHS. The grey line through the scatterplots has been fitted using ordinary least squares regression (with each data point in the plot having the same weight). The p-value shows whether the slope of the grey line is significantly different from zero. The y-axis for the relative difference is on the logarithmic scale.

**Fig. 10b:**
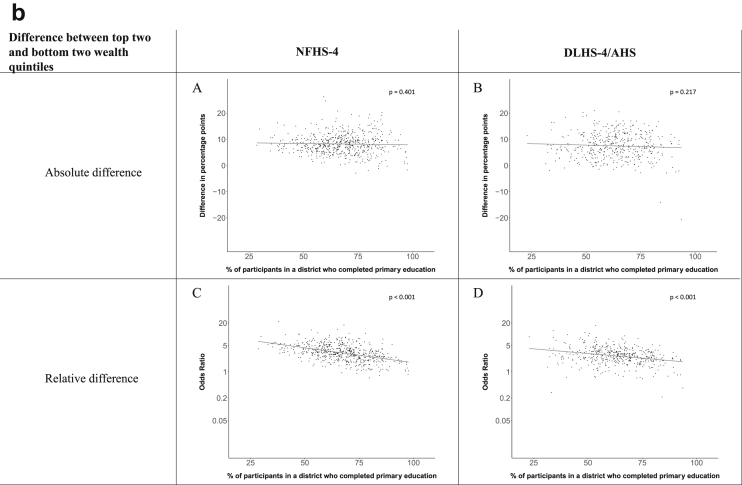
**Obesity: association of district-level primary school completion with the difference between the top two and bottom two household wealth quintiles computed nationally.** The points in the plot represent the regression coefficient from a linear probability model (for the absolute difference) and the Odds Ratio from a logistic regression (for the relative difference) comparing the top two to the bottom two household wealth quintiles in a district. These regressions regressed obesity onto sex, age, and urban/rural residency separately for each district. The analysis included 573 districts in the NFHS-4 and 448 districts in the DLHS-4/AHS. The grey line through the scatterplots has been fitted using ordinary least squares regression (with each data point in the plot having the same weight). The p-value shows whether the slope of the grey line is significantly different from zero. The y-axis for the relative difference is on the logarithmic scale.

**Fig. 10c:**
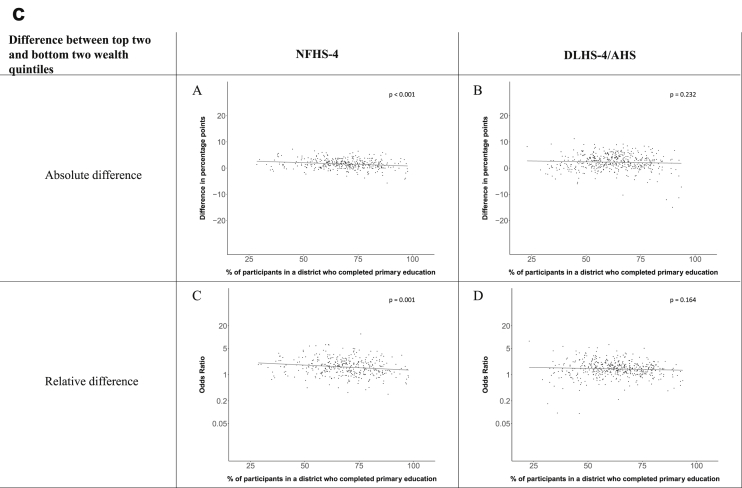
**Diabetes: association of district-level primary school completion with the difference between the top two and bottom two household wealth quintiles computed nationally.** The points in the plot represent the regression coefficient from a linear probability model (for the absolute difference) and the Odds Ratio from a logistic regression (for the relative difference) comparing the top two to the bottom two household wealth quintiles in a district. These regressions regressed diabetes onto sex, age, and urban/rural residency separately for each district. The analysis included 368 districts in the NFHS-4 and 466 districts in the DLHS-4/AHS. The grey line through the scatterplots has been fitted using ordinary least squares regression (with each data point in the plot having the same weight). The p-value shows whether the slope of the grey line is significantly different from zero. The y-axis for the relative difference is on the logarithmic scale.

**Fig. 10d:**
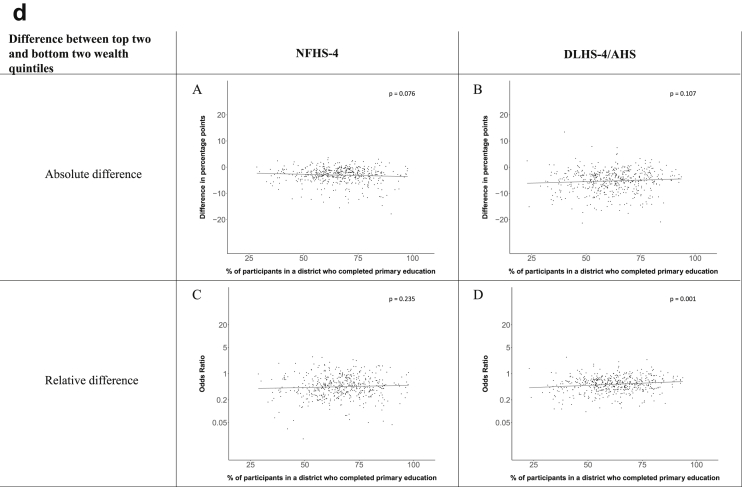
**Current smoking: association of district-level primary school completion with the difference between the top two and bottom two household wealth quintile computed nationally.** The points in the plot represent the regression coefficient from a linear probability model (for the absolute difference) and the Odds Ratio from a logistic regression (for the relative difference) comparing the top two to the bottom two household wealth quintiles in a district. These regressions regressed current smoking onto sex, age, and urban/rural residency separately for each district. The analysis included 491 districts in the NFHS-4 and 499 districts in the DLHS-4/AHS. The grey line through the scatterplots has been fitted using ordinary least squares regression (with each data point in the plot having the same weight). The p-value shows whether the slope of the grey line is significantly different from zero. The y-axis for the relative difference is on the logarithmic scale.

**Table 3:** Number of districts included in district-level regressions.^a^

	Two highest vs lowest education categories		Top two vs bottom two household wealth quintile	
	DLHS-4/AHS	NFHS-4	DLHS-4/AHS	NFHS-4
Hypertension	516 (516)	595 (595)	517 (517)	608 (608)
Obesity	443 (516)	531 (595)	461 (517)	589 (608)
Diabetes	469 (516)	200 (595)	477 (517)	373 (608)
Smoking	508 (516)	390 (595)	514 (517)	513 (608)

a Numbers in brackets are the numbers of districts remaining after excluding districts with urban population <5% or >95% and fewer than 50 participants in low or high SES category. Numbers without brackets are the final numbers for analysis (after excluding districts with fewer than 20 cases jointly in the low and high SES category for each risk factor).

Multilevel linear regressions for the interaction between district-level socio-economic development and participants’ educational attainment or household wealth, computed for each district and nationally, are shown for hypertension (Table 4, Table 5), obesity (Table 6, Table 7), diabetes (Table 8, Table 9) and currently smoking (Table 10, Table 11). As before, district-level indicators of socio-economic development were median household wealth, GDP per capita, percentage of participants living in an urban area, and female literacy rate. In addition, multilevel linear regressions with all our available indicators for district-level development (including primary school completion rate) were fitted for the following outcome variables: high blood pressure (Table 12, Table 13) and high blood glucose (Table 14, Table 15) in the NFHS-4 dataset, diabetes assuming that AHS participants have not fasted (Table 16, Table 17), and currently smoking separately for male (Table 18, Table 19) and female (Table 20, Table 21) survey participants.

**Table 4:** Results from multilevel linear regressions for the interaction between district-level socio-economic development and participants’ education and household wealth: Hypertension.^a^^,^^b^

		NFHS-4		DLHS-4/AHS	
		Absolute difference (% points)	P	Absolute difference (% points)	P
**Interaction of the district-level indicators with educational attainment**^c^					
Median household wealth	< primary	0.00 (Ref.)		0.00 (Ref.)	
	Primary completed	−0.15 [−0.86, 0.57]	0.690	−0.38 [−0.85, 0.10]	0.117
	Some secondary	−1.63 [−2.06, −1.20]	<0.001	−0.73 [−1.10, −0.37]	<0.001
	Secondary completed	−2.29 [−2.93, −1.66]	<0.001	−2.35 [−2.86, −1.84]	<0.001
	> secondary	−4.19 [−4.80, −3.58]	<0.001	−2.48 [−3.02, −1.95]	<0.001
GDP/capita	< primary	0.00 (Ref.)		0.00 (Ref.)	
	Primary completed	−1.06 [−1.89, −0.24]	0.011	−1.14 [−1.65, −0.62]	<0.001
	Some secondary	−2.25 [−2.74, −1.77]	<0.001	−1.67 [−2.06, −1.27]	<0.001
	Secondary completed	−2.85 [−3.55, −2.15]	<0.001	−2.64 [−3.20, −2.08]	<0.001
	> secondary	−4.14 [−4.79, −3.48]	<0.001	−3.52 [−4.09, −2.94]	<0.001
% of participants who live in an urban area	< primary	0.00 (Ref.)		0.00 (Ref.)	
	Primary completed	0.18 [−0.58, 0.94]	0.638	−0.56 [−1.04, −0.07]	0.025
	Some secondary	−0.55 [−0.99, −0.11]	0.013	−1.05 [−1.42, −0.69]	<0.001
	Secondary completed	−0.96 [−1.59, −0.32]	0.003	−2.25 [−2.76, −1.74]	<0.001
	> secondary	−1.94 [−2.52, −1.36]	<0.001	−2.74 [−3.25, −2.23]	<0.001
Female literacy rate	< primary	0.00 (Ref.)		0.00 (Ref.)	
	Primary completed	−1.21 [−1.97, −0.46]	0.002	−1.61 [−2.09, −1.13]	<0.001
	Some secondary	−2.13 [−2.56, −1.69]	<0.001	−1.97 [−2.35, −1.60]	<0.001
	Secondary completed	−2.70 [−3.37, −2.02]	<0.001	−2.88 [−3.43, −2.33]	<0.001
	> secondary	−3.50 [−4.14, −2.86]	<0.001	−2.81 [−3.38, −2.25]	<0.001
**Interaction of the district-level indicators with household wealth quintile computed in each district**^d^					
Median household wealth	1 (poorest)	0.00 (Ref.)		0.00 (Ref.)	
	2	0.67 [0.14, 1.19]	0.013	1.24 [0.79, 1.69]	<0.001
	3	0.51 [−0.01, 1.04]	0.056	1.51 [1.06, 1.96]	<0.001
	4	0.06 [−0.47, 0.58]	0.825	1.12 [0.67, 1.57]	<0.001
	5 (richest)	−1.35 [−1.87, −0.82]	<0.001	0.47 [0.02, 0.92]	0.042
GDP/capita	1 (poorest)	0.00 (Ref.)		0.00 (Ref.)	
	2	0.34 [−0.26, 0.93]	0.264	0.41 [−0.09, 0.91]	0.105
	3	−0.16 [−0.75, 0.44]	0.605	0.09 [−0.41, 0.58]	0.729
	4	−0.98 [−1.57, −0.38]	0.001	0.20 [−0.30, 0.69]	0.436
	5 (richest)	−1.68 [−2.28, −1.09]	<0.001	−0.29 [−0.78, 0.21]	0.258
% of participants who live in an urban area	1 (poorest)	0.00 (Ref.)		0.00 (Ref.)	
	2	0.18 [−0.35, 0.71]	0.503	0.17 [−0.28, 0.62]	0.453
	3	0.23 [−0.30, 0.76]	0.392	0.43 [−0.02, 0.88]	0.060
	4	−0.40 [−0.93, 0.13]	0.140	0.18 [−0.27, 0.63]	0.425
	5 (richest)	−1.41 [−1.94, −0.87]	<0.001	−0.91 [−1.36, −0.45]	<0.001
Female literacy rate	1 (poorest)	0.00 (Ref.)		0.00 (Ref.)	
	2	0.54 [0.02, 1.07]	0.042	0.81 [0.35, 1.26]	<0.001
	3	0.11 [−0.41, 0.64]	0.672	0.36 [−0.09, 0.82]	0.118
	4	−0.33 [−0.86, 0.19]	0.217	0.14 [−0.31, 0.60]	0.538
	5 (richest)	−1.51 [−2.04, −0.99]	<0.001	−0.62 [−1.08, −0.17]	0.007
**Interaction of district-level development with household wealth quintile computed nationally**^d^					
Median household wealth	1 (poorest)	0.00 (Ref.)		0.00 (Ref.)	
	2	0.02 [−0.65, 0.69]	0.954	0.00 [−0.61, 0.61]	0.994
	3	−0.20 [−0.89, 0.48]	0.560	−0.97 [−1.61, −0.32]	0.003
	4	−1.03 [−1.72, −0.33]	0.004	−1.00 [−1.64, −0.36]	0.002
	5 (richest)	−1.10 [−1.81, −0.40]	0.002	−0.83 [−1.48, −0.18]	0.012
GDP/capita	1 (poorest)	0.00 (Ref.)		0.00 (Ref.)	
	2	0.25 [−0.45, 0.95]	0.487	0.10 [−0.48, 0.68]	0.728
	3	−0.34 [−1.04, 0.37]	0.351	−0.53 [−1.14, 0.08]	0.089
	4	−1.31 [−2.01, −0.60]	<0.001	−1.30 [−1.91, −0.69]	<0.001
	5 (richest)	−0.95 [−1.66, −0.23]	0.010	−1.56 [−2.20, −0.91]	<0.001
% of participants who live in an urban area	1 (poorest)	0.00 (Ref.)		0.00 (Ref.)	
	2	−0.06 [−0.65, 0.53]	0.841	−0.34 [−0.83, 0.15]	0.170
	3	−0.21 [−0.81, 0.38]	0.485	−0.80 [−1.31, −0.29]	0.002
	4	−0.88 [−1.49, −0.28]	0.004	−1.23 [−1.76, −0.71]	<0.001
	5 (richest)	−1.36 [−1.98, −0.73]	<0.001	−2.73 [−3.29, −2.17]	<0.001
Female literacy rate	1 (poorest)	0.00 (Ref.)		0.00 (Ref.)	
	2	0.05 [−0.52, 0.61]	0.871	−0.29 [−0.75, 0.17]	0.213
	3	−0.34 [−0.93, 0.24]	0.251	−0.37 [−0.87, 0.14]	0.154
	4	−1.22 [−1.83, −0.61]	<0.001	−1.54 [−2.08, −1.01]	<0.001
	5 (richest)	−1.29 [−1.93, −0.66]	<0.001	−1.87 [−2.45, −1.29]	<0.001

**Abbreviations:** Ref. = Reference category; NFHS-4 = National Family Health Survey 4; DLHS-4 = District-Level Household Survey 4; AHS = Annual Health Survey.

a All multilevel linear regression models i) had hypertension as outcome variable; ii) contained a random intercept for district; iii) had five-year age group, sex, urban/rural residency as level 1 (the individual level) independent variables; and iv) district-level median household wealth, Gross Domestic Product (GDP) per capita, the percentage of participants in a district living in an urban area, and district female literacy rate as level 2 (the district level) independent variable.

b The numbers in square brackets are 95% confidence intervals.

c These models included educational attainment as level 1 independent variable and an interaction term between educational attainment and the district-level indicator.

d These models included household wealth quintile as level 1 independent variable and an interaction term between household wealth quintile and the district-level indicator.

**Table 5:** Results from multilevel linear regressions for individual-level variables: Hypertension.^a^^,^^b^

	NFHS-4		DLHS-4/AHS	
	Absolute difference (% points)	P	Absolute difference (% points)	P
**Interaction with educational attainment**^c^				
**Age group**				
15–19 years	0.00 (Ref.)		0.00 (Ref.)	
20–24 years	4.82 [4.52, 5.12]	<0.001	2.14 [1.76, 2.52]	<0.001
25–29 years	9.23 [8.93, 9.52]	<0.001	5.17 [4.80, 5.55]	<0.001
30–34 years	14.01 [13.70, 14.32]	<0.001	9.02 [8.64, 9.41]	<0.001
35–39 years	18.82 [18.51, 19.14]	<0.001	13.09 [12.70, 13.47]	<0.001
40–44 years	24.01 [23.68, 24.34]	<0.001	17.52 [17.13, 17.91]	<0.001
45–49 years	29.54 [29.20, 29.89]	<0.001	21.66 [21.26, 22.06]	<0.001
50–54 years	31.31 [30.46, 32.16]	<0.001	26.09 [25.68, 26.50]	<0.001
55–50 years	–	–	29.54 [29.11, 29.97]	<0.001
60–64 years	–	–	33.45 [33.00, 33.89]	<0.001
>65 years	–	–	38.06 [37.65, 38.46]	<0.001
**Educational attainment**				
< primary	0.00 (Ref.)		0.00 (Ref.)	
Primary completed	1.96 [1.61, 2.32]	<0.001	1.57 [1.34, 1.81]	<0.001
Some secondary	2.37 [2.14, 2.59]	<0.001	2.35 [2.15, 2.54]	<0.001
Secondary completed	2.48 [2.14, 2.82]	<0.001	2.23 [1.95, 2.51]	<0.001
> secondary	1.67 [1.35, 1.99]	<0.001	2.60 [2.32, 2.87]	<0.001
**Urban area**	2.32 [2.11, 2.53]	<0.001	3.15 [3.98, 3.32]	<0.001
**Female**	−1.90 [−2.15, −1.65]	<0.001	−3.71 [−3.85, −3.56]	<0.001
**Interaction with household wealth quintile computed in each district**^d^				
**Age group**				
15–19 years	0.00 (Ref.)		0.00 (Ref.)	
20–24 years	4.31 [4.02, 4.60]	<0.001	1.98 [1.59, 2.36]	<0.001
25–29 years	8.54 [8.25, 8.83]	<0.001	4.83 [4.44, 5.21]	<0.001
30–34 years	13.21 [12.92, 13.51]	<0.001	8.59 [8.20, 8.97]	<0.001
35–39 years	17.86 [17.55, 18.16]	<0.001	12.54 [12.15, 12.93]	<0.001
40–44 years	22.82 [22.51, 23.14]	<0.001	16.82 [16.43, 17.21]	<0.001
45–49 years	28.12 [27.80, 28.44]	<0.001	20.76 [20.36, 21.16]	<0.001
50–54 years	29.97 [29.13, 30.81]	<0.001	25.10 [24.69, 25.52]	<0.001
55–50 years	–	–	28.44 [28.01, 28.87]	<0.001
60–64 years	–	–	32.28 [31.84, 32.72]	<0.001
>65 years	–	–	36.79 [36.40, 37.19]	<0.001
**Household wealth quintile**				
1 (poorest)	0.00 (Ref.)		0.00 (Ref.)	
2	0.92 [0.66, 1.18]	<0.001	0.48 [0.26, 0.71]	<0.001
3	1.73 [1.47, 1.99]	<0.001	1.33 [1.10, 1.55]	<0.001
4	2.54 [2.28, 2.81]	<0.001	1.97 [1.75, 2.20]	<0.001
5 (richest)	3.66 [3.40, 3.92]	<0.001	3.55 [3.32, 3.77]	<0.001
**Urban area**	2.62 [2.42, 2.82]	<0.001	3.66 [3.49, 3.82]	<0.001
**Female**	−2.22 [–2.47, −1.97]	<0.001	−4.20 [–4.34, −4.06]	<0.001
**Interaction with household wealth quintile computed nationally**^d^				
**Age group**				
15–19 years	0.00 (Ref.)		0.00 (Ref.)	
20–24 years	4.29 [4.00, 4.58]	<0.001	1.99 [1.60, 2.37]	<0.001
25–29 years	8.52 [8.23, 8.81]	<0.001	4.84 [4.45, 5.22]	<0.001
30–34 years	13.19 [12.89, 13.49]	<0.001	8.59 [8.21, 8.98]	<0.001
35–39 years	17.83 [17.53, 18.13]	<0.001	12.54 [12.15, 12.93]	<0.001
40–44 years	22.80 [22.49, 23.11]	<0.001	16.82 [16.43, 17.21]	<0.001
45–49 years	28.09 [27.77, 28.41]	<0.001	20.75 [20.35, 21.16]	<0.001
50–54 years	29.93 [29.09, 30.77]	<0.001	25.10 [24.69, 25.51]	<0.001
55–50 years	–	–	28.44 [28.00, 28.87]	<0.001
60–64 years	–	–	32.29 [31.84, 32.73]	<0.001
>65 years	–	–	36.81 [36.41, 37.20]	<0.001
**Household wealth quintile**				
1 (poorest)	0.00 (Ref.)		0.00 (Ref.)	
2	1.38 [1.10, 1.65]	<0.001	0.72 [0.49, 0.96]	<0.001
3	2.34 [2.06, 2.62]	<0.001	1.69 [1.45, 1.94]	<0.001
4	3.20 [2.92, 3.49]	<0.001	2.64 [2.39, 2.89]	<0.001
5 (richest)	4.80 [4.50, 5.11]	<0.001	4.52 [4.25, 4.79]	<0.001
**Urban area**	3.15 [2.95, 3.36]	<0.001	4.08 [3.91, 4.25]	<0.001
**Female**	−2.23 [–2.47, −1.98]	<0.001	−4.21 [–4.35, −4.06]	<0.001

**Abbreviations:** Ref. = Reference category; NFHS-4 = National Family Health Survey 4; DLHS-4 = District-Level Household Survey 4; AHS = Annual Health Survey.

a All multilevel linear regression models i) had hypertension as outcome variable; ii) contained a random intercept for district; iii) had five-year age group, sex, urban/rural residency as level 1 (the individual level) independent variables.

b The numbers in square brackets are 95% confidence intervals.

c These models included educational attainment as level 1 independent variable.

d These models included household wealth quintile as level 1 independent variable.

**Table 6:** Results from multilevel linear regressions for the interaction between district-level socio-economic development and participants’ education and household wealth: Obesity.^a^^,^^b^

		NFHS-4		DLHS-4/AHS	
		Absolute difference (% points)	P	Absolute difference (% points)	P
**Interaction of the district-level indicators with educational attainment**^c^					
Median household wealth	< primary	0.00 (Ref.)		0.00 (Ref.)	
	Primary completed	−0.39 [−0.92, 0.15]	0.156	0.31 [0.00, 0.62]	0.049
	Some secondary	−2.03 [−2.35, −1.71]	<0.001	−0.12 [−0.36, 0.11]	0.306
	Secondary completed	−3.38 [−3.85, −2.91]	<0.001	−1.77 [−2.10, −1.43]	<0.001
	> secondary	−5.30 [−5.75, −4.84]	<0.001	−1.66 [−2.01, −1.31]	<0.001
GDP/capita	< primary	0.00 (Ref.)		0.00 (Ref.)	
	Primary completed	1.19 [0.57, 1.80]	<0.001	0.27 [−0.07, 0.61]	0.115
	Some secondary	−0.99 [−1.35, −0.63]	<0.001	0.41 [0.16, 0.67]	0.002
	Secondary completed	−2.26 [−2.78, −1.74]	<0.001	−0.41 [−0.77, −0.05]	0.026
	> secondary	−3.04 [−3.53, −2.55]	<0.001	0.10 [−0.28, 0.47]	0.618
% of participants who live in an urban area	< primary	0.00 (Ref.)		0.00 (Ref.)	
	Primary completed	0.09 [−0.48, 0.66]	0.754	0.74 [0.42, 1.06]	<0.001
	Some secondary	−1.30 [−1.63, −0.97]	<0.001	0.56 [0.32, 0.81]	<0.001
	Secondary completed	−2.30 [−2.77, −1.83]	<0.001	0.03 [−0.30, 0.37]	0.839
	> secondary	−3.14 [−3.57, −2.71]	<0.001	−0.53 [−0.87, −0.20]	0.002
Female literacy rate	< primary	0.00 (Ref.)		0.00 (Ref.)	
	Primary completed	−0.06 [−0.63, 0.50]	0.829	−0.40 [−0.72, −0.09]	0.012
	Some secondary	−1.25 [−1.57, −0.92]	<0.001	−0.32 [−0.56, −0.07]	0.011
	Secondary completed	−2.48 [−2.98, −1.97]	<0.001	0.14 [−0.50, 0.23]	0.426
	> secondary	−3.57 [−4.04, −3.09]	<0.001	−0.75 [−1.12, −0.38]	<0.001
**Interaction of the district-level indicators with household wealth quintile computed in each district**^d^					
Median household wealth	1 (poorest)	0.00 (Ref.)		0.00 (Ref.)	
	2	1.38 [0.99, 1.78]	<0.001	1.57 [1.27, 1.86]	<0.001
	3	2.36 [1.96, 2.75]	<0.001	3.28 [2.98, 3.57]	<0.001
	4	2.85 [2.46, 3.24]	<0.001	4.41 [4.11, 4.71]	<0.001
	5 (richest)	1.37 [0.98, 1.76]	<0.001	4.95 [4.65, 5.25]	<0.001
GDP/capita	1 (poorest)	0.00 (Ref.)		0.00 (Ref.)	
	2	0.97 [0.53, 1.42]	<0.001	1.41 [1.09, 1.74]	<0.001
	3	1.89 [1.45, 2.33]	<0.001	2.75 [2.42, 3.07]	<0.001
	4	2.20 [1.75, 2.64]	<0.001	3.99 [3.66, 4.31]	<0.001
	5 (richest)	1.15 [0.71, 1.60]	<0.001	4.94 [4.62, 5.27]	<0.001
% of participants who live in an urban area	1 (poorest)	0.00 (Ref.)		0.00 (Ref.)	
	2	1.33 [0.94, 1.73]	<0.001	1.41 [1.11, 1.70]	<0.001
	3	2.15 [1.75, 2.54]	<0.001	2.87 [2.57, 3.17]	<0.001
	4	2.50 [2.11, 2.90]	<0.001	3.83 [3.53, 4.13]	<0.001
	5 (richest)	1.32 [0.93, 1.72]	<0.001	4.47 [4.17, 4.77]	<0.001
Female literacy rate	1 (poorest)	0.00 (Ref.)		0.00 (Ref.)	
	2	1.14 [0.75, 1.53]	<0.001	0.76 [0.46, 1.06]	<0.001
	3	1.63 [1.24, 2.02]	<0.001	1.41 [1.11, 1.71]	<0.001
	4	1.95 [1.56, 2.35]	<0.001	1.79 [1.49, 2.09]	<0.001
	5 (richest)	0.81 [0.42, 1.21]	<0.001	2.18 [1.88, 2.48]	<0.001
**Interaction of the district-level indicators with household wealth quintile computed nationally**^d^					
Median household wealth	1 (poorest)	0.00 (Ref.)		0.00 (Ref.)	
	2	1.38 [0.89, 1.88]	<0.001	1.27 [0.87, 1.67]	<0.001
	3	2.00 [1.49, 2.51]	<0.001	1.88 [1.46, 2.31]	<0.001
	4	1.41 [0.90, 1.93]	<0.001	2.41 [1.99, 2.83]	<0.001
	5 (richest)	1.57 [1.05, 2.09]	<0.001	3.28 [2.85, 3.71]	<0.001
GDP/capita	1 (poorest)	0.00 (Ref.)		0.00 (Ref.)	
	2	1.33 [0.81, 1.85]	<0.001	1.35 [0.97, 1.73]	<0.001
	3	1.85 [1.33, 2.37]	<0.001	2.27 [1.87, 2.67]	<0.001
	4	1.42 [0.90, 1.94]	<0.001	2.53 [2.13, 2.93]	<0.001
	5 (richest)	1.75 [1.22, 2.28]	<0.001	3.33 [2.91, 3.75]	<0.001
% of participants who live in an urban area	1 (poorest)	0.00 (Ref.)		0.00 (Ref.)	
	2	1.54 [1.09, 1.98]	<0.001	1.07 [0.75, 1.40]	<0.001
	3	2.24 [1.80, 2.68]	<0.001	1.84 [1.51, 2.18]	<0.001
	4	2.16 [1.71, 2.61]	<0.001	2.46 [2.11, 2.81]	<0.001
	5 (richest)	1.09 [0.63, 1.55]	<0.001	2.33 [1.97, 2.70]	<0.001
Female literacy rate	1 (poorest)	0.00 (Ref.)		0.00 (Ref.)	
	2	1.52 [1.10, 1.94]	<0.001	0.70 [0.40, 1.00]	<0.001
	3	2.10 [1.66, 2.53]	<0.001	1.33 [1.00, 1.67]	<0.001
	4	1.50 [1.05, 1.95]	<0.001	1.29 [0.94, 1.64]	<0.001
	5 (richest)	1.64 [1.17, 2.11]	<0.001	0.19 [−0.19, 0.57]	0.336

**Abbreviations:** Ref. = Reference category; NFHS-4 = National Family Health Survey 4; DLHS-4 = District-Level Household Survey 4; AHS = Annual Health Survey.

a All multilevel linear regression models i) had obesity as outcome variable; ii) contained a random intercept for district; iii) had five-year age group, sex, urban/rural residency as level 1 (the individual level) independent variables; and iv) district-level median household wealth, Gross Domestic Product (GDP) per capita, the percentage of participants in a district living in an urban area, and district female literacy rate as level 2 (the district level) independent variable.

b The numbers in square brackets are 95% confidence intervals.

c These models included educational attainment as level 1 independent variable and an interaction term between educational attainment and the district-level indicator.

d These models included household wealth quintile as level 1 independent variable and an interaction term between household wealth quintile and the district-level indicator.

**Table 7:** Results from multilevel linear regressions for individual level variables: Obesity.^a^^,^^b^

	NFHS-4		DLHS-4/AHS	
	Absolute difference (% points)	P	Absolute difference (% points)	P
**Interaction with educational attainment**^c^				
**Age group**				
15–19 years	0.00 (Ref.)		0.00 (Ref.)	
20–24 years	2.14 [1.92, 2.36]	<0.001	1.45 [1.21, 1.70]	<0.001
25–29 years	6.19 [5.97, 6.41]	<0.001	4.72 [4.47, 4.96]	<0.001
30–34 years	10.60 [10.37, 10.83]	<0.001	7.84 [7.59, 8.09]	<0.001
35–39 years	13.55 [13.32, 13.79]	<0.001	9.62 [9.37, 9.87]	<0.001
40–44 years	15.98 [15.73, 16.22]	<0.001	10.90 [10.64, 11.15]	<0.001
45–49 years	17.23 [16.98, 17.49]	<0.001	11.64 [11.38, 11.90]	<0.001
50–54 years	13.76 [13.12, 14.39]	<0.001	12.05 [11.78, 12.32]	<0.001
55–50 years	–	–	11.85 [11.57, 12.13]	<0.001
60–64 years	–	–	11.43 [11.14, 11.72]	<0.001
>65 years	–	–	9.39 [9.13, 9.65]	<0.001
**Educational attainment**				
< primary	0.00 (Ref.)		Ref.	
Primary completed	4.08 [3.81, 4.35]	<0.001	3.69 [3.54, 3.85]	<0.001
Some secondary	5.95 [5.78, 6.12]	<0.001	5.49 [5.36, 5.61]	<0.001
Secondary completed	6.51 [6.26, 6.77]	<0.001	6.14 [5.96, 6.32]	<0.001
> secondary	6.76 [6.52, 6.99]	<0.001	7.44 [7.25, 7.62]	<0.001
**Urban area**	6.19 [6.04, 6.35]	<0.001	5.41 [5.30, 5.52]	<0.001
**Female**	3.74 [3.55, 3.93]	<0.001	3.64 [3.55, 3.74]	<0.001
**Interaction with household wealth quintile computed in each district**^d^				
**Age group**				
15–19 years	0.00 (Ref.)		0.00 (Ref.)	
20–24 years	1.29 [1.08, 1.51]	<0.001	1.18 [0.93, 1.44]	<0.001
25–29 years	4.67 [4.46, 4.89]	<0.001	3.92 [3.67, 4.18]	<0.001
30–34 years	8.66 [8.44, 8.88]	<0.001	6.72 [6.46, 6.97]	<0.001
35–39 years	11.11 [10.88, 11.33]	<0.001	8.15 [7.89, 8.40]	<0.001
40–44 years	12.93 [12.70, 13.17]	<0.001	8.99 [8.74, 9.25]	<0.001
45–49 years	13.53 [13.30, 13.77]	<0.001	9.27 [9.01, 9.53]	<0.001
50–54 years	10.29 [9.67, 10.92]	<0.001	9.29 [9.02, 9.56]	<0.001
55–50 years	–	–	8.86 [8.58, 9.14]	<0.001
60–64 years	–	–	8.33 [8.04, 8.62]	<0.001
>65 years	–	–	5.84 [5.59, 6.10]	<0.001
**Household wealth quintile**				
1 (poorest)	0.00 (Ref.)		Ref.	
2	2.03 [1.83, 2.22]	<0.001	1.76 [1.61, 1.91]	<0.001
3	3.98 [3.79, 4.18]	<0.001	3.40 [3.25, 3.55]	<0.001
4	6.17 [5.98, 6.37]	<0.001	5.48 [5.34, 5.63]	<0.001
5 (richest)	9.89 [9.69, 10.08]	<0.001	9.16 [9.01, 9.31]	<0.001
**Urban area**	7.29 [7.14, 7.44]	<0.001	6.80 [6.69, 6.91]	<0.001
**Female**	2.82 [2.64, 3.00]	<0.001	2.52 [2.42, 2.61]	<0.001
**Interaction with household wealth quintile computed nationally**^d^				
**Age group**				
15–19 years	0.00 (Ref.)		0.00 (Ref.)	
20–24 years	1.27 [1.05, 1.48]	<0.001	1.19 [0.94, 1.45]	<0.001
25–29 years	4.63 [4.41, 4.85]	<0.001	3.94 [3.69, 4.19]	<0.001
30–34 years	8.60 [8.38, 8.83]	<0.001	6.72 [6.47, 6.97]	<0.001
35–39 years	11.05 [10.82, 11.27]	<0.001	8.15 [7.89, 8.40]	<0.001
40–44 years	12.87 [12.64, 13.11]	<0.001	8.98 [8.73, 9.24]	<0.001
45–49 years	13.47 [13.23, 13.71]	<0.001	9.25 [8.99, 9.51]	<0.001
50–54 years	10.19 [9.56, 10.81]	<0.001	9.26 [8.99, 9.53]	<0.001
55–50 years	–	–	8.84 [8.56, 9.12]	<0.001
60–64 years	–	–	8.32 [8.03, 8.61]	<0.001
>65 years	–	–	5.85 [5.59, 6.10]	<0.001
**Household wealth quintile**				
1 (poorest)	0.00 (Ref.)		Ref.	
2	2.60 [2.39, 2.80]	<0.001	1.85 [1.69, 2.00]	<0.001
3	4.77 [4.56, 4.98]	<0.001	3.71 [3.55, 3.87]	<0.001
4	7.41 [7.19, 7.62]	<0.001	6.26 [6.09, 6.42]	<0.001
5 (richest)	12.12 [11.90, 12.35]	<0.001	11.53 [11.35, 11.70]	<0.001
**Urban area**	8.64 [8.48, 8.79]	<0.001	7.86 [7.75, 7.97]	<0.001
**Female**	2.80 [2.62, 2.98]	<0.001	2.50 [2.40, 2.59]	<0.001

**Abbreviations:** Ref. = Reference category; NFHS-4 = National Family Health Survey 4; DLHS-4 = District-Level Household Survey 4; AHS = Annual Health Survey.

a All multilevel linear regression models i) had obesity as outcome variable; ii) contained a random intercept for district; iii) had five-year age group, sex, urban/rural residency as level 1 (the individual level) independent variables.

b The numbers in square brackets are 95% confidence intervals.

c These models included educational attainment as level 1 independent variable.

d These models included household wealth quintile as level 1 independent variable.

**Table 8:** Results from multilevel linear regressions for the interaction between district-level socio-economic development and participants’ education and household wealth: Diabetes.^a^^,^^b^

		NFHS-4		DLHS-4/AHS	
		Absolute difference (% points)	P	Absolute difference (% points)	P
**Interaction of the district-level indicators with educational attainment**^c^					
Median household wealth	< primary	0.00 (Ref.)		0.00 (Ref.)	
	Primary completed	−0.22 [−0.55, 0.11]	0.186	0.40 [−0.70, −0.11]	0.007
	Some secondary	−0.50 [−0.69, −0.30]	<0.000	−1.17 [−1.40, −0.94]	<0.001
	Secondary completed	−1.17 [−1.46, −0.88]	<0.000	−2.05 [−2.37, −1.73]	<0.001
	> secondary	−1.35 [−1.63, −1.07]	<0.000	−2.59 [−2.93, −2.26]	<0.001
GDP/capita	< primary	0.00 (Ref.)		0.00 (Ref.)	
	Primary completed	−0.08 [−0.46, 0.30]	0.674	−0.39 [−0.71, −0.07]	0.016
	Some secondary	−0.26 [−0.48, −0.03]	0.023	−0.76 [−1.01, −0.52]	<0.001
	Secondary completed	−0.77 [−1.09, −0.45]	<0.001	−1.92 [−2.26, −1.57]	<0.001
	> secondary	−1.06 [−1.36, −0.76]	<0.001	−2.14 [−2.50, −1.79]	<0.001
% of participants who live in an urban area	< primary	0.00 (Ref.)		0.00 (Ref.)	
	Primary completed	−0.26 [−0.61, 0.09]	0.140	−0.62 [−0.93, −0.32]	<0.001
	Some secondary	−0.82 [−1.02, −0.62]	<0.001	−1.29 [−1.52, −1.06]	<0.001
	Secondary completed	−1.36 [−1.65, −1.07]	<0.001	−2.51 [−2.83, −2.18]	<0.001
	> secondary	−1.63 [−1.90, −1.37]	<0.001	−2.88 [−3.20, −2.55]	<0.001
Female literacy rate	< primary	0.00 (Ref.)		0.00 (Ref.)	
	Primary completed	−0.26 [−0.60, 0.09]	0.145	−0.77 [−1.08, −0.46]	<0.001
	Some secondary	−0.57 [−0.77, −0.38]	<0.001	−1.09 [−1.33, −0.85]	<0.001
	Secondary completed	−1.14 [−1.45, −0.83]	<0.001	−2.26 [−2.61, −1.91]	<0.001
	> secondary	−1.31 [−1.60, −1.02]	<0.001	−2.61 [−2.97, −2.25]	<0.001
**Interaction of the district-level indicators with household wealth quintile computed in each district**^d^					
Median household wealth	1 (poorest)	0.00 (Ref.)		0.00 (Ref.)	
	2	0.14 [−0.10, 0.38]	0.237	0.47 [0.19, 0.75]	0.001
	3	0.01 [−0.23, 0.25]	0.928	0.52 [0.24, 0.81]	<0.001
	4	−0.02 [−0.26, 0.22]	0.878	0.64 [0.36, 0.93]	<0.001
	5 (richest)	−0.16 [−0.40, 0.09]	0.206	0.41 [0.12, 0.69]	0.005
GDP/capita	1 (poorest)	0.00 (Ref.)		0.00 (Ref.)	
	2	0.17 [−0.11, 0.44]	0.235	0.50 [0.19, 0.81]	0.001
	3	0.03 [−0.24, 0.31]	0.814	0.52 [0.21, 0.83]	0.001
	4	0.11 [−0.17, 0.38]	0.446	0.70 [0.40, 1.01]	<0.001
	5 (richest)	0.03 [−0.25, 0.30]	0.841	0.74 [0.43, 1.05]	<0.001
% of participants who live in an urban area	1 (poorest)	0.00 (Ref.)		0.00 (Ref.)	
	2	0.46 [0.21, 0.70]	<0.001	0.52 [0.23, 0.81]	<0.001
	3	0.37 [0.13, 0.61]	0.003	0.77 [0.48, 1.05]	<0.001
	4	0.31 [0.07, 0.56]	0.011	0.98 [0.70, 1.27]	<0.001
	5 (richest)	0.21 [−0.03, 0.46]	0.089	1.14 [0.86, 1.43]	<0.001
Female literacy rate	1 (poorest)	0.00 (Ref.)		0.00 (Ref.)	
	2	0.29 [0.05, 0.53]	0.018	0.26 [−0.03, 0.54]	0.083
	3	0.11 [−0.13, 0.35]	0.388	0.11 [−0.18, 0.40]	0.470
	4	0.11 [−0.13, 0.35]	0.363	0.16 [−0.13, 0.45]	0.282
	5 (richest)	0.08 [−0.17, 0.32]	0.538	0.32 [0.03, 0.60]	0.032
**Interaction of the district-level indicators with household wealth quintile computed nationally**^d^					
Median household wealth	1 (poorest)	0.00 (Ref.)		0.00 (Ref.)	
	2	−0.01 [−0.32, 0.29]	0.929	0.57 [0.19, 0.95]	0.004
	3	−0.01 [−0.33, 0.30]	0.926	0.28 [−0.13, 0.68]	0.183
	4	−0.32 [−0.64, −0.01]	0.046	0.42 [0.02, 0.82]	0.042
	5 (richest)	−0.33 [−0.65, −0.02]	0.038	−0.25 [−0.66, 0.16]	0.233
GDP/capita	1 (poorest)	0.00 (Ref.)		0.00 (Ref.)	
	2	0.30 [−0.02, 0.62]	0.067	0.63 [0.27, 1.00]	0.001
	3	0.23 [−0.10, 0.55]	0.171	0.43 [0.05, 0.81]	0.025
	4	0.06 [−0.26, 0.38]	0.709	0.77 [0.38, 1.15]	<0.001
	5 (richest)	0.14 [−0.18, 0.47]	0.390	0.33 [−0.07, 0.73]	0.101
% of participants who live in an urban area	1 (poorest)	0.00 (Ref.)		0.00 (Ref.)	
	2	0.27 [0.00, 0.54]	0.050	0.38 [0.07, 0.69]	0.016
	3	0.36 [0.09, 0.63]	0.009	0.49 [0.17, 0.82]	0.003
	4	0.24 [−0.04, 0.51]	0.090	0.62 [0.29, 0.96]	<0.001
	5 (richest)	0.23 [−0.06, 0.51]	0.117	0.43 [0.07, 0.78]	0.019
Female literacy rate	1 (poorest)	0.00 (Ref.)		0.00 (Ref.)	
	2	0.08 [−0.18, 0.34]	0.547	0.20 [−0.09, 0.49]	0.172
	3	0.22 [−0.04, 0.49]	0.103	0.02 [−0.31, 0.34]	0.924
	4	0.06 [−0.21, 0.34]	0.647	0.10 [−0.24, 0.44]	0.557
	5 (richest)	0.26 [−0.03, 0.54]	0.079	−0.30 [−0.67, 0.06]	0.106

**Abbreviations:** Ref. = Reference category; NFHS-4 = National Family Health Survey 4; DLHS-4 = District-Level Household Survey 4; AHS = Annual Health Survey.

a All multilevel linear regression models i) had diabetes as outcome variable; ii) contained a random intercept for district; iii) had five-year age group, sex, urban/rural residency as level 1 (the individual level) independent variables; and iv) district-level median household wealth, Gross Domestic Product (GDP) per capita, the percentage of participants in a district living in an urban area, and district female literacy rate as level 2 (the district level) independent variable.

b The numbers in square brackets are 95% confidence intervals.

c These models included educational attainment as level 1 independent variable and an interaction term between educational attainment and the district-level indicator.

d These models included household wealth quintile as level 1 independent variable and an interaction term between household wealth quintile and the district-level indicator.

**Table 9:** Results from multilevel linear regressions for individual-level variables: Diabetes.^a^^,^^b^

	NFHS-4		DLHS-4/AHS	
	Absolute difference (% points)	P	Absolute difference (% points)	P
**Interaction with educational attainment**^c^				
**Age group**				
15–19 years	0.00 (Ref.)		0.00 (Ref.)	
20–24 years	0.38 [0.25, 0.52]	<0.001	0.33 [0.09, 0.57]	0.007
25–29 years	0.92 [0.79, 1.06]	<0.001	1.34 [1.10, 1.59]	<0.001
30–34 years	1.90 [1.76, 2.04]	<0.001	2.81 [2.57, 3.05]	<0.001
35–39 years	3.18 [3.04, 3.33]	<0.001	4.25 [4.00, 4.49]	<0.001
40–44 years	5.26 [5.11, 5.41]	<0.001	6.03 [5.78, 6.27]	<0.001
45–49 years	7.62 [7.46, 7.77]	<0.001	7.92 [7.67, 8.18]	<0.001
50–54 years	10.60 [10.21, 10.98]	<0.001	10.05 [9.79, 10.31]	<0.001
55–50 years	–	–	11.48 [11.21, 11.76]	<0.001
60–64 years	–	–	12.79 [12.51, 13.07]	<0.001
>65 years	–	–	13.46 [13.21, 13.72]	<0.001
**Educational attainment**				
< primary	0.00 (Ref.)		Ref.	
Primary completed	0.87 [0.70, 1.03]	<0.001	1.56 [1.41, 1.71]	<0.001
Some secondary	1.15 [1.04, 1.25]	<0.001	2.04 [1.92, 2.16]	<0.001
Secondary completed	0.99 [0.83, 1.14]	<0.001	1.54 [1.36, 1.71]	<0.001
> secondary	0.85 [0.70, 0.99]	<0.001	1.44 [1.27, 1.62]	<0.001
**Urban area**	1.21 [1.12, 1.31]	<0.001	2.24 [2.14, 2.35]	<0.001
**Female**	−0.57 [−0.68, −0.45]	<0.001	0.00 [−0.09, 0.10]	0.931
**Interaction with household wealth quintile computed in each district**^d^				
**Age group**				
15–19 years	0.00 (Ref.)		0.00 (Ref.)	
20–24 years	0.14 [0.01, 0.27]	0.037	0.13 [−0.11, 0.38]	0.294
25–29 years	0.60 [0.47, 0.73]	<0.001	1.02 [0.77, 1.27]	<0.001
30–34 years	1.52 [1.39, 1.66]	<0.001	2.44 [2.19, 2.69]	<0.001
35–39 years	2.72 [2.58, 2.86]	<0.001	3.82 [3.57, 4.06]	<0.001
40–44 years	4.70 [4.55, 4.84]	<0.001	5.48 [5.23, 5.73]	<0.001
45–49 years	6.94 [6.80, 7.09]	<0.001	7.23 [6.98, 7.49]	<0.001
50–54 years	9.97 [9.58, 10.35]	<0.001	9.30 [9.04, 9.56]	<0.001
55–50 years	–	–	10.66 [10.39, 10.94]	<0.001
60–64 years	–	–	11.93 [11.64, 12.21]	<0.001
>65 years	–	–	12.48 [12.23, 12.74]	<0.001
**Household wealth quintile**				
1 (poorest)	0.00 (Ref.)		0.00 (Ref.)	
2	0.23 [0.11, 0.35]	<0.001	0.60 [0.46, 0.74]	<0.001
3	0.60 [0.48, 0.72]	<0.001	1.07 [0.92, 1.21]	<0.001
4	0.97 [0.85, 1.09]	<0.001	1.76 [1.61, 1.90]	<0.001
5 (richest)	1.68 [1.55, 1.80]	<0.001	2.91 [2.77, 3.06]	<0.001
**Urban area**	1.36 [1.27, 1.45]	<0.001	2.57 [2.47, 2.68]	<0.001
**Female**	−0.72 [−0.83, −0.60]	<0.001	−0.31 [−0.40, −0.22]	<0.001
**Interaction with household wealth quintile computed nationally**^d^				
**Age group**				
15–19 years	0.00 (Ref.)		0.00 (Ref.)	
20–24 years	0.14 [0.00, 0.27]	0.045	0.14 [−0.11, 0.38]	0.274
25–29 years	0.59 [0.46, 0.73]	<0.001	1.03 [0.78, 1.28]	<0.001
30–34 years	1.51 [1.38, 1.65]	<0.001	2.45 [2.20, 2.69]	<0.001
35–39 years	2.71 [2.57, 2.85]	<0.001	3.82 [3.57, 4.07]	<0.001
40–44 years	4.69 [4.54, 4.83]	<0.001	5.49 [5.24, 5.74]	<0.001
45–49 years	6.93 [6.79, 7.08]	<0.001	7.23 [6.98, 7.49]	<0.001
50–54 years	9.95 [9.57, 10.33]	<0.001	9.30 [9.04, 9.56]	<0.001
55–50 years	–	–	10.66 [10.39, 10.94]	<0.001
60–64 years	–	–	11.93 [11.65, 12.21]	<0.001
>65 years	–	–	12.50 [12.25, 12.75]	<0.001
**Household wealth quintile**				
1 (poorest)	0.00 (Ref.)		0.00 (Ref.)	
2	0.41 [0.28, 0.53]	<0.001	0.83 [0.68, 0.98]	<0.001
3	0.79 [0.66, 0.91]	<0.001	1.50 [1.35, 1.66]	<0.001
4	1.27 [1.14, 1.40]	<0.001	2.23 [2.07, 2.39]	<0.001
5 (richest)	2.00 [1.87, 2.14]	<0.001	3.68 [3.51, 3.85]	<0.001
**Urban area**	1.57 [1.47, 1.66]	<0.001	2.92 [2.81, 3.03]	<0.001
**Female**	−0.72 [−0.83, −0.61]	<0.001	−0.32 [−0.41, −0.23]	<0.001

**Abbreviations:** Ref. = Reference category; NFHS-4 = National Family Health Survey 4; DLHS-4 = District-Level Household Survey 4; AHS = Annual Health Survey.

a All multilevel linear regression models i) had diabetes as outcome variable; ii) contained a random intercept for district; iii) had five-year age group, sex, urban/rural residency as level 1 (the individual level) independent variables.

b The numbers in square brackets are 95% confidence intervals.

c These models included educational attainment as level 1 independent variable.

d These models included household wealth quintile as level 1 independent variable.

**Table 10:** Results from multilevel linear regressions for the interaction between district-level socio-economic development and participants’ education and household wealth: Currently smoking.^a^^,^^b^

		NFHS-4		DLHS-4/AHS	
		Absolute difference (% points)	P	Absolute difference (% points)	P
**Interaction of the district-level indicators with educational attainment**^c^					
Median household wealth	< primary	0.00 (Ref.)		0.00 (Ref.)	
	Primary completed	−0.49 [−0.86, −0.11]	0.011	1.64 [1.28, 1.99]	<0.001
	Some secondary	−1.01 [−1.23, −0.79]	<0.001	2.82 [2.55, 3.09]	<0.001
	Secondary completed	−0.86 [−1.19, −0.53]	<0.001	3.70 [3.31, 4.08]	<0.001
	> secondary	−0.84 [−1.16, −0.53]	<0.001	5.31 [4.91, 5.71]	<0.001
GDP/capita	< primary	0.00 (Ref.)		0.00 (Ref.)	
	Primary completed	0.03 [−0.41, 0.46]	0.897	1.39 [0.99, 1.78]	<0.001
	Some secondary	0.00 [−0.26, 0.25]	0.978	2.57 [2.27, 2.87]	<0.001
	Secondary completed	0.56 [0.19, 0.93]	0.003	4.07 [3.65, 4.49]	<0.001
	> secondary	0.86 [0.52, 1.21]	<0.001	5.42 [4.99, 5.86]	<0.001
% of participants who live in an urban area	< primary	0.00 (Ref.)		0.00 (Ref.)	
	Primary completed	0.49 [0.10, 0.88]	0.015	1.44 [1.07, 1.80]	<0.001
	Some secondary	0.16 [−0.07, 0.38]	0.173	2.31 [2.04, 2.59]	<0.001
	Secondary completed	0.72 [0.39, 1.04]	<0.001	3.71 [3.33, 4.10]	<0.001
	> secondary	0.88 [0.58, 1.18]	<0.001	4.62 [4.23, 5.00]	<0.001
Female literacy rate	< primary	0.00 (Ref.)		0.00 (Ref.)	
	Primary completed	0.25 [−0.14, 0.64]	0.215	1.90 [1.53, 2.26]	<0.001
	Some secondary	−0.16 [−0.38, 0.07]	0.172	3.16 [2.88, 3.44]	<0.001
	Secondary completed	0.46 [0.10, 0.81]	0.012	4.70 [4.28, 5.11]	<0.001
	> secondary	1.02 [0.68, 1.35]	<0.001	6.54 [6.11, 6.96]	<0.001
**Interaction of the district-level indicators with household wealth quintile computed in each district**^d^					
Median household wealth	1 (poorest)	0.00 (Ref.)		0.00 (Ref.)	
	2	−0.93 [−1.20, −0.66]	<0.001	−1.30 [−1.63, −0.97]	<0.001
	3	−1.14 [−1.42, −0.87]	<0.001	−1.73 [−2.06, −1.40]	<0.001
	4	−1.43 [−1.70, −1.15]	<0.001	−1.64 [−2.97, −1.31]	<0.001
	5 (richest)	−1.42 [−1.69, −1.15]	<0.001	−1.08 [−1.41, −0.75]	<0.001
GDP/capita	1 (poorest)	0.00 (Ref.)		0.00 (Ref.)	
	2	−0.24 [−0.55, 0.08]	0.137	−0.74 [−1.10, −0.37]	<0.001
	3	−0.18 [−0.49, 0.14]	0.265	−0.85 [−1.22, −0.48]	<0.001
	4	−0.24 [−0.55, 0.07]	0.135	−1.10 [−1.46, −0.73]	<0.001
	5 (richest)	0.00 [−0.32, 0.31]	0.992	−0.29 [−0.65, 0.08]	0.126
% of participants who live in an urban area	1 (poorest)	0.00 (Ref.)		0.00 (Ref.)	
	2	−0.12 [−0.40, 0.15]	0.381	−0.51 [−0.84, −0.18]	0.003
	3	0.00 [−0.27, 0.27]	0.999	−0.78 [−1.11, −0.45]	<0.001
	4	0.00 [−0.27, 0.28]	0.977	−0.51 [−0.84, −0.18]	<0.001
	5 (richest)	−0.03 [−0.30, 0.25]	0.854	0.42 [−0.09, 0.75]	0.013
Female literacy rate	1 (poorest)	0.00 (Ref.)		0.00 (Ref.)	
	2	−0.41 [−0.68, −0.13]	0.004	−0.54 [−0.87, −0.21]	0.001
	3	−0.33 [−0.60, −0.06]	0.018	−0.48 [−0.81, −0.15]	0.005
	4	−0.47 [−0.74, −0.19]	0.001	−0.37 [−0.70, −0.04]	0.028
	5 (richest)	−0.22 [−0.49, 0.06]	0.120	0.20 [−0.14, 0.53]	0.247
**Interaction of the district-level indicators with household wealth quintile computed nationally**^d^					
Median household wealth	1 (poorest)	0.00 (Ref.)		0.00 (Ref.)	
	2	−0.43 [−0.78, −0.08]	0.016	−0.55 [−0.99, −0.11]	0.015
	3	−0.70 [−1.06, −0.34]	<0.001	−0.13 [−0.60, 0.34]	0.591
	4	−1.02 [−1.38, −0.65]	<0.001	−0.39 [−0.86, 0.08]	0.102
	5 (richest)	−2.30 [−2.67, −1.93]	<0.001	−1.79 [−2.27, −1.32]	<0.001
GDP/capita	1 (poorest)	0.00 (Ref.)		0.00 (Ref.)	
	2	0.25 [−0.12, 0.62]	0.179	−0.36 [−0.78, 0.07]	0.100
	3	0.13 [−0.24, 0.50]	0.492	0.14 [−0.31, 0.59]	0.549
	4	0.33 [−0.04, 0.70]	0.082	0.44 [−0.01, 0.89]	0.054
	5 (richest)	−0.19 [−0.56, 0.19]	0.333	−0.13 [−0.60, 0.35]	0.596
% of participants who live in an urban area	1 (poorest)	0.00 (Ref.)		0.00 (Ref.)	
	2	0.08 [−0.23, 0.39]	0.618	−0.06 [−0.42, 0.29]	0.719
	3	−0.03 [−0.34, 0.28]	0.860	0.45 [0.08, 0.82]	0.017
	4	0.24 [−0.07, 0.55]	0.132	0.51 [0.12, 0.89]	0.010
	5 (richest)	0.08 [−0.24, 0.41]	0.605	1.19 [0.78, 1.61]	<0.001
Female literacy rate	1 (poorest)	0.00 (Ref.)		0.00 (Ref.)	
	2	−0.03 [−0.33, 0.26]	0.832	−0.74 [−1.07, −0.41]	<0.001
	3	−0.41 [−0.72, −0.11]	0.008	−0.10 [−0.47, 0.27]	0.594
	4	−0.07 [−0.39, 0.25]	0.678	0.22 [−0.16, 0.61]	0.256
	5 (richest)	−0.52 [−0.85, −0.19]	0.002	0.64 [0.21, 1.06]	0.003

**Abbreviations:** Ref. = Reference category; NFHS-4 = National Family Health Survey 4; DLHS-4 = District-Level Household Survey 4; AHS = Annual Health Survey.

a All multilevel linear regression models i) had currently smoking as outcome variable; ii) contained a random intercept for district; iii) had five-year age group, sex, urban/rural residency as level 1 (the individual level) independent variables; and iv) district-level median household wealth, Gross Domestic Product (GDP) per capita, the percentage of participants in a district living in an urban area, and district female literacy rate as level 2 (the district level) independent variable.

b The numbers in square brackets are 95% confidence intervals.

c These models included educational attainment as level 1 independent variable and an interaction term between educational attainment and the district-level indicator.

d These models included household wealth quintile as level 1 independent variable and an interaction term between household wealth quintile and the district-level indicator.

**Table 11:** Results from multilevel linear regressions for individual-level variables: Currently smoking.^a^^,^^b^

	NFHS-4		DLHS-4/AHS	
	Absolute difference (% points)	P	Absolute difference (% points)	P
**Interaction with educational attainment**^c^				
**Age group**				
15–19 years	0.00 (Ref.)		0.00 (Ref.)	
20–24 years	2.11 [1.96, 2.27]	<0.001	1.48 [1.15, 1.81]	<0.001
25–29 years	2.62 [2.47, 2.78]	<0.001	4.98 [4.65, 5.31]	<0.001
30–34 years	3.08 [2.92, 3.24]	<0.001	7.05 [6.72, 7.38]	<0.001
35–39 years	3.70 [3.54, 3.87]	<0.001	8.54 [8.21, 8.88]	<0.001
40–44 years	4.50 [4.33, 4.67]	<0.001	9.55 [9.21, 9.88]	<0.001
45–49 years	5.07 [4.89, 5.25]	<0.001	10.47 [10.13, 10.81]	<0.001
50–54 years	14.73 [14.29, 15.17]	<0.001	10.41 [10.06, 10.76]	<0.001
55-50 years	–	–	10.32 [9.96, 10.69]	<0.001
60–64 years	–	–	9.98 [9.61, 10.35]	<0.001
>65 years	–	–	7.69 [7.35, 8.03]	<0.001
**Educational attainment**				
< primary	0.00 (Ref.)		0.00 (Ref.)	
Primary completed	−0.79 [−0.98, −0.61]	<0.001	−1.76 [−1.94, −1.59]	<0.001
Some secondary	−1.94 [−2.06, −1.83]	<0.001	−4.15 [−4.29, −4.00]	<0.001
Secondary completed	−2.98 [−3.16, −2.81]	<0.001	−6.53 [−6.74, −6.32]	<0.001
> secondary	−4.05 [−4.21, −3.88]	<0.001	−8.08 [−8.29, −7.87]	<0.001
**Urban area**	−0.37 [−0.48, −0.26]	<0.001	−1.12 [−1.25, −0.99]	<0.001
**Female**	−25.55 [−25.68, −25.43]	<0.001	−21.87 [−21.98, −21.77]	<0.001
**Interaction with household wealth quintile computed in each district**^d^				
**Age group**				
15–19 years	0.00 (Ref.)		0.00 (Ref.)	
20–24 years	2.04 [1.89, 2.19]	<0.001	1.74 [1.41, 2.06]	<0.001
25–29 years	2.96 [2.81, 3.11]	<0.001	5.87 [5.55, 6.20]	<0.001
30–34 years	3.67 [3.51, 3.82]	<0.001	8.39 [8.06, 8.71]	<0.001
35–39 years	4.52 [4.36, 4.67]	<0.001	10.23 [9.90, 10.56]	<0.001
40–44 years	5.55 [5.39, 5.72]	<0.001	11.61 [11.28, 11.94]	<0.001
45–49 years	6.38 [6.21, 6.55]	<0.001	12.94 [12.60, 13.27]	<0.001
50–54 years	15.94 [15.50, 16.37]	<0.001	13.20 [12.86, 13.55]	<0.001
55–50 years	–	–	13.29 [12.93, 13.64]	<0.001
60–64 years	–	–	13.07 [12.70, 13.43]	<0.001
>65 years	–	–	11.12 [10.79, 11.45]	<0.001
**Household wealth quintile**				
1 (poorest)	0.00 (Ref.)		0.00 (Ref.)	
2	−1.09 [−1.23, −0.95]	<0.001	−1.54 [−1.70, −1.37]	<0.001
3	−1.93 [−2.06, −1.79]	<0.001	−2.76 [−2.92, −2.59]	<0.001
4	−2.58 [−2.72, −2.44]	<0.001	−4.15 [−4.31, −3.98]	<0.001
5 (richest)	−3.37 [−3.51, −3.23]	<0.001	−5.95 [−6.12, −5.79]	<0.001
**Urban area**	−1.00 [−1.11, −0.90]	<0.001	−2.55 [−2.67, −2.43]	<0.001
**Female**	−25.15 [−25.28, −25.02]	<0.001	−20.77 [−20.87, −20.66]	<0.001
**Interaction with household wealth quintile computed nationally**^d^				
**Age group**				
15–19 years	0.00 (Ref.)		0.00 (Ref.)	
20–24 years	2.04 [1.89, 2.19]	<0.001	1.70 [1.37, 2.02]	<0.001
25–29 years	2.97 [2.81, 3.12]	<0.001	5.83 [5.50, 6.16]	<0.001
30–34 years	3.69 [3.53, 3.84]	<0.001	8.35 [8.02, 8.67]	<0.001
35–39 years	4.54 [4.38, 4.70]	<0.001	10.20 [9.87, 10.53]	<0.001
40–44 years	5.57 [5.41, 5.73]	<0.001	11.59 [11.26, 11.92]	<0.001
45–49 years	6.39 [6.23, 6.56]	<0.001	12.92 [12.58, 13.26]	<0.001
50–54 years	15.96 [15.53, 16.40]	<0.001	13.19 [12.85, 13.54]	<0.001
55–50 years	–	–	13.28 [12.92, 13.63]	<0.001
60–64 years	–	–	13.04 [12.68, 13.41]	<0.001
>65 years	–	–	11.08 [10.75, 11.41]	<0.001
**Household wealth quintile**				
1 (poorest)	0.00 (Ref.)		0.00 (Ref.)	
2	−0.93 [−1.08, −0.79]	<0.001	−2.04 [−2.21, −1.87]	<0.001
3	−1.74 [−1.89, −1.60]	<0.001	−3.56 [−3.74, −3.39]	<0.001
4	−2.60 [−2.75, −2.45]	<0.001	−5.10 [−5.29, −4.92]	<0.001
5 (richest)	−3.76 [−3.92, −3.61]	<0.001	−7.78 [−7.98, −7.59]	<0.001
**Urban area**	−1.43 [−1.54, −1.32]	<0.001	−3.23 [−3.35, −3.11]	<0.001
**Female**	−25.14 [−25.27, −25.01]	<0.001	−20.76 [−20.87, −20.66]	<0.001

**Abbreviations:** Ref. = Reference category; NFHS-4 = National Family Health Survey 4; DLHS-4 = District-Level Household Survey 4; AHS = Annual Health Survey.

a All multilevel linear regression models i) had currently smoking as outcome variable; ii) contained a random intercept for district; iii) had five-year age group, sex, urban/rural residency as level 1 (the individual level) independent variables.

b The numbers in square brackets are 95% confidence intervals.

c These models included educational attainment as level 1 independent variable.

d These models included household wealth quintile as level 1 independent variable.

**Table 12:** Results from multilevel linear regressions for the interaction between district-level socio-economic development and participants’ education and household wealth: High blood pressure (NFHS-4).^a^^,^^b^

		*NFHS-4*	
		Absolute difference (% points)	P
**Interaction of the district-level indicators with educational attainment**^c^			
% of participants who completed primary education	< primary	0.00 (Ref.)	
	Primary completed	−0.45 [−1.08, 0.18]	0.159
	Some secondary	−0.83 [−1.20, −0.47]	<0.001
	Secondary completed	−1.25 [−1.81, −0.69]	<0.001
	> secondary	−1.46 [−1.99, −0.92]	<0.001
Median household wealth	< primary	0.00 (Ref.)	
	Primary completed	0.25 [−0.34, 0.85]	0.403
	Some secondary	0.00 [−0.35, 0.36]	0.990
	Secondary completed	−0.55 [−1.07, −0.02]	0.042
	> secondary	−0.97 [−1.48, −0.46]	<0.001
GDP/capita	< primary	0.00 (Ref.)	
	Primary completed	−0.96 [−1.64, −0.28]	0.006
	Some secondary	−1.34 [−1.74, −0.94]	<0.001
	Secondary completed	−2.02 [−2.60, −1.45]	<0.001
	> secondary	−2.50 [−3.04, −1.95]	<0.001
% of participants who live in an urban area	< primary	0.00 (Ref.)	
	Primary completed	0.26 [−0.37, 0.89]	0.417
	Some secondary	0.07 [−0.29, 0.44]	0.686
	Secondary completed	−0.21 [−0.74, 0.32]	0.432
	> secondary	−0.76 [−1.24, −0.27]	0.002
Female literacy rate	< primary	0.00 (Ref.)	
	Primary completed	−0.87 [−1.50, −0.24]	0.007
	Some secondary	−1.18 [−1.54, −0.82]	<0.001
	Secondary completed	−1.59 [−2.16, −1.03]	<0.001
	> secondary	−1.89 [−2.42, −1.36]	<0.001
**Interaction of the district-level indicators with household wealth quintile computed in each district**^d^			
% of participants who completed primary education	1 (poorest)	0.00 (Ref.)	
	2	0.66 [0.23, 1.10]	0.003
	3	0.50 [0.06, 0.93]	0.025
	4	0.12 [−0.31, 0.56]	0.583
	5 (richest)	−0.72 [−1.16, −0.28]	0.001
Median household wealth	1 (poorest)	0.00 (Ref.)	
	2	0.83 [0.39, 1.27]	<0.001
	3	0.94 [0.50, 1.38]	<0.001
	4	0.68 [0.25, 1.12]	0.002
	5 (richest)	0.19 [−0.25, 0.63]	0.390
GDP/capita	1 (poorest)	0.00 (Ref.)	
	2	0.46 [−0.04, 0.95]	0.069
	3	0.20 [−0.29, 0.69]	0.425
	4	−0.46 [−0.96, 0.03]	0.065
	5 (richest)	−0.94 [−1.43, −0.44]	<0.001
% of participants who live in an urban area	1 (poorest)	0.00 (Ref.)	
	2	0.26 [−0.18, 0.70]	0.249
	3	0.43 [−0.01, 0.87]	0.055
	4	0.07 [−0.37, 0.51]	0.749
	5 (richest)	−0.34 [−0.78, 0.11]	0.135
Female literacy rate	1 (poorest)	0.00 (Ref.)	
	2	0.57 [0.13, 1.01]	0.011
	3	0.35 [−0.09, 0.79]	0.117
	4	−0.01 [−0.44, 0.43]	0.978
	5 (richest)	−0.79 [−1.23, −0.35]	<0.001

**Abbreviations:** Ref. = Reference category; NFHS-4 = National Family Health Survey 4; DLHS-4 = District-Level Household Survey 4; AHS = Annual Health Survey.

a All multilevel linear regression models i) had high blood pressure as outcome variable; ii) contained a random intercept for district; iii) had five-year age group, sex, urban/rural residency as level 1 (the individual level) independent variables; and iv) district-level primary school completion rate, district-level median household wealth, Gross Domestic Product (GDP) per capita, the percentage of participants in a district living in an urban area, and district female literacy rate as level 2 (the district level) independent variable.

b The numbers in square brackets are 95% confidence intervals.

c These models included educational attainment as level 1 independent variable and an interaction term between educational attainment and the district-level indicator.

d These models included household wealth quintile as level 1 independent variable and an interaction term between household wealth quintile and the district-level indicator.

**Table 13:** Results from multilevel linear regressions for individual-level variables: High blood pressure (NFHS-4).^a^^,^^b^

	NFHS-4	
	Absolute difference (% points)	P
**Interaction with educational attainment**^c^		
**Age group**		
15–19 years	0.00 (Ref.)	
20–24 years	2.00 [1.75, 2.24]	<0.001
25–29 years	4.66 [4.41, 4.90]	<0.001
30–34 years	8.68 [8.42, 8.93]	<0.001
35–39 years	13.23 [12.97, 13.49]	<0.001
40–44 years	17.96 [17.68, 18.23]	<0.001
45–49 years	22.62 [22.33, 22.90]	<0.001
50–54 years	24.32 [23.64, 25.04]	<0.001
55–50 years	–	–
60–64 years	–	–
>65 years	–	–
**Educational attainment**		
< primary	0.00 (Ref.)	
Primary completed	0.71 [0.41, 1.10]	<0.001
Some secondary	0.69 [0.50, 0.87]	<0.001
Secondary completed	0.57 [0.29, 0.86]	<0.001
> secondary	0.21 [−0.05, 0.47]	0.113
**Urban area**	1.45 [1.28, 1.62]	<0.001
**Female**	−4.90 [−5.11, −4.70]	<0.001
**Interaction of the district-level indicators with household wealth quintile computed in each district**^d^		
**Age group**		
15–19 years	0.00 (Ref.)	
20–24 years	1.77 [1.53, 2.01]	<0.001
25–29 years	4.42 [4.18, 4.66]	<0.001
30–34 years	8.44 [8.19, 8.69]	<0.001
35–39 years	12.95 [12.70, 13.20]	<0.001
40–44 years	17.61 [17.35, 17.88]	<0.001
45–49 years	22.20 [21.93, 22.46]	<0.001
50–54 years	23.94 [23.25, 24.64]	<0.001
55–50 years	–	–
60–64 years	–	–
>65 years	–	–
**Household wealth quintile**		
1 (poorest)	0.00 (Ref.)	
2	0.41 [0.19, 0.63]	<0.001
3	0.78 [0.56, 1.00]	<0.001
4	1.08 [0.86, 1.30]	<0.001
5 (richest)	1.63 [1.41, 1.85]	<0.001
**Urban area**	1.50 [1.33, 1.67]	<0.001
**Female**	−4.98 [−5.18, −4.77]	<0.001

**Abbreviations:** Ref. = Reference category; NFHS-4 = National Family Health Survey 4; DLHS-4 = District-Level Household Survey 4; AHS = Annual Health Survey.

a All multilevel linear regression models i) had high blood pressure as outcome variable; ii) contained a random intercept for district; iii) had five-year age group, sex, urban/rural residency as level 1 (the individual level) independent variables.

b The numbers in square brackets are 95% confidence intervals.

c These models included educational attainment as level 1 independent variable.

d These models included household wealth quintile as level 1 independent variable.

**Table 14:** Results from multilevel linear regressions for the interaction between district-level socio-economic development and participants’ education and household wealth: High blood glucose (NFHS-4).^a^^,^^b^

		*NFHS-4*	
		Absolute difference (% points)	P
**Interaction of the district-level indicators with educational attainment**^c^			
% of participants who completed primary education	< primary	0.00 (Ref.)	
	Primary completed	0.03 [−0.25, 0.31]	0.827
	Some secondary	−0.28 [−0.44, −0.12]	0.001
	Secondary completed	−0.70 [−0.95, −0.45]	<0.001
	> secondary	−0.88 [−1.12, −0.64]	<0.001
Median household wealth	< primary	0.00 (Ref.)	
	Primary completed	0.15 [−0.12, 0.42]	0.271
	Some secondary	−0.24 [−0.39, −0.08]	0.003
	Secondary completed	−0.68 [−0.91, −0.44]	<0.001
	> secondary	−0.82 [−1.05, −0.60]	<0.001
GDP/capita	< primary	0.00 (Ref.)	
	Primary completed	0.04 [−0.27, 0.35]	0.820
	Some secondary	−0.16 [−0.34, 0.02]	0.085
	Secondary completed	−0.53 [−0.79, −0.27]	<0.001
	> secondary	−0.74 [−0.98, −0.49]	<0.001
% of participants who live in an urban area	< primary	0.00 (Ref.)	
	Primary completed	−0.06 [−0.34, 0.22]	0.681
	Some secondary	−0.51 [−0.67, −0.34]	<0.001
	Secondary completed	−0.85 [−1.09, −0.62]	<0.001
	> secondary	−1.11 [−1.33, −0.89]	<0.001
Female literacy rate	< primary	0.00 (Ref.)	
	Primary completed	0.03 [−0.25, 0.31]	0.836
	Some secondary	−0.28 [−0.44, −0.12]	0.001
	Secondary completed	−0.67 [−0.92, −0.42]	<0.001
	> secondary	−0.81 [−1.05, −0.58]	<0.001
**Interaction of the district-level indicators with household wealth quintile computed in each district**^d^			
% of participants who completed primary education	1 (poorest)	0.00 (Ref.)	
	2	0.23 [0.04, 0.43]	0.020
	3	0.08 [−0.12, 0.27]	0.450
	4	0.11 [−0.08, 0.31]	0.265
	5 (richest)	−0.13 [−0.33, 0.06]	0.182
Median household wealth	1 (poorest)	0.00 (Ref.)	
	2	0.21 [0.01, 0.40]	0.040
	3	0.11 [−0.09, 0.31]	0.274
	4	0.05 [−0.15, 0.24]	0.639
	5 (richest)	−0.18 [−0.38, 0.01]	0.065
GDP/capita	1 (poorest)	0.00 (Ref.)	
	2	0.14 [−0.09, 0.36]	0.236
	3	0.07 [−0.15, 0.30]	0.533
	4	0.06 [−0.17, 0.28]	0.616
	5 (richest)	−0.09 [−0.31, 0.13]	0.430
% of participants who live in an urban area	1 (poorest)	0.00 (Ref.)	
	2	0.28 [0.08, 0.48]	0.006
	3	0.24 [0.04, 0.44]	0.018
	4	0.25 [0.06, 0.45]	0.012
	5 (richest)	0.07 [−0.13, 0.27]	0.494
Female literacy rate	1 (poorest)	0.00 (Ref.)	
	2	0.25 [0.05, 0.44]	0.013
	3	0.11 [−0.09, 0.30]	0.282
	4	0.14 [−0.05, 0.34]	0.147
	5 (richest)	−0.05 [−0.24, 0.15]	0.650

**Abbreviations:** Ref. = Reference category; NFHS-4 = National Family Health Survey 4; DLHS-4 = District-Level Household Survey 4; AHS = Annual Health Survey.

a All multilevel linear regression models i) had high blood glucose as outcome variable; ii) contained a random intercept for district; iii) had five-year age group, sex, urban/rural residency as level 1 (the individual level) independent variables; and iv) district-level primary school completion rate, district-level median household wealth, Gross Domestic Product (GDP) per capita, the percentage of participants in a district living in an urban area, and district female literacy rate as level 2 (the district level) independent variable.

b The numbers in square brackets are 95% confidence intervals.

c These models included educational attainment as level 1 independent variable and an interaction term between educational attainment and the district-level indicator.

d These models included household wealth quintile as level 1 independent variable and an interaction term between household wealth quintile and the district-level indicator.

**Table 15:** Results from multilevel linear regressions for individual-level variables: High blood glucose (NFHS-4).^a^^,^^b^

	NFHS-4	
	Absolute difference (% points)	P
**Interaction with educational attainment**^c^		
**Age group**		
15–19 years	0.00 (Ref.)	
20–24 years	0.25 [0.14, 0.36]	<0.001
25–29 years	0.58 [0.47, 0.69]	<0.001
30–34 years	1.23 [1.12, 1.34]	<0.001
35–39 years	2.16 [2.04, 2.28]	<0.001
40–44 years	3.58 [3.46, 3.71]	<0.001
45–49 years	5.16 [5.03, 5.29]	<0.001
50–54 years	7.13 [6.82, 7.45]	<0.001
55–50 years	–	–
60–64 years	–	–
>65 years	–	–
**Educational attainment**		
< primary	0.00 (Ref.)	
Primary completed	0.53 [0.39, 0.66]	<0.001
Some secondary	0.70 [0.62, 0.79]	<0.001
Secondary completed	0.51 [0.38, 0.63]	<0.001
> secondary	0.43 [0.32, 0.55]	<0.001
**Urban area**	0.86 [0.78, 0.94]	<0.001
**Female**	−0.52 [−0.61, −0.42]	<0.001
**Interaction of the district-level indicators with household wealth quintile computed in each district**^d^		
**Age group**		
15–19 years	0.00 (Ref.)	
20–24 years	0.08 [−0.03, 0.19]	0.139
25–29 years	0.37 [0.26, 0.48]	<0.001
30–34 years	1.00 [0.89, 1.11]	<0.001
35–39 years	1.88 [1.77, 2.00]	<0.001
40–44 years	3.25 [3.13, 3.36]	<0.001
45–49 years	4.76 [4.64, 4.88]	<0.001
50–54 years	6.75 [6.44, 7.07]	<0.001
55–50 years	–	–
60–64 years	–	–
>65 years	–	–
**Household wealth quintile**		
1 (poorest)	0.00 (Ref.)	
2	0.14 [0.04, 0.24]	<0.001
3	0.38 [0.28, 0.48]	<0.001
4	0.66 [0.56, 0.75]	<0.001
5 (richest)	1.01 [0.91, 1.11]	<0.001
**Urban area**	0.94 [0.86, 1.01]	<0.001
**Female**	−0.60 [−0.69, −0.51]	<0.001

**Abbreviations:** Ref. = Reference category; NFHS-4 = National Family Health Survey 4; DLHS-4 = District-Level Household Survey 4; AHS = Annual Health Survey.

a All multilevel linear regression models i) had high blood glucose as outcome variable; ii) contained a random intercept for district; iii) had five-year age group, sex, urban/rural residency as level 1 (the individual level) independent variables.

b The numbers in square brackets are 95% confidence intervals.

c These models included educational attainment as level 1 independent variable.

d These models included household wealth quintile as level 1 independent variable.

**Table 16:** Results from multilevel linear regressions for the interaction between district-level socio-economic development and participants’ education and household wealth: Diabetes (assuming AHS participants were not fasted).^a^^,^^b^

		*DLHS-4 AHS*	
		Absolute difference (% points)	P
**Interaction of the district-level indicators with educational attainment**^c^			
% of participants who completed primary education	< primary	0.00 (Ref.)	
	Primary completed	−1.01 [−1.29, −0.72]	<0.001
	Some secondary	−1.67 [−1.89, −1.45]	<0.001
	Secondary completed	−3.11 [−3.43, −2.79]	<0.001
	> secondary	−3.07 [−3.40, −2.75]	<0.001
Median household wealth	< primary	0.00 (Ref.)	
	Primary completed	−0.81 [−1.08, −0.54]	<0.001
	Some secondary	−1.65 [−1.86, −1.45]	<0.001
	Secondary completed	−2.65 [−2.94, −2.35]	<0.001
	> secondary	−2.43 [−2.74, −2.13]	<0.001
GDP/capita	< primary	0.00 (Ref.)	
	Primary completed	−0.72 [−1.00, −0.43]	<0.001
	Some secondary	−1.19 [−1.41, −0.98]	<0.001
	Secondary completed	−2.51 [−2.82, −2.20]	<0.001
	> secondary	−2.27 [−2.59, −1.95]	<0.001
% of participants who live in an urban area	< primary	0.00 (Ref.)	
	Primary completed	−0.84 [−1.12, −0.56]	<0.001
	Some secondary	−1.65 [−1.87, −1.44]	<0.001
	Secondary completed	−2.89 [−3.18, −2.59]	<0.001
	> secondary	−2.74 [−3.03, −2.44]	<0.001
Female literacy rate	< primary	0.00 (Ref.)	
	Primary completed	−0.91 [−1.19, −0.63]	<0.001
	Some secondary	−1.38 [−1.60, −1.16]	<0.001
	Secondary completed	−2.75 [−3.07, −2.43]	<0.001
	> secondary	−2.75 [−3.08, −2.43]	<0.001
**Interaction of the district-level indicators with household wealth quintile computed in each district**^d^			
% of participants who completed primary education	1 (poorest)	0.00 (Ref.)	
	2	0.36 [0.09, 0.62]	0.008
	3	0.37 [0.10, 0.63]	0.007
	4	0.49 [0.22, 0.75]	<0.001
	5 (richest)	0.78 [0.52, 1.05]	<0.001
Median household wealth	1 (poorest)	0.00 (Ref.)	
	2	0.47 [0.21, 0.73]	<0.001
	3	0.63 [0.37, 0.89]	<0.001
	4	0.87 [0.61, 1.14]	<0.001
	5 (richest)	1.25 [0.99, 1.51]	<0.001
GDP/capita	1 (poorest)	0.00 (Ref.)	
	2	0.53 [0.25, 0.81]	<0.001
	3	0.66 [0.38, 0.94]	<0.001
	4	0.88 [0.60, 1.16]	<0.001
	5 (richest)	1.31 [1.03, 1.59]	<0.001
% of participants who live in an urban area	1 (poorest)	0.00 (Ref.)	
	2	0.50 [0.24, 0.76]	<0.001
	3	0.84 [0.58, 1.10]	<0.001
	4	1.26 [1.00, 1.53]	<0.001
	5 (richest)	1.89 [1.62, 2.15]	<0.001
Female literacy rate	1 (poorest)	0.00 (Ref.)	
	2	0.24 [−0.02, 0.51]	0.074
	3	0.19 [−0.07, 0.46]	0.157
	4	0.29 [0.02, 0.56]	0.033
	5 (richest)	0.60 [0.33, 0.87]	<0.001

**Abbreviations:** Ref. = Reference category; NFHS-4 = National Family Health Survey 4; DLHS-4 = District-Level Household Survey 4; AHS = Annual Health Survey.

a All multilevel linear regression models i) had diabetes as outcome variable (assuming AHS participants were not fasted); ii) contained a random intercept for district; iii) had five-year age group, sex, urban/rural residency as level 1 (the individual level) independent variables; and iv) district-level primary school completion rate, district-level median household wealth, Gross Domestic Product (GDP) per capita, the percentage of participants in a district living in an urban area, and district female literacy rate as level 2 (the district level) independent variable.

b The numbers in square brackets are 95% confidence intervals.

c These models included educational attainment as level 1 independent variable and an interaction term between educational attainment and the district-level indicator.

d These models included household wealth quintile as level 1 independent variable and an interaction term between household wealth quintile and the district-level indicator.

**Table 17:** Results from multilevel linear regressions for individual-level variables: Diabetes (assuming AHS participants were not fasted).^a^^,^^b^

	DLHS-4/AHS	
	Absolute difference (% points)	P
**Interaction with educational attainment**^c^		
**Age group**		
15–19 years	0.00 (Ref.)	
20–24 years	0.22 [0.00, 0.44]	0.048
25–29 years	1.11 [0.89, 1.33]	0.002
30–34 years	2.42 [2.20, 2.64]	<0.001
35–39 years	3.54 [3.32, 3.77]	<0.001
40–44 years	5.08 [4.86, 5.31]	<0.001
45–49 years	6.57 [6.34, 6.80]	<0.001
50–54 years	8.30 [8.06, 8.53]	<0.001
55–50 years	9.35 [9.10, 9.60]	<0.001
60–64 years	10.44 [10.18, 10.69]	<0.001
>65 years	10.69 [10.45, 10.92]	<0.001
**Educational attainment**		
< primary	0.00 (Ref.)	
Primary completed	1.30 [1.17, 1.44]	<0.001
Some secondary	1.63 [1.52, 1.74]	<0.001
Secondary completed	0.82 [0.66, 0.98]	<0.001
> secondary	0.53 [0.37, 0.69]	<0.001
**Urban area**	1.94 [1.84, 2.03]	<0.001
**Female**	0.04 [−0.05, 0.12]	0.401
**Interaction of the district-level indicators with household wealth quintile computed in each district**^d^		
**Age group**		
15–19 years	0.00 (Ref.)	
20–24 years	0.01 [−0.22, 0.24]	0.927
25–29 years	0.82 [0.60, 1.05]	<0.001
30–34 years	2.15 [1.92, 2.38]	<0.001
35–39 years	3.27 [3.04, 3.50]	<0.001
40–44 years	4.78 [4.54, 5.01]	<0.001
45–49 years	6.20 [5.96, 6.43]	<0.001
50–54 years	7.90 [7.66, 8.14]	<0.001
55–50 years	8.94 [8.69, 9.20]	<0.001
60–64 years	10.03 [9.77, 10.29]	<0.001
>65 years	10.23 [10.00, 10.46]	<0.001
**Household wealth quintile**		
1 (poorest)	0.00 (Ref.)	
2	0.54 [0.41, 0.67]	<0.001
3	0.92 [0.79, 1.05]	<0.001
4	1.55 [1.42, 1.68]	<0.001
5 (richest)	2.42 [2.29, 2.55]	<0.001
**Urban area**	2.14 [2.05, 2.24]	<0.001
**Female**	−0.18 [−0.27, −0.10]	<0.001

**Abbreviations:** Ref. = Reference category; NFHS-4 = National Family Health Survey 4; DLHS-4 = District-Level Household Survey 4; AHS = Annual Health Survey.

a All multilevel linear regression models i) had diabetes as outcome variable (assuming AHS participants were not fasted); ii) contained a random intercept for district; iii) had five-year age group, sex, urban/rural residency as level 1 (the individual level) independent variables.

b The numbers in square brackets are 95% confidence intervals.

c These models included educational attainment as level 1 independent variable.

d These models included household wealth quintile as level 1 independent variable.

**Table 18:** Results from multilevel linear regressions for the interaction between district-level socio-economic development and participants’ education and household wealth: Currently smoking (men only).^a^^,^^b^^,^^c^

		*NFHS-4*		*DLHS-4/AHS*	
		Absolute difference (% points)	P	Absolute difference (% points)	P
**Interaction of the district-level indicators with educational attainment**^d^					
% of participants who completed primary education	< primary	0.00 (Ref.)		0.00 (Ref.)	
	Primary completed	0.45 [−1.83, 2.73]	0.700	2.88 [2.19, 3.58]	<0.001
	Some secondary	−1.16 [−2.54, 0.21]	0.097	4.30 [3.76, 4.85]	<0.001
	Secondary completed	0.14 [−1.76, 2.04]	0.886	5.22 [4.47, 5.97]	<0.001
	> secondary	−0.99 [−2.76, 0.77]	0.271	7.33 [6.58, 8.08]	<0.001
Median household wealth	< primary	0.00 (Ref.)		0.00 (Ref.)	
	Primary completed	−2.36 [−4.62, −0.10]	0.040	2.56 [1.88, 3.24]	<0.001
	Some secondary	−6.13 [−7.52, −4.75]	<0.001	3.65 [3.12, 4.17]	<0.001
	Secondary completed	−6.12 [−7.96, −4.29]	<0.001	4.24 [3.54, 4.93]	<0.001
	> secondary	−8.54 [−10.30, −6.78]	<0.001	5.42 [4.70, 6.14]	<0.001
GDP/capita	< primary	0.00 (Ref.)		0.00 (Ref.)	
	Primary completed	−0.32 [−3.05, 2.41]	0.818	2.54 [1.78, 3.30]	<0.001
	Some secondary	−1.79 [−3.49, −0.08]	0.040	3.74 [3.16, 4.33]	<0.001
	Secondary completed	−0.06 [−2.22, 2.11]	0.959	5.23 [4.46, 6.01]	<0.001
	> secondary	−0.93 [−2.97, 1.11]	0.371	6.55 [5.77, 7.33]	<0.001
% of participants who live in an urban area	< primary	0.00 (Ref.)		0.00 (Ref.)	
	Primary completed	−2.16 [−4.48, 0.16]	0.068	1.54 [0.83, 2.25]	<0.001
	Some secondary	−2.25 [−3.66, −0.84]	0.002	2.91 [2.37, 4.46]	<0.001
	Secondary completed	0.85 [−1.02, 2.71]	0.373	4.60 [3.89, 5.32]	<0.001
	> secondary	−0.98 [−2.69, 0.74]	0.263	5.82 [5.13, 6.52]	<0.001
Female literacy rate	< primary	0.00 (Ref.)		0.00 (Ref.)	
	Primary completed	0.53 [−1.72, 2.78]	0.646	2.83 [2.16, 3.50]	<0.001
	Some secondary	−0.05 [−1.41, 1.32]	0.946	4.20 [3.67, 4.73]	<0.001
	Secondary completed	1.62 [−0.27, 3.52]	0.094	5.45 [4.71, 6.20]	<0.001
	> secondary	0.58 [−1.18, 2.34]	0.519	7.37 [6.63, 8.11]	<0.001
**Interaction of the district-level indicators with household wealth quintile computed in each district**^e^					
% of participants who completed primary education	1 (poorest)	0.00 (Ref.)		0.00 (Ref.)	
	2	−1.77 [−3.32, −0.22]	0.025	−1.51 [−2.14, −0.88]	<0.001
	3	−3.39 [−4.94, −1.84]	<0.001	−2.07 [−2.70, −1.44]	<0.001
	4	−3.05 [−4.60, −1.51]	<0.001	−1.35 [−1.98, −0.73]	<0.001
	5 (richest)	−2.92 [−4.46, −1.38]	<0.001	−0.72 [−1.34, −0.09]	0.025
Median household wealth	1 (poorest)	0.00 (Ref.)		0.00 (Ref.)	
	2	−4.22 [−5.78, −2.67]	<0.001	−2.62 [−3.23, −2.00]	<0.001
	3	−6.12 [−7.67, −4.57]	<0.001	−3.83 [−4.45, −3.21]	<0.001
	4	−6.81 [−8.35, −5.27]	<0.001	−3.74 [−4.36, −3.12]	<0.001
	5 (richest)	−7.15 [−8.69, −5.62]	<0.001	−2.86 [−3.48, −2.24]	<0.001
GDP/capita	1 (poorest)	0.00 (Ref.)		0.00 (Ref.)	
	2	−1.18 [−3.01, 0.66]	0.209	−1.88 [−2.57, −1.19]	<0.001
	3	−1.64 [−3.48, 0.20]	0.081	−2.35 [−3.04, −1.66]	<0.001
	4	−1.57 [−3.39, 0.25]	0.092	−2.92 [−3.62, −2.23]	<0.001
	5 (richest)	−1.35 [−3.15, 0.46]	0.143	−1.44 [−2.13, −0.74]	<0.001
% of participants who live in an urban area	1 (poorest)	0.00 (Ref.)		0.00 (Ref.)	
	2	−1.06 [−2.60, 0.48]	0.178	−1.25 [−1.87, −0.63]	<0.001
	3	−1.67 [−3.21, −0.12]	0.034	−2.25 [−2.88, −1.63]	<0.001
	4	−1.03 [−2.58, 0.51]	0.191	−1.81 [−2.43, −1.19]	<0.001
	5 (richest)	−1.69 [−3.23, −0.16]	0.030	−0.38 [−1.01, 0.24]	0.226
Female literacy rate	1 (poorest)	0.00 (Ref.)		0.00 (Ref.)	
	2	−1.15 [−2.70, 0.40]	0.145	−1.20 [−1.82, −0.58]	<0.001
	3	−2.47 [−4.01, −0.92]	0.002	−1.37 [−2.00, −0.75]	<0.001
	4	−2.33 [−3.87, −0.78]	0.003	−1.11 [−1.73, −0.48]	0.001
	5 (richest)	−2.02 [−3.56, −0.48]	0.010	−0.38 [−1.01, 0.24]	0.226

**Abbreviations:** Ref. = Reference category; NFHS-4 = National Family Health Survey 4; DLHS-4 = District-Level Household Survey 4; AHS = Annual Health Survey.

a All multilevel linear regression models i) had currently smoking as outcome variable; ii) contained a random intercept for district; iii) had five-year age group, sex, urban/rural residency as level 1 (the individual level) independent variables; and iv) district-level primary school completion rate, district-level median household wealth, Gross Domestic Product (GDP) per capita, the percentage of participants in a district living in an urban area, and district female literacy rate as level 2 (the district level) independent variable.

b The numbers in square brackets are 95% confidence intervals.

c In this analysis only male participants were included.

d These models included educational attainment as level 1 independent variable and an interaction term between educational attainment and the district-level indicator.

e These models included household wealth quintile as level 1 independent variable and an interaction term between household wealth quintile and the district-level indicator.

**Table 19:** Results from multilevel linear regressions for individual-level variables: Currently smoking (men only).^a^^,^^b^^,^^c^

	NFHS-4		DLHS-4/AHS	
	Absolute difference (% points)	P	Absolute difference (% points)	P
**Interaction with educational attainment**^d^				
**Age group**				
15–19 years	0.00 (Ref.)		0.00 (Ref.)	
20–24 years	14.30 [13.41, 15.19]	<0.001	3.01 [2.40, 3.61]	<0.001
25–29 years	18.27 [17.38, 19.15]	<0.001	9.72 [9.10, 10.33]	<0.001
30–34 years	19.91 [19.00, 20.82]	<0.001	13.83 [13.22, 14.45]	<0.001
35–39 years	21.85 [20.92, 22.77]	<0.001	16.92 [16.30, 17.54]	<0.001
40–44 years	24.01 [23.05, 24.98]	<0.001	18.67 [18.05, 19.29]	<0.001
45–49 years	24.63 [23.64, 25.62]	<0.001	20.03 [19.40, 20.67]	<0.001
50–54 years	25.73 [24.64, 26.81]	<0.001	19.50 [18.86, 20.15]	<0.001
55–50 years	–	–	18.80 [18.13, 19.46]	<0.001
60–64 years	–	–	17.31 [16.63, 18.00]	<0.001
>65 years	–	–	12.57 [11.94, 13.20]	<0.001
**Educational attainment**				
< primary	0.00 (Ref.)		0.00 (Ref.)	
Primary completed	−2.78 [−3.89, −1.66]	<0.001	−4.16 [−4.49, −3.83]	<0.001
Some secondary	−10.30 [−10.99, −9.61]	<0.001	−8.68 [−8.95, −8.41]	<0.001
Secondary completed	−16.18 [−17.12, −15.23]	<0.001	−12.70 [−13.07, −12.33]	<0.001
> secondary	−21.74 [−22.62, −20.86]	<0.001	−15.24 [−15.60–14.87]	<0.001
**Urban area**	−0.63 [−1.21, −0.05]	0.032	−1.84 [−2.08, −1.60]	<0.001
**Interaction of the district-level indicators with household wealth quintile computed in each district**^e^				
**Age group**				
15–19 years	0.00 (Ref.)		0.00 (Ref.)	
20–24 years	12.43 [11.56, 13.30]	<0.001	3.34 [2.74, 3.95]	<0.001
25–29 years	17.97 [17.09, 18.85]	<0.001	10.82 [10.21, 11.43]	<0.001
30–34 years	20.61 [19.71, 21.51]	<0.001	15.64 [15.01, 16.25]	<0.001
35–39 years	23.30 [22.39, 24.21]	<0.001	19.18 [18.56, 19.80]	<0.001
40–44 years	26.11 [25.15, 27.06]	<0.001	21.47 [20.85, 22.09]	<0.001
45–49 years	27.81 [26.83, 28.78]	<0.001	23.59 [22.96, 24.22]	<0.001
50–54 years	29.92 [28.86, 30.98]	<0.001	23.71 [23.07, 24.35]	<0.001
55–50 years	–	–	23.38 [22.72, 24.04]	<0.001
60–64 years	–	–	22.28 [21.61, 22.96]	<0.001
>65 years	–	–	18.31 [17.70, 18.93]	<0.001
**Household wealth quintile**				
1 (poorest)	0.00 (Ref.)		0.00 (Ref.)	
2	−3.68 [−4.45, −2.90]	<0.001	−2.74 [−3.06, −2.43]	<0.001
3	−7.38 [−8.15, −6.60]	<0.001	−4.89 [−5.20, −4.58]	<0.001
4	−10.29 [−11.06, −9.51]	<0.001	−7.37 [−7.68, −7.06]	<0.001
5 (richest)	−13.54 [−14.31, −12.77]	<0.001	−10.61 [−10.92, −10.30]	<0.001
**Urban area**	−3.24 [−3.82, −2.67]	<0.001	−4.49 [−4.73, −4.26]	<0.001

**Abbreviations:** Ref. = Reference category; NFHS-4 = National Family Health Survey 4; DLHS-4 = District-Level Household Survey 4; AHS = Annual Health Survey.

a All multilevel linear regression models i) had currently smoking as outcome variable; ii) contained a random intercept for district; iii) had five-year age group, sex, urban/rural residency as level 1 (the individual level) independent variables.

b The numbers in square brackets are 95% confidence intervals.

c In this analysis only male participants were included.

d These models included educational attainment as level 1 independent variable.

e These models included household wealth quintile as level 1 independent variable.

**Table 20:** Results from multilevel linear regressions for the interaction between district-level socio-economic development and participants’ education and household wealth: Currently smoking (women only).^a^^,^^b^^,^^c^

		*NFHS-4*		*DLHS-4/AHS*	
		Absolute difference (% points)	P	Absolute difference (% points)	P
**Interaction of the district-level indicators with educational attainment**^d^					
% of participants who completed primary education	< primary	0.00 (Ref.)		0.00 (Ref.)	
	Primary completed	0.21 [−0.03, 0.44]	0.087	0.65 [0.39, 0.90]	<0.001
	Some secondary	−0.18 [−0.31, −0.04]	0.011	0.38 [0.18, 0.58]	<0.001
	Secondary completed	−0.21 [−0.42, 0.01]	0.061	0.31 [0.00, 0.62]	0.049
	> secondary	0.03 [−0.18, 0.23]	0.794	0.46 [0.24, 0.80]	0.007
Median household wealth	< primary	0.00 (Ref.)		0.00 (Ref.)	
	Primary completed	−0.50 [−0.72, −0.28]	<0.001	0.34 [0.11, 0.58]	0.004
	Some secondary	−0.65 [−0.78, −0.51]	<0.001	0.39 [0.20, 0.58]	<0.001
	Secondary completed	−0.54 [−0.74, −0.34]	<0.001	0.73 [0.45, 1.00]	<0.001
	> secondary	−0.48 [−0.67, −0.28]	<0.001	0.73 [0.43, 1.03]	<0.001
GDP/capita	< primary	0.00 (Ref.)		0.00 (Ref.)	
	Primary completed	0.03 [−0.23, 0.30]	0.816	0.39 [0.12, 0.65|	0.004
	Some secondary	0.10 [−0.05, 0.26]	0.192	0.64 [0.44, 0.85]	<0.001
	Secondary completed	0.21 [−0.02, 0.44]	0.070	0.91 [0.60, 1.22]	<0.001
	> secondary	0.43 [0.22, 0.65]	<0.001	0.86 [0.52, 1.19]	<0.001
% of participants who live in an urban area	< primary	0.00 (Ref.)		0.00 (Ref.)	
	Primary completed	0.83 [0.60, 1.07]	<0.001	1.03 [0.79, 1.27]	<0.001
	Some secondary	0.61 [0.48, 0.75]	<0.001	1.18 [0.99, 1.36]	<0.001
	Secondary completed	0.55 [0.36, 0.75]	<0.001	1.31 [1.04, 1.58]	<0.001
	> secondary	0.74 [0.56, 0.92]	<0.001	1.34 [1.06, 1.63]	<0.001
Female literacy rate	< primary	0.00 (Ref.)		0.00 (Ref.)	
	Primary completed	0.40 [0.16, 0.63]	0.001	0.91 [0.66, 1.16]	<0.001
	Some secondary	−0.17 [−0.30, −0.03]	0.017	0.04 [−0.16, 0.24]	0.708
	Secondary completed	−0.27 [−0.49, −0.06]	0.012	−0.12 [−0.43, 0.18]	0.433
	> secondary	0.02 [−0.19, 0.22]	0.870	−0.03 [−0.36, 0.30]	0.845
**Interaction of the district-level indicators with household wealth quintile computed in each district**^e^					
% of participants who completed primary education	1 (poorest)	0.00 (Ref.)		0.00 (Ref.)	
	2	−0.07 [−0.24, 0.09]	0.376	0.08 [−0.14, 0.30]	0.473
	3	0.13 [−0.04, 0.29]	0.132	0.29 [0.07, 0.51]	0.010
	4	0.09 [−0.07, 0.26]	0.257	0.29 [0.07, 0.51]	0.010
	5 (richest)	0.33 [0.16, 0.49]	<0.001	0.60 [0.38, 0.77]	<0.001
Median household wealth	1 (poorest)	0.00 (Ref.)		0.00 (Ref.)	
	2	−0.22 [−0.39, −0.06]	0.008	−0.02 [−0.24, 0.20]	0.881
	3	−0.16 [−0.33, 0.00]	0.049	0.15 [−0.07, 0.37]	0.173
	4	−0.22 [−0.38, −0.06]	0.009	0.14 [−0.08, 0.36]	0.222
	5 (richest)	−0.07 [−0.23, 0.10]	0.424	0.32 [0.10, 0.54]	0.004
GDP/capita	1 (poorest)	0.00 (Ref.)		0.00 (Ref.)	
	2	0.05 [−0.14, 0.25]	0.589	0.28 [0.03, 0.53]	0.026
	3	0.16 [−0.03, 0.35]	0.105	0.39 [0.14, 0.63]	0.002
	4	0.22 [0.02, 0.41]	0.027	0.39 [0.15, 0.64]	0.002
	5 (richest)	0.49 [0.29, 0.68]	<0.001	0.45 [0.20, 0.69]	<0.001
% of participants who live in an urban area	1 (poorest)	0.00 (Ref.)		0.00 (Ref.)	
	2	0.09 [−0.08, 0.25]	0.303	0.15 [−0.07, 0.37]	0.188
	3	0.27 [0.11, 0.43]	0.001	0.44 [0.22, 0.66]	<0.001
	4	0.26 [0.09, 0.42]	0.002	0.44 [0.22, 0.66]	<0.001
	5 (richest)	0.36 [0.20, 0.52]	<0.001	0.85 [0.63, 1.07]	<0.001
Female literacy rate	1 (poorest)	0.00 (Ref.)		0.00 (Ref.)	
	2	−0.13 [−0.29, 0.04]	0.132	−0.05 [−0.27, 0.17]	0.662
	3	0.12 [−0.04, 0.28]	0.151	0.14 [−0.08, 0.36]	0.218
	4	0.05 [−0.11, 0.22]	0.516	0.04 [−0.19, 0.26]	0.754
	5 (richest)	0.28 [0.12, 0.45]	0.001	0.34 [0.12, 0.56]	0.002

**Abbreviations:** Ref. = Reference category; NFHS-4 = National Family Health Survey 4; DLHS-4 = District-Level Household Survey 4; AHS = Annual Health Survey.

a All multilevel linear regression models i) had currently smoking as outcome variable; ii) contained a random intercept for district; iii) had five-year age group, sex, urban/rural residency as level 1 (the individual level) independent variables; and iv) district-level primary school completion rate, district-level median household wealth, Gross Domestic Product (GDP) per capita, the percentage of participants in a district living in an urban area, district female literacy rate as level 2 (the district level) independent variable.

b The numbers in square brackets are 95% confidence intervals.

c In this analysis only female participants were included.

d These models included educational attainment as level 1 independent variable and an interaction term between educational attainment and the district-level indicator.

e These models included household wealth quintile as level 1 independent variable and an interaction term between household wealth quintile and the district-level indicator.

**Table 21:** Results from multilevel linear regressions for individual-level variables: Currently smoking (women only).^a^^,^^b^^,^^c^

	NFHS-4		DLHS-4/AHS	
	Absolute difference (% points)	P	Absolute difference (% points)	P
**Interaction with educational attainment**^d^				
**Age group**				
15–19 years	0.00 (Ref.)		0.00 (Ref.)	
20–24 years	0.17 [0.08, 0.26]	<0.001	0.35 [0.13, 0.57]	<0.001
25–29 years	0.25 [0.16, 0.34]	<0.001	0.44 [0.22, 0.66]	<0.001
30–34 years	0.52 [0.43, 0.62]	<0.001	0.63 [0.41, 0.86]	<0.001
35–39 years	0.91 [0.81, 1.01]	<0.001	0.96 [0.73, 1.18]	<0.001
40–44 years	1.54 [1.44, 1.64]	<0.001	1.20 [0.98, 1.43]	<0.001
45–49 years	2.11 [2.00, 2.21]	<0.001	1.64 [1.40, 1.87]	<0.001
50–54 years	–	–	2.18 [1.95, 1.42]	<0.001
55–50 years	–	–	2.35 [2.11, 2.60]	<0.001
60–64 years	–	–	2.93 [2.67, 3.18]	<0.001
>65 years	–	–	3.04 [2.81, 3.28]	<0.001
**Educational attainment**				
< primary	0.00 (Ref.)		0.00 (Ref.)	
Primary completed	−1.20 [−1.31, −1.09]	<0.001	−0.83 [−0.95, −0.71]	<0.001
Some secondary	−1.39 [−1.46, −1.32]	<0.001	−1.10 [−1.20, −1.00]	<0.001
Secondary completed	−1.47 [−1.58, −1.37]	<0.001	−1.04 [−1.19, −0.89]	<0.001
> secondary	−1.45 [−1.55, −1.35]	<0.001	−1.06 [−1.21, −0.90]	<0.001
**Urban area**	−0.31 [−0.38, −0.25]	<0.001	−0.50 [−0.58, −0.41]	<0.001
**Interaction of the district-level indicators with household wealth quintile computed in each district**^e^				
**Age group**				
15–19 years	0.00 (Ref.)		0.00 (Ref.)	
20–24 years	0.37 [0.28, 0.46]	<0.001	0.43 [0.21, 0.65]	<0.001
25–29 years	0.60 [0.51, 0.69]	<0.001	0.64 [0.42, 0.86]	<0.001
30–34 years	0.99 [0.89, 1.08]	<0.001	0.91 [0.70, 1.13]	<0.001
35–39 years	1.50 [1.40, 1.59]	<0.001	1.32 [1.10, 1.54]	<0.001
40–44 years	2.26 [2.17, 2.36]	<0.001	1.67 [1.45, 1.89]	<0.001
45–49 years	2.97 [2.87, 3.07]	<0.001	2.19 [1.96, 2.41]	<0.001
50–54 years	–	–	2.80 [2.57, 3.03]	<0.001
55–50 years	–	–	3.00 [2.76, 3.24]	<0.001
60–64 years	–	–	3.59 [3.35, 3.84]	<0.001
>65 years	–	–	3.78 [3.56, 4.01]	<0.001
**Household wealth quintile**				
1 (poorest)	0.00 (Ref.)		0.00 (Ref.)	
2	−0.64 [−0.72, −0.56]	<0.001	−0.49 [−0.60, −0.38]	<0.001
3	−0.99 [−1.07, −0.91]	<0.001	−0.77 [−0.88, −0.66]	<0.001
4	−1.27 [−1.35, −1.19]	<0.001	−1.16 [−1.27, −1.05]	<0.001
5 (richest)	−1.59 [−1.68, −1.51]	<0.001	−1.55 [−1.66, −1.44]	<0.001
**Urban area**	−0.56 [−0.63, −0.50]	<0.001	−0.73 [−0.81, −0.65]	<0.001

**Abbreviations:** Ref. = Reference category; NFHS-4 = National Family Health Survey 4; DLHS-4 = District-Level Household Survey 4; AHS = Annual Health Survey.

a All multilevel linear regression models i) had currently smoking as outcome variable; ii) contained a random intercept for district; iii) had five-year age group, sex, urban/rural residency as level 1 (the individual level) independent variables.

b The numbers in square brackets are 95% confidence intervals.

c In this analysis only female participants were included.

d These models included educational attainment as level 1 independent variable.

e These models included household wealth quintile as level 1 independent variable.

We conducted two additional analyses to improve our understanding of our findings: i) association of a district's primary school completion rate with the difference in the continuous household wealth index between highest and lowest household wealth quintile (Fig. 11a, Fig. 11ba and b), and ii) logistic and linear regressions of CVD risk factors onto household wealth and district-level fixed effects, conducted in the total sample and a subset of the data (Table 22, Table 23, Table 24, Table 25).

**Fig. 11a:**
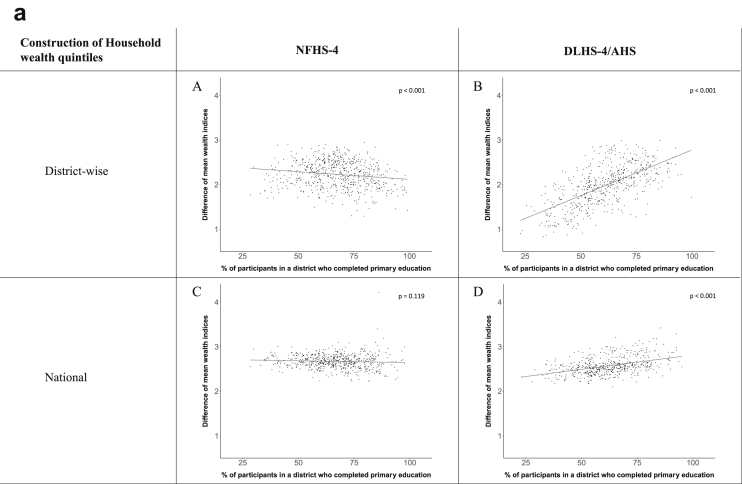
**Association of a district's primary school completion rate with the difference in the continuous household wealth index between highest and lowest household wealth quintile (computed for each district).** The asset score was standardized by subtracting the mean and dividing by one standard deviation. The grey line through the scatterplots has been fitted using ordinary least squares regression (with each data point in the plot having the same weight). The p-value shows whether the slope of the grey line is significantly different from zero. We excluded districts with fewer than 20 participants in the highest or lowest household wealth quintile.

**Fig. 11b:**
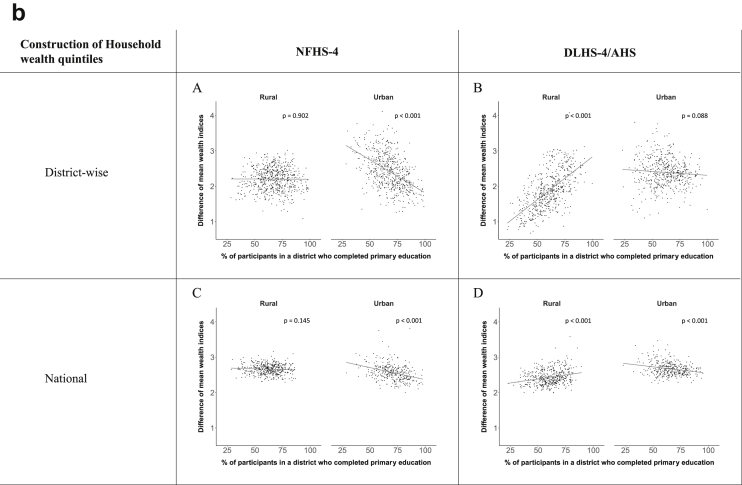
**Association of a district's primary school completion rate with the difference in the continuous household wealth index between highest and lowest household wealth quintile (computed for each district) stratified by urban-rural residency**. The asset score was standardized by subtracting the mean and dividing by one standard deviation. This analysis was performed separately for urban and rural areas. The grey line through the scatterplots has been fitted using ordinary least squares regression (with each data point in the plot having the same weight). The p-value shows whether the slope of the grey line is significantly different from zero. We excluded districts with fewer than 20 participants in the highest or lowest household wealth quintile.

**Table 22:** Logistic regression of CVD risk factors onto household wealth (computed in each district) with district-level fixed effects (NFHS-4).^a^^,^^b^^,^^c^

		all districts		subset^d^	
		Relative difference (Odds ratio)	P	Relative difference (Odds ratio)	P
Household wealth quintile					
Diabetes	1 (poorest)	0.00 (Ref.)		0.00 (Ref.)	
	2	1.09 [1.04, 1.14]	<0.001	0.98 [0.87, 1.11]	0.760
	3	1.26 [1.20, 1.32]	<0.001	1.27 [1.13, 1.42]	<0.001
	4	1.45 [1.39, 1.52]	<0.001	1.47 [1.32, 1.65]	<0.001
	5 (richest)	1.84 [1.76, 1.92]	<0.001	1.92 [1.73, 2.14]	<0.001
Hypertension	1 (poorest)	0.00 (Ref.)		0.00 (Ref.)	
	2	1.05 [1.03, 1.07]	<0.001	1.00 [0.95, 1.05]	0.953
	3	1.12 [1.10, 1.14]	<0.001	1.08 [1.03, 1.13]	0.001
	4	1.20 [1.17, 1.22]	<0.001	1.18 [1.13, 1.24]	<0.001
	5 (richest)	1.33 [1.30, 1.35]	<0.001	1.40 [1.34, 1.47]	<0.001
Obesity	1 (poorest)	0.00 (Ref.)		0.00 (Ref.)	
	2	1.43 [1.39, 1.48]	<0.001	1.40 [1.26, 1.55]	<0.001
	3	1.92 [1.86, 1.98]	<0.001	1.91 [1.73, 2.10]	<0.001
	4	2.51 [2.44, 2.59]	<0.001	2.86 [2.61, 3.14]	<0.001
	5 (richest)	3.65 [3.54, 3.75]	<0.001	5.21 [4.77, 5.68]	<0.001

**Abbreviations:** Ref. = Reference category; NFHS-4 = National Family Health Survey 4.

a All logistic models had diabetes, hypertension or obesity as outcome variables.

b The numbers in square brackets are 95% confidence intervals.

c These models included district household wealth quintile as independent variable.

d The subset were the 20% of the districts with the lowest primary school completion rate.

**Table 23:** Logistic regression of CVD risk factors onto household wealth (computed in each district) with district-level fixed effects (DLHS-4/AHS).^a^^,^^b^^,^^c^

		all districts		subset^d^	
		Relative difference (Odds ratio)	P	Relative difference (Odds ratio)	P
Household wealth quintile					
Diabetes	1 (poorest)	0.00 (Ref.)		0.00 (Ref.)	
	2	1.05 [1.03, 1.07]	<0.001	1.01 [0.94, 1.09]	0.782
	3	1.13 [1.11, 1.16]	<0.001	1.08 [1.01, 1.16]	0.036
	4	1.27 [1.24, 1.29]	<0.001	1.23 [1.14, 1.32]	<0.001
	5 (richest)	1.50 [1.47, 1.54]	<0.001	1.48 [1.38, 1.58]	<0.001
Hypertension	1 (poorest)	0.00 (Ref.)		0.00 (Ref.)	
	2	0.98 [0.97, 1.00]	0.015	0.95 [0.92, 0.98]	0.003
	3	1.03 [1.02, 1.05]	<0.001	0.99 [0.95, 1.02]	0.418
	4	1.08 [1.07, 1.10]	<0.001	1.00 [0.97, 1.03]	0.929
	5 (richest)	1.21 [1.19, 1.22]	<0.001	1.17 [1.14, 1.21]	<0.001
Obesity	1 (poorest)	0.00 (Ref.)		0.00 (Ref.)	
	2	1.36 [1.33, 1.39]	<0.001	1.24 [1.14, 1.35]	<0.001
	3	1.73 [1.69, 1.77]	<0.001	1.45 [1.34, 1.58]	<0.001
	4	2.24 [2.19, 2.29]	<0.001	2.02 [1.86, 2.18]	<0.001
	5 (richest)	3.23 [3.16, 3.29]	<0.001	3.48 [3.23, 3.76]	<0.001

**Abbreviations:** Ref. = Reference category; DLHS-4 = District-Level Household Survey 4; AHS = Annual Health Survey.

a All logistic models had diabetes, hypertension or obesity as outcome variables.

b The numbers in square brackets are 95% confidence intervals.

c These models included district household wealth quintile as independent variable.

d The subset was the 20% of the districts with the lowest primary school completion rate.

**Table 24:** Ordinary least squares regression of CVD risk factors onto household wealth (computed in each district) with district-level fixed effects (NFHS-4).^a^^,^^b^^,^^c^

		all districts		subset^d^	
		Absolute difference (% points)	P	Absolute difference (% points)	P
Household wealth quintile					
Diabetes	1 (poorest)	0.00 (Ref.)		0.00 (Ref.)	
	2	0.20 [0.07, 0.32]	0.002	−0.03 [−0.29, 0.22]	0.787
	3	0.57 [0.44, 0.69]	<0.001	0.48 [0.23, 0.73]	<0.001
	4	0.98 [0.86, 1.10]	<0.001	0.84 [0.59, 1.10]	<0.001
	5 (richest)	1.79 [1.66, 1.91]	<0.001	1.63 [1.38, 1.88]	<0.001
Hypertension	1 (poorest)	0.00 (Ref.)		0.00 (Ref.)	
	2	0.68 [0.40, 0.95]	<0.001	−0.02 [−0.59, 0.56]	0.956
	3	1.53 [1.26, 1.80]	<0.001	0.91 [0.34, 1.48]	0.002
	4	2.51 [2.24, 2.78]	<0.001	2.02 [1.45, 2.59]	<0.001
	5 (richest)	4.07 [3.79, 4.34]	<0.001	4.30 [3.72, 4.87]	<0.001
Obesity	1 (poorest)	0.00 (Ref.)		0.00 (Ref.)	
	2	1.83 [1.63, 2.03]	<0.001	0.85 [0.49, 1.20]	<0.001
	3	3.78 [3.58, 3.98]	<0.001	1.91 [1.56, 2.27]	<0.001
	4	6.03 [5.83, 6.24]	<0.001	3.82 [3.46, 4.18]	<0.001
	5 (richest)	9.94 [9.74, 10.14]	<0.001	8.11 [7.75, 8.47]	<0.001

**Abbreviations:** Ref. = Reference category; NFHS-4 = National Family Health Survey 4.

a All models had diabetes, hypertension or obesity as outcome variables.

b The numbers in square brackets are 95% confidence intervals.

c These models included district household wealth quintile as independent variable.

d The subset was the 20% of the districts with the lowest primary school completion rate.

**Table 25:** Ordinary least squares regression of CVD risk factors onto household wealth (computed in each district) with district-level fixed effects (DLHS-4/AHS).^a^^,^^b^^,^^c^

		all districts		subset^d^	
		Absolute difference (% points)	P	Absolute difference (% points)	P
Household wealth quintile					
Diabetes	1 (poorest)	0.00 (Ref.)		0.00 (Ref.)	
	2	0.32 [0.17, 0.46]	<0.001	0.04 [−0.23, 0.31]	0.790
	3	0.80 [0.66, 0.95]	<0.001	0.28 [0.01, 0.55]	0.046
	4	1.59 [1.45, 1.74]	<0.001	0.77 [0.50, 1.04]	<0.001
	5 (richest)	2.97 [2.82, 3.11]	<0.001	1.60 [1.33, 1.87]	<0.001
Hypertension	1 (poorest)	0.00 (Ref.)		0.00 (Ref.)	
	2	−0.28 [−0.52, −0.05]	0.017	−0.82 [−1.37, −0.28]	0.003
	3	0.60 [0.37, 0.83]	<0.001	−0.22 [−0.76, 0.32]	0.422
	4	1.50 [1.26, 1.73]	<0.001	−0.02 [−0.57, 0.52]	0.930
	5 (richest)	3.65 [3.41, 3.88]	<0.001	2.75 [2.21, 3.29]	<0.001
Obesity	1 (poorest)	0.00 (Ref.)		0.00 (Ref.)	
	2	1.70 [1.55, 1.85]	<0.001	0.51 [0.26, 0.76]	<0.001
	3	3.35 [3.20, 3.50]	<0.001	0.94 [0.69, 1.19]	<0.001
	4	5.47 [5.32, 5.62]	<0.001	2.07 [1.82, 2.32]	<0.001
	5 (richest)	9.24 [9.09, 9.39]	<0.001	4.81 [4.56, 5.06]	<0.001

**Abbreviations:** Ref. = Reference category; DLHS-4 = District-Level Household Survey 4; AHS = Annual Health Survey.

a All models had diabetes, hypertension or obesity as outcome variables.

b The numbers in square brackets are 95% confidence intervals.

c These models included district household wealth quintile as independent variable.

d The subset was the 20% of the districts with the lowest primary school completion rate.

Table 26, Table 27 show how the district-level independent variables are correlated.

**Table 26:** Correlation of district level indicator variables (NFHS-4).^a^^,^^b^^,^^c^

	% of participants who completed primary education	Median household wealth	GDP/capita	% of participants who live in an urban area	Female literacy rate
% of participants who completed primary education	1	0.72 [0.66, 0.77]	0.60 [0.53, 0.68]	0.50 [0.43, 0.56]	0.98 [0.96, 1.00]
Median household wealth	0.72 [0.66, 0.77]	1	0.68 [0.61, 0.74]	0.43 [0.36, 0.50]	0.68 [0.62, 0.74]
GDP/capita	0.59 [0.52, 0.66]	0.69 [0.63, 0.76]	1	0.60 [0.51, 0.69]	0.59 [0.52, 0.66]
% of participants who live in an urban area	0.49 [0.42, 0.55]	0.42 [0.35, 0.49]	0.43 [0.36, 0.50]	1	0.47 [0.40, 0.53]
Female literacy rate	0.96 [0.94, 0.98]	0.67 [0.61, 0.72]	0.60 [0.52, 0.67]	0.46 [0.39, 0.53]	1

a Ordinary least square regressions were used to conduct this analysis. The rows indicate the district-level indicators that were regressed onto the district-level indicators displayed in the columns.

b District-level variables (as continuous variables) were centered and scaled by subtracting the mean and dividing by two standard deviations prior to fitting these models.

c The numbers in square brackets are 95% confidence intervals.

**Table 27:** Correlation of district level indicator variables (DLHS-4/AHS).^a^^,^^b^^,^^c^

	% of participants who completed primary education	Median household wealth	GDP/capita	% of participants who live in an urban area	Female literacy rate
% of participants who completed primary education	1	0.78 [0.72, 0.84]	0.60 [0.52, 0.67]	0.60 [0.54, 0.67]	0.89 [0.85, 0.92]
Median household wealth	0.68 [0.63, 0.73]	1	0.63 [0.57, 0.69]	0.47 [0.40, 0.54]	0.53 [0.46, 0.59]
GDP/capita	0.62 [0.54, 0.69]	0.79 [0.71, 0.86]	1	0.49 [0.40, 0.57]	0.53 [0.46, 0.59]
% of participants who live in an urban area	0.61 [0.55, 0.68]	0.55 [0.47, 0.63]	0.41 [0.34, 0.49]	1	0.52 [0.45, 0.59]
Female literacy rate	0.92 [0.89, 0.96]	0.62 [0.55, 0.70]	0.54 [0.46, 0.61]	0.53 [0.45, 0.60]	1

Abbreviations: DLHS-4 = District-Level Household Survey 4; AHS = Annual Health Survey.

a Ordinary least square regressions were used to conduct this analysis. The rows indicate the district-level indicators that were regressed on the district-level indicators displayed in the columns.

b District-level variables (as continuous variables) were centered and scaled by subtracting the mean and dividing by two standard deviations prior to fitting these models.

c The numbers in square brackets are 95% confidence intervals.

In the sampling procedure, the health surveys used projections from either the 2001 or the 2011 India Census, while the GDP per capita data was collected in 2004/2005. Because of these time differences, we did not have GDP per capita data for some districts in each survey. We, therefore, excluded districts that were newly created within that time period (2001–2011) [2]. Neighboring districts, which underwent subsequent jurisdictional changes, were also excluded, leaving us with GDP per capita data for 476 of 640 districts in the NFHS-4 dataset and 467 of 561 districts in the DLHS-4/AHS dataset.

## 2 Experimental design, materials, and methods

Methods and statistical analyses are described in our main publication entitled “The interaction between district-level development and individual-level socioeconomic gradients of cardiovascular disease risk factors in India: A cross-sectional study of 2.4 million adults”. Here, we provide more detail on sampling procedure, anthropometric and biomarker measurements, construction of educational attainment categories, and the computation of household wealth quintiles. Analysis code files and raw data are provided in the Harvard Dataverse (link shown in the specifications table).

### 2.1 Sampling procedure and anthropometric and biomarker measurements

#### 2.1.1 National Family Health Survey (NFHS-4)

The NFHS-4 covered all 640 districts of India as of the time of the 2011 India census [3] and was conducted between 2015 and 2016. In the first stage of the stratified two-stage-cluster random sampling design, each district was separated into rural and urban areas and, within each rural or urban stratum, primary sampling units (PSUs) were selected with probability proportional to population size using the 2011 India census as a sampling frame. Rural PSUs were villages and urban PSUs were census enumeration blocks. In the following step, a household listing was carried out in the PSUs whereby large PSUs (defined as having more than 300 households) were divided into segments (each segment with approximately 100–150 households). Lastly, systematic random sampling (i.e., the first household was selected randomly, followed by the sampling of every nth household) was used in each PSU or PSU segment to select 22 households. Eligible women and men included all residents and visitors (who stayed the night prior to the survey) of the selected households. Women eligible for the women's survey were female residents or visitors that stayed the night prior to the survey and were 15–49 years old. The men's questionnaire was conducted in a random subsample of 15% of households. Eligible men were men aged 15–54 years who spent the night prior to the survey in the household or were usual residents. Men are, therefore, underrepresented in this survey and the variables for men that we used in this analysis are not representative at the district level. The socio-demographic data used in this analysis were ascertained by administering questionnaires using Computer Assisted Personal Interviewing (CAPI). Interviews with eligible women were completed with a response rate of 97%, while the response rate for eligible men was 92%. We only included non-pregnant residents (i.e. excluded pregnant women and visitors that stayed the night prior to the survey) in our dataset.

The biomarker questionnaire was administered to all eligible women and men and included measurements of height, weight, blood pressure, and blood glucose. For glucose measurements, capillary blood samples were taken with a finger prick and were analyzed with the FreeStyle Optimum H glucometer. The Omron Blood Pressure Monitor was used to measure blood pressure three times in the same arm in each individual, with a five-minute break in between measurements. Weight was assessed using the Seca 874 scale, and height measurements were conducted with the Seca 213 stadiometer. More information on the methodology of the survey and data collection procedures is available in the national report [4] and the NFHS-4 CAB manual [5].

#### 2.1.2 District-Level Household Survey-4 (DLHS-4) & Annual Health Survey (AHS)

The District-Level Household Survey–4 (DLHS-4) and the second update of the Annual Health Survey (AHS) were carried out simultaneously (between 2012 and 2014) and, when pooled, cover all Indian states except Gujarat and Jammu and Kashmir as well as all Union Territories except for Lakshadweep, and Dadra and Nagar Haveli. Sampling procedure and clinical, anthropometric, and biomarker (CAB) measurements are described elsewhere in detail and summarized below [6].

The DLHS-4 was conducted in 18 states and five Union Territories (comprising 336 districts in total) between 2012 and 2014 [7,8]. In the first stage of the two-stage cluster-random sampling design, PSUs were selected, which were “census villages” (sampled with probability proportional to population size using projections from the 2001 India census) in rural areas and “urban frame survey blocks” (selected through simple random sampling) in urban areas. Systematic random sampling was used in the second step to select the households in each PSU.

The AHS was conducted in nine states, comprising 284 districts between 2012 and 2013 [7,9]. These states were chosen because they had high percentages of infant and child mortality at the time of the conception of the first AHS. The two-stage cluster-random sampling approach was, again, stratified by rural versus urban areas. The PSUs were villages in rural areas and enumeration blocks in urban areas and both were selected through simple random sampling with probability proportional to population size using projections from the 2001 India census. Systematic random sampling was employed to choose households in each PSU. CAB measurements were conducted 12–18 months after the household questionnaire was conducted. Importantly, because sociodemographic information and CAB data in the AHS was published in the public domain in two separate datasets without a unique identifier that could be used to match participants across these two datasets, we had to resort to “fuzzy matching” to match individuals across these two datasets. Specifically, we merged participants using a composite indicator consisting of state, district, stratum (indicating rural versus urban areas and village size), a household identifier that is unique within each PSU, and a household serial number assigned before and one assigned after data entry. 59.0% (607,227 out of 1,028,545 participants) of non-pregnant adults in the CAB dataset were successfully merged to their corresponding sociodemographic information. Those whom we could not match had similar sample characteristics as those whom we were able to match; detailed tables of this comparison are shown in the appendix of our first publication with this data [6].

CAB measurements were conducted in all adult non-pregnant household members. Visitors were excluded from our dataset. Trained data collectors quantified blood glucose from a finger prick blood specimen with a handheld glucometer (SD CodeFree), which automatically converted capillary blood glucose readings into a plasma-equivalent value by multiplying with 1.11 [10]. Participants were instructed to fast overnight before blood glucose was measured the following morning. Blood pressure was measured with an electronic blood pressure monitor (Rossmax AW150) in the upper arm when the participant was sitting. Blood pressure measurements were repeated twice with a ten-minute interval between readings. A household questionnaire was used to ascertain the socio-demographic information that was used in our analysis. The respondent was the household head, who answered on behalf of all household members.

A more detailed description of the sampling procedure and data collection procedures is available in the state reports [8,9] and the CAB manual [11].

### 2.2 Measures of socio-economic status (SES)

We used educational attainment and household wealth as individual-level SES measures. Table 28 shows the ordinary least squares regression of household wealth onto educational attainment.

**Table 28:** Ordinary least squares regression of household wealth (computed in each district) on educational attainment.^a^

Educational attainment	*NFHS-4*		*DLHS-4/AHS*	
	Absolute difference (% points)	P	Absolute difference (% points)	P
< primary	0.00 (Ref.)		0.00 (Ref.)	
Primary completed	36.03 [34.77, 37.28]	<0.001	27.99 [27.29, 28.69]	<0.001
Some secondary	72.07 [71.37, 72.76]	<0.001	59.41 [58.89, 59.93]	<0.001
Secondary completed	122.25 [121.14, 123.37]	<0.001	94.53 [93.78, 95.27]	<0.001
> secondary	163.54 [162.54, 164.54]	<0.001	135.72 [134.99, 136.46]	<0.001

**Abbreviations:** Ref. = Reference category; NFHS-4 = National Family Health Survey 4; DLHS-4 = District-Level Household Survey 4; AHS = Annual Health Survey.

a The numbers in square brackets are 95% confidence intervals.

The household wealth quintile of DLHS-4 and AHS respondents was constructed as previously described [12]. Shortly, the household wealth quintiles were created by dividing a continuous household wealth index variable into quintiles, either at the district or national level. At the national level, this was done separately for rural and urban areas.

If the urban or rural proportion in a district was ≥5%, the computation of wealth quintiles at the district level was also performed separately for urban and rural areas. The continuous household wealth index was the standardized (to yield a mean of zero and standard deviation of one) first principal component from a principal component analysis (PCA) of binary variables, which indicated household ownership of durable goods and key housing characteristics (coded each as 1 or 0) [13]. The PCA was conducted separately for urban and rural areas.

The household wealth quintiles for NFHS-4 respondents were created using the same methodology. A more detailed description of the construction of the wealth indices in the NFHS-4 is provided by the DHS program [14]. The assets (ownership of durable goods) and key housing characteristics that were used to construct the household wealth index in each survey are listed in Table 29.

**Table 29:** Variables used to construct the household wealth index.

DLHS-4/AHS	NFHS-4
Improved water supply	Source of drinking water
Improved sanitation facility	Type of toilet facility
Cooking fuel	Type of cooking fuel
House structure	
Source of lighting	
Ownership of house	Ownership of house
Land	Ownership of land
	Main material of floor
	Main roof material
	Main wall material
	Ownership of animals
	Number of members per sleeping room
	Domestic staff listed in household
	Bank account
Radio	Radio or translator
TV	Black and white television
	Colour television
Phone	Mobile telephone
	Telephone (non-mobile)
Fridge	Refrigerator
Bike	Bicycle
Scooter	Motorcycle or Scooter
Car	Animal-drawn cart
	Car
Computer	Computer
Washing machine	Washing machine
Sewing machine	Sewing machine
	Electricity
	Mattress
	Pressure cooker
	Chair
	Cot or bed
	Table
	Electric fan
	Internet
	Air conditioner/cooler
	Watch or clock
	Water pump
	Thresher
	Tractor

The construction of educational attainment categories is presented in Table 30.

**Table 30:** Construction of educational attainment categories.

Educational attainment variable	NFHS-4 answers	DLHS/AHS answers
Below primary (Some primary)	No education; Incomplete primary	Illiterate; Literate without formal education; Below Primary
Primary	Primary	Primary
Some secondary	Incomplete Secondary	Middle; Secondary/Matric (class-x)
Secondary completed	Complete Secondary	Hr. Secondary/Sr. Secondary/pre University (class xii)
Higher (>Secondary)	Higher	Graduate/B.B.A/B.Tech/ MBBS/equivalent; Post graduate/M.B.A/MCA/equivalent or higher; Technical Diploma; Non-technical diploma or certificate not equivalent to degree
